# Possible causes of data model discrepancy in the temperature history of the last Millennium

**DOI:** 10.1038/s41598-018-25862-2

**Published:** 2018-05-15

**Authors:** Raphael Neukom, Andrew P. Schurer, Nathan. J. Steiger, Gabriele C. Hegerl

**Affiliations:** 1 Oeschger Centre for Climate Change Research and Institute of Geography, University of Bern, Bern, Switzerland; 2 School of Geosciences, University of Edinburgh, Edinburgh, UK; 3 Lamont-Doherty Earth Observatory, Columbia University, Palisades, New York, USA

## Abstract

Model simulations and proxy-based reconstructions are the main tools for quantifying pre-instrumental climate variations. For some metrics such as Northern Hemisphere mean temperatures, there is remarkable agreement between models and reconstructions. For other diagnostics, such as the regional response to volcanic eruptions, or hemispheric temperature differences, substantial disagreements between data and models have been reported. Here, we assess the potential sources of these discrepancies by comparing 1000-year hemispheric temperature reconstructions based on real-world paleoclimate proxies with climate-model-based pseudoproxies. These pseudoproxy experiments (PPE) indicate that noise inherent in proxy records and the unequal spatial distribution of proxy data are the key factors in explaining the data-model differences. For example, lower inter-hemispheric correlations in reconstructions can be fully accounted for by these factors in the PPE. Noise and data sampling also partly explain the reduced amplitude of the response to external forcing in reconstructions compared to models. For other metrics, such as inter-hemispheric differences, some, although reduced, discrepancy remains. Our results suggest that improving proxy data quality and spatial coverage is the key factor to increase the quality of future climate reconstructions, while the total number of proxy records and reconstruction methodology play a smaller role.

## Introduction

Knowledge of past climate variability is important to put contemporary climate states and changes into a long-term context. Furthermore, paleoclimate studies can help to evaluate the ability of climate models to realistically simulate the relative strength of externally forced versus internally driven influences on the climate system. The last millennium is important for such assessments, as proxy data for both climatic fluctuations and external forcing factors (such as volcanic eruptions, solar irradiance and greenhouse gas concentrations) are available in relatively high temporal and spatial resolution. Particularly for temperature, a wide range of proxy based reconstructions on regional to global levels exist^1–5^.

For Northern Hemispheric (NH) mean surface air temperatures, a wealth of reconstructions is available^1,4,6^. These reconstructions are in remarkably good agreement with independent climate model simulations in terms of amplitudes and multi-decadal variability after ca. 1400^1^. In contrast, there is some disagreement, for example with regards to timing, amount and spatial extent of relative warmth during medieval times^1,7,8^. Current understanding suggests that volcanic eruptions and greenhouse gas concentrations are the main drivers of NH temperature variability over the last Millennium and that solar variability plays a minor role^1,9,10^.

**Figure 1:**
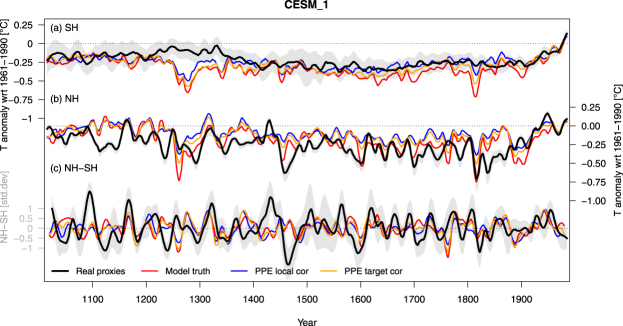
Comparison of hemispheric model temperatures, pseudoproxies and real proxy reconstructions over the past Millennium. Real proxy temperature reconstructions (black, grey shaded area indicating: 90% ensemble range), model truth (red) and pseudoproxy reconstructions using synthetic proxies based on local (blue) and hemispheric mean (yellow) correlations for the CESM1-CAM5 (member 1) simulation over the past Millennium. (**a**) SH, (**b**) NH and (**c**) standardized NH-SH difference. A 30-year loess filter was applied to all curves. Data in (**a**) and (**b**) are centered on the climatological base period of 1961–1990.

**Figure 2:**
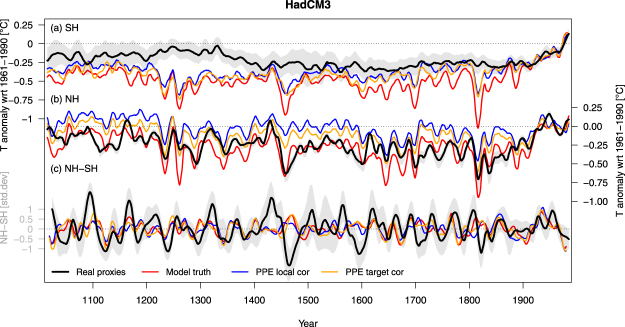
Same as Fig. 1 but for the HadCM3 last millennium simulation. Plots for all other simulations in the SM (Supplementary Figs 44–55).

However, comparisons of reconstructions on continental to hemispheric scales suggest substantial differences in decadal to centennial temperature variability across regions, particularly, in the Southern Hemisphere (SH), that cannot be reproduced by model simulations^2,10–12^. For instance, the correlations between regional and hemispheric temperatures are much weaker in reconstructions than simulations^12^. The global timing of decadal-scale extreme periods was found to be less tied to strong peaks of volcanic forcing in reconstructions compared to models^11^. Furthermore, inter-hemispheric temperature differences appear larger (Figs 1c, 2c and ref.^11^) and industrial-era warming more delayed in some regions^10^ in reconstructions than in simulations. Finally, detection and attribution results for the SH for the last millennium suggest a surprisingly small response to forcing in reconstructions^12^. These data-model discrepancies may arise from model deficiencies in simulating the relative influence of external forcing and internal variability on the climate system, leading to imperfect multidecadal and regional- to large-scale variability in the model^13–15^. They might also arise from insufficient quality or distribution of proxy data, noise in proxy data, deficiencies in reconstruction methods, or a combination of all these factors.

This study uses pseudoproxy experiments (PPE; refs^16,17^) to investigate potential sources of these data-model discrepancies. PPE have been widely used to evaluate climate reconstruction methods^7,16–23^. PPE use the virtual reality of climate model simulations, which provide physically self-consistent and complete global datasets of possible climate over the entire Millennium. The model output at real-world proxy locations is used in PPE to generate synthetic pseudoproxies. Typically, this is done by adding a realistic amount and type of noise to simulated grid-cell temperatures^17^. Such noise-based pseudoproxies do not capture non-temperature variations and nonlinear relationships between proxy and climate variables. The latter can be explored using physically-based proxy system models and multi-variable inputs from climate models^24–29^.

The pseudoproxies are processed in the same way as actual proxies to calculate a climate reconstruction, which can be compared to the target climate variable derived from the same climate model. Any significant difference between the pseudoproxy reconstruction and model truth is then due to a combination of proxy noise, sub-sampling of the model field using proxy locations, and the limitations of the reconstruction method itself. Ref.^17^ presents a comprehensive overview of the concepts, history and applications of PPE. Note that while PPE are a powerful tool to evaluate climate reconstructions, PPE results can vary with the climate model used, and depend on choosing noise with adequate magnitude and autocorrelation structure^6,7,17,22^.

In the past, PPE have been mostly used to compare reconstruction methods and investigate differences between the model truth and the reconstructed climate in metrics such as the amplitude between temperatures of the Medieval Climate Anomaly, the Little Ice Age (LIA) and present-day warmth^7,17,22^.

Here, we use PPE to investigate possible causes of differences in interhemispheric temperature contrast and response to forcing between models and reconstructions. We generate real proxy and pseudoproxy reconstructions of NH and SH mean temperatures over the past 1000 years following the data selection and methods of two recent hemispheric reconstructions^5,11^. We use 14 simulations from two CMIP5/PMIP last millennium climate models (HadCM3^30^ and CESM1-CAM5^31^) and multiple methods to generate pseudoproxies to test the influence of datasets and methodological choices on the results (see Methods). PPE and real-world temperature reconstructions are used to evaluate the influence of proxy noise, proxy site distribution and reconstruction methodology on a number of key metrics, for which data-model discrepancies have been diagnosed in the past: NH vs. SH correlations, NH-SH differences, the response to volcanic eruptions, and the magnitude of the response to forcing based on formal detection and attribution.

## Results

### Performance of pseudoproxy and real-world reconstructions

Hemispheric temperature reconstructions were generated using the same methods as in the original publications, with some minor differences in the parameters (see Methods): Principal component regression (PCR) for the SH^11^ and Composite Plus Scale (CPS) for the NH^5^. Both are relatively simple linear methods that usually yield very similar results^5,11,32,33^. Note that to allow direct comparison of our results with earlier studies^11,34^, we use twelve month seasonal windows and full hemispheric mean targets (as opposed to summer and extratropical land-only in ref.^5^). However, our interpretations are robust with regards to the choice of the NH proxy data and target season and domain (Supplementary Figs 6–9 and 28). The hemispheric reconstructions based on real proxy data are shown in Figs 1 and 2 (black lines).

We calculated a range of PPE reconstructions based on statistically derived pseudoproxies using different structures and amplitudes of noise (see Methods and Supplementary Section S3). In a first step, the model field is subsampled at the locations of the proxy records in the SH^11^ and NH^5^ (Supplementary Fig. 1). These grid-cell time series from the model simulations are then subjected to the reconstruction methods to estimate hemispheric mean temperatures (Perfect pseudoproxies, *NoNoise* experiment).

To obtain more realistic pseudoproxies, we then add noise to the model grid-cell temperatures. We generate a range of nine white and AR1 noise experiments (Table S1) with varying signal to noise ratios (SNR), both idealized and designed to reproduce the correlation to local or target instrumental data and autocorrelation structure of the real-world proxy data. We select the experiments with reconstruction skill closest to the real-world reconstructions as “best match” PPE (see Methods & Table S2), and keep the other experiments for sensitivity tests (Supplementary Figs 10, 11, 17–22, 26 and 56–83). Reconstruction skill is estimated by comparing the real-world reconstructions with measured instrumental data and the PPE with the model output over a validation period independent from calibration using the root mean squared error (RMSE), correlation coefficient and reduction of error (RE) validation metrics^35^ (see Methods). Our conclusions are robust with regards to the choice of the best match PPE (Supplementary Fig. 26). Additionally, we generate an alternative set of PPE using physically-based proxy system models (PSM) following the approach of ref.^29^ for creating realistic tree-ring width and coral δ^18^O pseudoproxies, two proxy types that dominate global proxy networks^36^ (details see Supplementary Section S4). We find that the PSM-based results are very similar to the ones using statistical pseudoproxies (Supplementary Figs 12–16) and so we focus the discussion of our results on noise-based pseudoproxies. This similarity of results suggests that while nonlinear and multi-variable relationships between proxy data and climate may occur, they are not the dominant cause of the data-model discrepancies assessed herein.

Interestingly, the PPE that most closely resembles the skill of the real-world reconstructions is not the realization with the most realistic SNR based on real-world correlations of proxy data with local temperatures, but one with relatively small amounts of noise (SNR by standard deviation of 1; *LocalCor* experiment). In other words, the real-world reconstructions perform better than one would expect based on the local noise. The weaker performance of the PPE compared to real proxies can possibly be explained by the lower correlations of local grid-cell temperatures with the hemispheric mean reconstruction target in models compared to real-world data^6^. For example, the median interannual correlation of instrumental temperatures at the locations of the proxy data with the NH mean is r = 0.37 (Supplementary Fig. 2), but varies between r = 0.19 and r = 0.31 for the equivalent median correlation in the individual model ensemble members used herein. These values are not systematically different for the HadCM3 and CESM1-CAM5 model members (average 0.26 for both models). For the real proxy data, the median correlation with the NH mean over the instrumental period is r = 0.21 (Supplementary Fig. 2) while that value is substantially lower, r = 0.04 to r = 0.16, for the model-based pseudoproxies with realistic local temperature correlations.

In order to account for the reduced co-variability of local temperatures with the hemispheric mean in the model world, we also generated pseudoproxies that replicate the correlations of the proxy with the field mean target instead of local temperatures^6^ (*TargetCor* experiment, see Methods and supplementary material SM). We stress that this is a sensitivity experiment addressing the too-strong local noise in models, and we do not assume a direct mechanistic relationship between hemispheric temperature and proxy data. It is important to note that the relationship of local temperatures to the hemispheric mean is not constant over time in the model simulations, which probably affects the PPE. However, the average correlation of NH model proxy locations to the NH model mean over the last Millennium (r = 0.27) is almost exactly the same as over the calibration period (r = 0.26, Supplementary Fig. 3).

**Figure 3:**
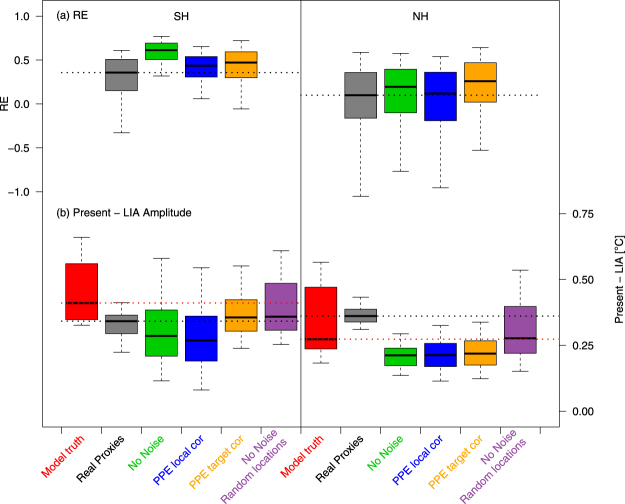
Reconstruction skill and low frequency amplitude. (**a**) Temporal mean Reduction of Error (RE) skill, in the SH (left) and NH (right). Higher values indicate higher reconstruction skill. (**b**) Low-frequency amplitude defined as the difference between average temperatures over 1950–1999 (present-day) and 1600–1649 (LIA). Boxplots are across all model simulations and reconstruction ensemble members. Dashed black (red) horizontal lines are the median values of the real proxy experiments (model truth). Bold lines are medians, boxes represent the interquartile range and whiskers the 90% range. Additional skill metrics and PPE as well as the results from each simulation are shown in the SM.

Example PPE reconstructions from two simulations for the *LocalCor* (blue color in all Figures) and *TargetCor* (yellow) experiments are shown in Figs 1 and 2; and their RE skill is compared to the real proxy results (black) and the *NoNoise* case (green) in Fig. 3a. The performance of the *NoNoise* experiment is over-optimistic in the SH (higher RE values than the real proxies), but similar to the real proxies and noise cases in the NH. This indicates that in the NH, much of the uncertainty in the reconstruction is introduced by the sub-sampling at proxy locations, with little effect of local noise. In the SH, subsampling alone generates too skillful reconstructions compared to reality and noise is required to generate realistic pseudoproxy experiments.

### Low frequency variability

In previous studies, PPE have suggested an underestimation of low frequency variability in proxy based reconstructions^6,17,22^. We therefore compare the temperature amplitude between LIA (1600–1649 CE) and present-day (1950–1999 CE) conditions in real proxy and pseudoproxy reconstructions with the model “truth” (red) in Fig. 3b. For the SH, the reconstructed amplitude from real-world proxies is smaller than in the models. In previous studies, this has been attributed to deficiencies in the PCR reconstruction method^4,19^, or imperfect representation of low frequency variability in the proxy records or models. In the NH, the low frequency amplitude of the real proxy reconstructions is well within the range of model simulations, which is in contrast to most earlier studies, usually finding an underestimation of amplitude by reconstructions relative to model experiments^17^. This discrepancy may be explained by the fact that the CESM and HadCM3 models used herein have a small present-day – LIA amplitude compared to other models^12,31^. A plausible cause for this reduced amplitude is anthropogenic aerosol forcing, which is relatively strong in the models used herein, compared to most other last-millennium simulations that are currently available. Aerosols tend to preferentially cool the NH, and because of this, the CESM and HadCM3 simulations also have a larger present-day – LIA amplitude in the SH than in the NH (Fig. 3b).

The *NoNoise* PPE have reduced present-day – LIA amplitude compared to the model truth in both hemispheres, but the magnitude of the amplitude loss is larger in the SH (median of 31%) than in the NH (22%). This is probably due to the reduced number of proxies available during the LIA period in the SH (27 records) compared to the NH (46 records). The PPE with realistic noise levels shown in Fig. 3 do not display further amplitude loss compared to the *NoNoise* cases in either hemisphere. This is in contrast to PPE with higher amounts of noise (sensitivity experiments, Supplementary Figs 17–22), which have further reduced amplitudes. Accordingly, the loss of low frequency amplitude in the realistic PPE compared to the model truth is either caused by the reconstruction methodology or the subsampling of the field at the given proxy locations.

Alternative reconstruction methods that aim at retaining the variance better (Pairwise Comparison PaiCO^37^ and Bayesian Hierarchical Models BHM^38^), are able to generate larger amplitudes for some experiments (Supplementary Figs 33–38). However, the results are not consistent across the hemispheres and do not allow to identify a single method that is superior to the others. Overall, SH reconstructions appear to be more sensitive to the choice of the reconstruction method.

In order to evaluate the effect of the proxy spatial distribution, we generated additional experiments using the same number of pseudoproxies as in reality, but sub-sampled the model field at random locations. Resulting pseudoproxy-reconstructions (purple color in all Figures) show increased amplitude compared to the original *NoNoise* PPE in both hemispheres (Fig. 3b), indicating the non-homogeneous distribution of proxy data is a primary cause for amplitude losses. Further increasing the number of randomly sampled proxy locations has a positive but much smaller effect on amplitude losses (Supplementary Figs 23 and 24). Overall, variance losses are larger for the HadCM3 simulations, where the amplitude of the model truth is larger than in CESM (Figs 1, 2, S5–S8).

To summarize, low-frequency variance losses in the PPE are mainly caused by number and location of the proxy sites, but also depend on the climate model and reconstruction method chosen, particularly if the number and quality of available proxies is low. These results are in line with a recent study assessing the influence of method and proxy location in the extratropical NH^6^. In contrast, the amount of noise typically inherent in the proxy data appears to only have a minor influence, contrasting findings in ref.^6^, where a large influence of local proxy correlations on amplitude is found and relatively small amplitude losses in the *NoNoise* case given the number of proxies used herein. This apparent inconsistency may be partly caused by the different input datasets and climate models chosen (relatively low correlations of local temperatures with the hemispheric mean in the models used herein). Also, the magnitude of noise in our “best match” PPE is relatively low compared to the typically chosen SNRs, but targeted to reflect the real-world situation. Our results confirm earlier findings that the true amplitudes in both hemispheres may be higher than in the real-world reconstructions^6,17,22^.

### Inter-hemispheric correlations and differences

**Figure 4:**
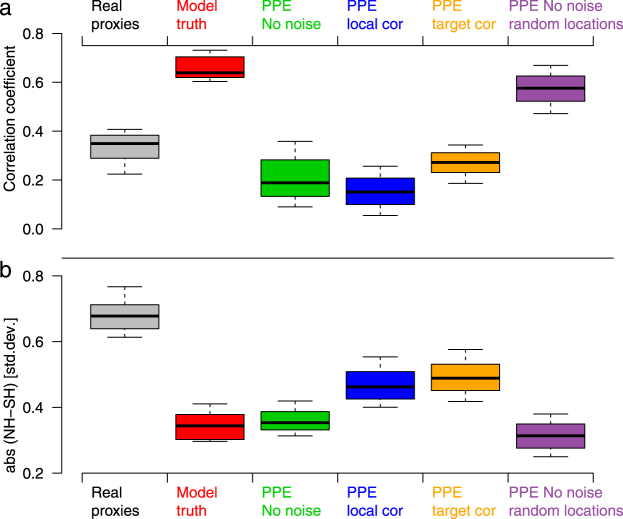
Inter-hemispheric correlations and differences. (**a**) Correlations between the NH and the SH over the period 1400–1999. Distributions are shown across all model simulations and ensemble members. (**b**) same but for NH-SH differences. Boxplots are defined as in Fig. 3.

There are substantial differences in inter-hemispheric correlations across the reconstruction experiments. Fig. 4a shows that while the model truth has much higher correlations than the real proxies, most PPE show inter-hemispheric correlations that are more consistent with the real proxies, with even lower median values. Comparable to the present vs. LIA differences, subsampling the model at proxy locations (purple boxes in Fig. 4) appears to have the largest effect, with added noise (blue and yellow boxes) only slightly further reducing correlations (note that the amplitude of the reconstruction anomalies is disregarded in correlations). Similar results are obtained with alternative reconstruction methods (Supplementary Figs 39 and 40). Consistent with results on low-frequency variance, the discrepancy between results for original and randomly sampled proxy locations is largest for the NH, while the use of random sampled locations in the SH alone has a minor effect on correlations (not shown).

The lower correlations between the hemispheres in reconstructions compared to models identified in previous studies^11,12^ can thus be fully explained by the PPE and are largely caused by the unequal spatial distribution of proxy data.

In contrast to the correlations, discrepancies between models and data in their decadal-scale inter-hemispheric temperature difference can only partly be explained by the PPE. Results for the *NoNoise* PPE are very similar to the model truth (Fig. 4b), suggesting that sub-sampling of the field plays a minor role for this metric. While increasing noise moves the distributions closer to values of the real proxies, all PPE still show lower magnitudes in NH-SH differences (example time series in Figs 1c and 2c). Our most realistic PPE indicate that about 50% of the difference between the reconstructions and the model truth can be explained by proxy noise. Again, the results are robust to the choice of reconstruction method (Supplementary Figs 39 and 40) and pseudoproxy type (Supplementary Fig. 14); suggesting that variance loss in reconstruction methods does not influence hemispheric differences. Instead, strong decadal-scale events of inter-hemispheric temperature contrast caused by internal variability may be responsible e.g. as observed in the 1970s^11,39^, and may be underestimated in magnitude by model simulations.

Similar to the NH-SH differences, the timing of extreme decades over the last 1000 years remains mostly unaffected by the different noise levels or reconstruction methods (Supplementary Figs 27 and 41). Cold peaks are uniquely associated with large volcanic eruptions in all PPE, whereas the real reconstructions have the coldest period in the 17^th^ century, where the modelled extremes are moderate.

### Volcanic cooling

**Figure 5:**
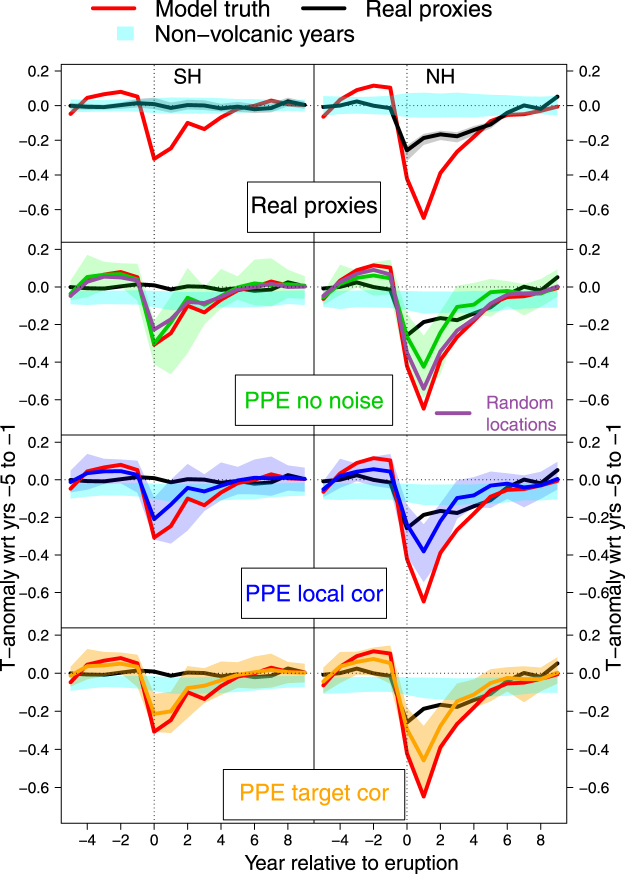
Superposed Epoch Analysis of the temperature response to volcanic eruptions. SH (left) and NH (right) temperature anomalies relative to 5-year pre-eruption means. Lines (shading) represent ensemble medians (9–95% range). Red: model truth, black: real proxies, other colors: PPE. Cyan shading is the 5–95% range of monte-carlo sampled years during non-volcanic periods. Distributions are across all model simulations and ensemble members. Model truth and real proxy data are shown in all panels for better comparison.

To evaluate if noise in proxy data can explain the small volcanic response in particularly the SH proxy reconstructions^11,40^, the temperature response to volcanic eruptions is assessed using superposed epoch analysis^41–43^ (SEA) for the different PPE in the SH (Fig. 5, left) and NH (right), using the largest 14 eruptions within the last Millennium^44^ (see Methods). The cooling response in the model truth is reduced in magnitude by 50% in the SH compared to the NH and peaks already in the year of the eruption, whereas in the NH maximum cooling is found in the year after the eruption. The smaller response in the SH compared to the NH in both reconstructions and models may be due to the larger fraction of oceans in the SH damping the response to volcanic forcing. Note that the magnitude of the response depends on the number and size of events selected for the analysis, so while there is good qualitative agreement with other studies (e.g. refs^1,5^), the magnitude of the response differs slightly.

The real proxy reconstructions show no clear response in the average volcanic response in the SH. In the NH, the reconstructed cooling response is significant, but reduced by 0.40 °C compared to the models (ensemble medians). The response in our data is weaker than in the reconstruction of ref.^5^, because we use a hemispheric mean target in contrast to the land-only target in ref.^5^. Scaling the latter reconstruction to our combined land-ocean target yields results that are practically identical to ours (Supplementary Fig. 28). The ability of tree-ring records to capture volcanic cooling and differences to models and instrumental measurements have been discussed widely^5,9,45–50^. Here, we focus on the difference between the hemispheres and comparison between real-world and pseudoproxy reconstructions.

In the SH, the cooling in the model truth and *NoNoise* experiments are practically identical, indicating that the proxy locations are representative for the hemispheric mean. This is in contrast to the NH, where the *NoNoise* PPE shows a reduced cooling compared to the model truth. Again, the reconstruction method does not play a critical role for these results (Supplementary Fig. 42).

As in the other diagnostics, only in the NH do the proxy locations explain part of the model-data discrepancy: using randomly sampled locations in the *NoNoise* PPE accounts for 52% of the reduced signal compared to the model truth in the NH; (Fig. 5). Adding more proxies to the randomly sampled NH networks does, again, have a very minor influence on the results (Supplementary Fig. 29).

The change in the volcanic response by adding noise to the pseudoproxies is very small in the NH (Fig. 5). In the SH, in contrast, the noise PPE reduce volcanic cooling by about 33% compared to the *NoNoise* PPE. The reconstructions using alternative methods (PaiCO and BHM) perform better in the SH noise PPE, having a similar or even larger signal than the model truth (Supplementary Fig. 42). In the real-world, however, reconstructions based on all methods show a very weak or no response. Thus, while the ‘standard’ reconstruction methods slightly reduce the volcanic signal in PPE, neither proxy noise or distribution, nor reconstruction method can fully explain the non-response seen in the real-world reconstructions in the SH. The strong modulating effect of the ocean or counter-acting response of key modes of internal variability such as ENSO^51–55^ or SAM^56^ may contribute to the large model-data discrepancy in the SH, as they may be imperfectly simulated by models^55^. Alternatively, a response in the hydrological cycle opposite to the temperature response^55,57,58^ may compensate the cooling response in some real world proxies, but not in our temperature-based pseudoproxies. Note that the spatial patterns of such a hydrological signal may be different in the real and model worlds. Also, as there are no tree-ring density records available for the SH, which tend to show a larger volcanic signal than width-based records, the detection of volcanic cooling may be limited by proxy archive availability^40^. Finally, timing and location of an eruption^59–61^ and the chemical composition of each eruption’s plume^14^ play an important role; and therefore the currently available forcing reconstructions may not be sufficiently detailed to allow models to capture regionally different responses^62^. All these factors may contribute to the non-response of real-world reconstructions in the SH and to the data-model discrepancies.

In our PPE, the results for the white and AR1 noise experiments with identical SNRs are nearly the same (Supplementary Figs 10, 11 and 17–22, see also ref.^23^). Thus using realistic AR1 coefficients in the pseudoproxies does not alter the response to volcanic eruptions. Note that our PPE do not account for higher order autocorrelation and thus the possibility of higher memory in proxy data than instrumental temperatures on longer time-scales^63^.

### Detection & Attribution

Detection and attribution (D&A)^64,65^, has been used to disentangle the climatic response to external forcings (such as volcanic eruptions and solar variability) from chaotic internal variability and each other e.g.^20,30^. D&A studies typically use a multivariate Total Least Squares (TLS) regression technique to regress forced “fingerprints” of change taken from the mean of several model simulations onto reconstructions of past temperature change. This results in ‘scaling factors’ that quantify the magnitude of the forced response with an associated uncertainty range, estimated using unforced model control simulations (see Methods). If a scaling factor is found to be significantly greater than 0 (p > 0.05) then the effect of the forcing is “detected”, and can be said to be consistent with the actual model response if the uncertainty range encompasses 1. Earlier work^9,12^ found that a forced response can been detected in all regions of the NH but only for few regions and time periods in the SH. Many reconstructions yield scaling factors that are significantly less than 1 for either volcanic forcing, or all forcings combined; indicating a significantly stronger response to forcing in models than in the reconstructions.

**Figure 6:**
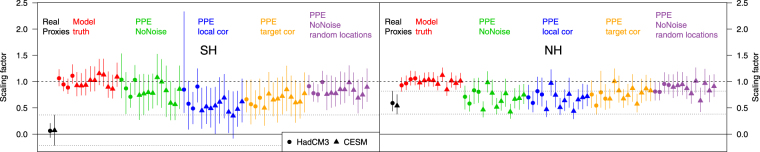
Detection and Attribution scaling factors. Amplitude (‘scaling factor’) of the response to all-forcing fingerprints in real proxies and pseudoproxies for the SH (left) and NH (right) over the period 1400–1900. Circles (triangles): HadCM3 (CESM-LME) ensemble members. Symbols represent the ensemble median, vertical lines the 90% range.

Here we investigate, for the first time, the effect of proxy noise on D&A results and whether the proxy noise could be masking the influence of external forcing in temperature reconstructions. We carry out a typical D&A type perfect-model experiment using the HadCM3 (circles in Fig. 6) and CESM1-CAM5 (triangles in Fig. 6) model simulations. Consistent with ref.^12^ for the pre-industrial period (1400–1900), we detect the effect of external forcings in the NH real-world reconstructions (scaling factors significantly greater than 0) but not in the SH (scaling factor range encompasses 0). If the 20^th^ century is included in the analysis, external forcing is now detectable in the SH (Supplementary Fig. 30), presumably due to strong warming at the end of the record in both models and reconstructions.

To assess the influence of reconstruction method and proxy noise in the model and PPE domain, we use one of the model ensemble members in place of the real-world reconstructions and regress the mean of the remaining model ensemble members onto this. Such a set-up will allow us to investigate the effect of various assumptions about proxy noise in a situation where the true answer is known, since by construction the scaling factor will be consistent with ‘1’ if using the model truth (see Fig. 6).

In the NH, the scaling factors of the *NoNoise* experiments are significantly less than 1 in the majority of cases and comparable to that calculated using the real proxies. Using random location increases the scaling factors, thus, once again the sub-sampling of the field plays a key role to explain the low scaling factors for external forcing discussed in the literature, while adding additional noise to the pseudoproxies has only a minor influence on the results. These conclusions are very similar to those reached for the response to volcanic eruptions discussed above. This is because the results for the D&A experiment during the pre-1900 period are mostly driven by volcanic forcing, (the average volcanic cooling shown in Fig. 5 explains 67% of variance in the scaling factors in the NH; Supplementary Fig. 31).

In the SH, scaling factors from the *NoNoise* experiment indicate a reduced amplitude but by less than in the NH and with little change using random locations, again consistent with results for the epoch analysis. Adding proxy noise reduces the scaling factors of the PPE, but they remain higher than the results of the real proxies over 1400–1900, which are not distinguishable from zero, meaning no detectable influence of external forcing on reconstructed temperatures. The *LocalCor* (*TargetCor*) experiments in the SH yield signal amplitudes that are compatible with the real-world proxies for 12 (7) out of 14 simulations and significantly larger for the other 2 (7). On average the scaling factors are 0.57 (*LocalCor*) and 0.67 (*LocalCor*), thus roughly in the middle between the model truth and real proxies. This suggest that about 50% of the apparent non-response of SH reconstructions to external forcing can be explained by noise in the proxy data. Reconstruction method does also play a role in both hemispheres (Supplementary Fig. 43) but again no discernible best method can be identified.

## Discussion

Our results corroborate the importance of PPE to evaluate climate reconstructions and their interpretation. However, we find that the signal-to-noise ratios typically chosen based on the local correlations of proxy records with the instrumental data tend to underestimate the quality of real-world reconstructions in terms of validation skill. A main reason for this discrepancy is the different behavior of local vs. large-scale temperatures in climate model and instrumental data. The identification of “optimal” noise levels is difficult as the performance of the various PPE varies between different metrics, e.g. reconstruction skill versus low frequency amplitude. This suggests that either aspects of the proxy autocorrelation or large-scale / small-scale relationship is different between real-world and model data.

Although our conclusions are independent from the type of pseudoproxies (noise-based vs. proxy system models), future use of more elaborate proxy system models^26–28^ or reconstruction methods that make direct use of such models^28,29^ may allow a more detailed assessment of the influence of spectral properties and other variables (e.g. hydroclimate, CO_2_) on the data-model discrepancies discussed herein.

Different factors appear to be influencing reconstruction quality in the two hemispheres. Imperfect sub-sampling of the field by the proxies is the main cause of data-model discrepancies in the NH for most diagnostics studied herein (low frequency variability, inter-hemispheric correlations and response to forcing). In the SH, where proxy records are of lower quality and number, both proxy noise and reconstruction methodology appear to play a role. While the choice of reconstruction methodology influences the temperature amplitude in the reconstruction period, its effect on NH vs. SH correlations and differences and the timing of extreme periods is minor^11,33^. The response to forcing is also influenced by the reconstruction method but our results do not allow to identify a method that is superior to the others in all metrics, experiments and hemispheres.

*NoNoise* PPE tend to validate better in the SH than in the NH, most probably due to the stronger average relation of local temperature with the hemispheric mean caused by the strong oceanic influence in the SH. Although this advantage is currently overcompensated by the reduced number, average length and spatial coverage of proxy records in the SH^36,66^, it is encouraging for future attempts to reconstruct SH mean and regional climate.

Our results carried out in the controlled conditions of PPE indicate that noise and proxy distribution can fully explain some of the discrepancies between models and data (e.g. correlations and D&A results in the NH) and others only partly (e.g. NH-SH differences, forcing response in the SH). However, none of our PPE can consistently explain all discrepancies, suggesting that there are further contributing factors to model-data differences. These may include model deficiencies, forcing errors, or additional uncertainties in climate reconstructions. The non-response of current SH reconstructions to volcanic eruptions is plausible given our PPE results, if further considering the lack of high-quality proxies such as tree-ring density measurements and the moisture and large-scale circulation changes possibly counteracting the cooling response in some regions^53,55–58^. This is again an aspect that may be further investigated using process based proxy models.

The relatively large inter-hemispheric differences in the reconstructions and the timing of cold and warm peaks cannot or only partly be explained by the PPE. This suggests that internally driven changes in circulation^39,67^, or circulation response to forcing that is not captured by climate models, may have influenced regional to hemispheric climate on decadal and longer time-scales, with a magnitude comparable to externally forced extremes^68,69^. Ref.^70^ showed evidence that such regional differences do not necessarily require an overestimation of external forcing response by the models, because the regional differences can be consistent with model physics. Alternatively, the apparent data-model difference at multi-decadal timescales may also be caused by imperfect pseudoproxy or real world data at lower frequencies.

We demonstrate that interpretations of past climate, such as regional differences, may be strongly influenced by natural constraints in the proxy data such as inherent noise or spatial coverage. However, it is hardly possible to *a priori* estimate the magnitude and relative influence of these factors on different diagnostics, such as volcanic response vs. inter-hemispheric difference. Our study shows that comparison of reconstructions from different regions or data sources and data-model comparisons requires PPE or other approaches to evaluate sources of error in data and methods.

## Methods

### Reconstruction methods

We generate real-world and pseudoproxy reconstructions of NH and SH temperature over the last 1000 years. To be able to assess the findings of ref.^11^, we use their proxy network and Principal Component Regression (PCR) reconstruction method for the SH. For the NH, ref.^11^ used the reconstruction ensemble of ref.^34^. Reproducing this reconstruction using PPE is onerous and difficult to interpret as it consists of an ensemble of 521 reconstructions based on nine different methods and proxy networks, many of which are not publicly available^34^. We therefore use the recent tree-ring width and density network of ref.^5^ for the NH and a Composite Plus Scale (CPS) reconstruction approach very similar to ref^5^. PCR and CPS usually yield very similar results for continental and hemispheric index reconstructions^5,11,32,33^. We generate 100-member ensemble reconstructions^11^ for all experiments. The uncertainty bands shown for the reconstructions in all Figures are derived based on proxy and parameter resampling^11^ and the use of an ensemble of noise pseudoproxies (see below). Other sources of uncertainties, such as the calibration error, would increase the uncertainty range. As the aim of this paper is to compare different PPE and real-world reconstructions, we argue that it is most illustrative to display uncertainties resulting from the generation of pseudoproxies rather than adding other sources of error, which are not discussed herein and would have a similar effect on all our experiments.

To test the robustness of the results we also apply two alternative reconstruction methods: Pairwise Comparison (PaiCO)^37^ and an implementation of Bayesian Hierarchical Models (BHM) developed by ref.^38^. For both methods, we use the code available from ref.^37^, which has been used earlier for comparison with traditional linear methods^2,33^. For details about these reconstruction techniques we refer to refs^37,38^. Results for these reconstruction methods are displayed in Supplementary Figs 33–43.

### Proxy networks

The SH proxy network consists of 111 records from seven different archives (tree-rings, corals, ice cores, historical documents, lake and marine sediments and speleothems). Note that only seven of these records cover the full Millennium, most of the other proxies are shorter than 400 years (Supplementary Fig. 1). We thus use a nested approach to create the 1000-year reconstructions. In the NH network, 23 of the 53 publicly available tree-ring records extend back to the year 1000. Results using the alternative NH proxy network recently published by ref.^36^ do not affect our conclusions and are shown in Supplementary Figs 7–9.

### Instrumental data and reconstruction targets

We use hemispheric means of the GISTEMP temperature grid^71^ averaged over the May-April and January-December windows for the SH and NH reconstructions, respectively. A discussion of the suitability of the SH average given the sparse station coverage over some periods and regions and a comparison with other gridded products is provided in the supplementary material of ref.^11^.

We use an annual mean and full hemispheric mean target for the NH to allow comparison of our results with ref.^11^. Note that the NH proxy network was designed and used by ref.^5^ to reconstruct extratropical land-only summer temperatures. Our results are therefore biased towards this season and target domain and reconstructions are less skillful than reported in ref.^5^. However, Our reconstruction target and the one used by ref.^5^ are highly correlated (r = 0.85), and our results and interpretations also hold if we use the same target domain and season as ref.^5^, or the more extensive multi-proxy network of ref.^36^ (Supplementary Figs 4–9 and 28).

### Calibration and verification

All SH reconstructions are calibrated over the 1911–1990 period. For the NH, we use 1911–1988 (the last year with no missing data in the proxy matrix). We use three different verification metrics: RE (reduction of error), r^2^ (explained variance) and RMSE (root mean squared error)^35^.

1 $$RE=1-\frac{{\sum }_{i=1}^{n}{(xins{t}_{i}-xreco{n}_{i})}^{2}}{{\sum }_{i=1}^{n}{(xins{t}_{i}-{x}_{calib})}^{2}}$$

2 $$RMSE=\sqrt{\frac{1}{n}{\sum }_{i=1}^{n}{(xins{t}_{i}-xreco{n}_{i})}^{2}}$$

*x*_*inst*_ denotes instrumental values and *x*_*recon*_ the reconstructed values for each year *i* of the verification period. *x*_*calib*_ is the calibration period mean of the instrumental data. The RE value assesses the ability of the reconstructions to capture interannual variations in the target. An RE of 1 represents a perfect reconstruction, while negative values indicate less predictive skill than just using the climatology from the calibration period (*x*_*calib*_). The RE skill values presented in Fig. 3 are based on sub-periods within the calibration interval that are withheld from calibration. These periods are randomly sampled for each reconstruction member, have a length of 20–45 years and are divided in 10-year blocks (allowing one block to be shorter, depending on the total amount of verification years sampled). Note that the skill values reported in Fig. 3 are relatively low because of the parameter sampling for each ensemble member. The skill for the best estimate of the reconstruction (ensemble median) is substantially higher than the average skill of the individual ensemble members^5,11,72^. We nevertheless display the skill metrics across the ensemble arguing that the spread of the values for each PPE is also informative and because the focus of this study is on relative comparison of different PPE rather than optimized reconstruction skill. Furthermore, we calculate RE, explained variance and RMSE for the reconstruction ensemble medians over the period 1881–1910, which is fully independent from the calibration period of all ensemble members, but suffers from substantial quality loss due to sparse station coverage, particularly in the SH^73^.

Results for these additional verification metrics are presented in the SM (Supplementary Figs 10, 11 and 56–83). We identify our “best match” reconstructions by comparing all validation metrics with the real-world reconstruction, selecting the experiments with the smallest average RMSE between real-world and pseudoproxy skill metrics (SM section S2, Table S2).

### Model data

We use temperature data from two different models, for which ensemble simulations for the past1000 experiment are available: HadCM3^30^ consisting of 3 runs extending back to 1400 and one run covering the full Millennium and CESM1-CAM5^31^, consisting of a ten member ensemble covering the last Millennium. The model data were processed to cover the same temporal windows as the instrumental reconstruction targets (May-April for the SH, calendar year means for the NH).

### Pseudoproxy generation

The results presented in Figs 1–6 are based on statistical pseudoproxies that are generated by adding a certain type and amount of noise to the model temperature output sampled at proxy locations^16,19^ (See eq. 3 below). This approach has been widely used in the past (see ref.^17^ for a review) and it allows us to test the influence of location of proxies and amount and type of noise on our results. An alternative approach of generating pseudoproxies by using proxy system models^24,26–29^ is presented in Supplementary Section S4, showing that our conclusions are robust to the choice of pseudoproxy type (Supplementary Figs 12–16). In the following sections, we provide the details of the generation of the statistical pseudoproxies, with more details provided in the SM (section S3).

We generate a range of different pseudoproxy sets for each model simulation. As a basis for the pseudoproxy generation, we use the model temperature grids subsampled at the locations of the proxy data. At the time steps where the real proxies have missing values, the corresponding model grid-cell time temperatures were also replaced with missing values to have realistic proxy coverage over time. The pseudoproxies *PP* are created by adding noise *n* to the model temperatures at the proxy location *T*_*l*_ at each time step *t* (ref.^19^):

3 $$PP(t)={T}_{l}(t)+n(t).$$

For simplicity and in accordance with earlier studies using statistical pseudoproxies^17^, we assume that *n* is unrelated to temperature. Note, however that this is a simplification, because for all paleoclimate archives, *n* is a complex combination of climatic and non-climatic factors, some of which may not be independent from temperature (e.g. water availability, CO_2_ and light availability for tree-rings^74–77^). Again, as we obtain similar results with PSM-based pseudoproxies, we argue that this simplification does not affect our conclusions. We also use relatively simple reconstruction techniques that model the predictors as consisting of a temperature signal plus unrelated noise, similar to Eq. 3. For each climate model simulation, nine different sets of pseudoproxies were generated using different noise levels (Table S1). For each noise level, a set of 100 pseudoproxies were generated with different noise realizations but the same methodology to generate a 100-member reconstruction ensemble.

First, no-noise pseudoproxies are used, i.e. directly using the model temperature time series from the locations of the proxy data *T*_*l*_ as predictors (*NoNoise* experiment).

Second, we created a set of Gaussian white-noise pseudoproxies as used in most PPE in the past^17^ using signal to noise ratios (SNR) of the fraction of standard deviation from temperature divided by that from noise of 1, 0.5 and 0.25, with the limit of a perfect proxy having infinite SNR. Thus, the variance of noise *n* is^17^:

4 $$var(n)=var({T}_{l})\ast \frac{1}{SN{R}^{2}}{\rm{.}}$$

Third, white-noise PPs are constructed with realistic noise levels based on the correlations *r* of the real proxies with local instrumental data *T*_*l*_. The relationship between SNR and *r* is given by

5 $$SNR=\sqrt{\frac{{r}^{2}}{1-{r}^{2}}}$$

For the SH, correlations from the proxy screening procedures of ref.^11^ are used (see their SM). For the NH, correlations were calculated between the real proxy data and the GISS gridded temperatures over the 1911–1990 period. As target value for the pseudoproxy generation, we use the maximum of all correlations over the domain as opposed to the local grid cell correlation (see refs^22,78^ for a discussion of the two approaches). Fourth, we use an AR1 noise model instead of white noise for the pseudoproxies:

6 $$n(t)=\rho \ast n(t-1)+{\epsilon }.$$

ρ is the AR1 autocorrelation coefficient of the noise. We create the noise such that the resulting pseudoproxy has the same AR1 coefficient *β* as the corresponding real proxy. To obtain this, ${\epsilon }$ is calculated as Gaussian white noise with mean zero and variance:

7 $$var({\epsilon })=var(n)\ast (1-{\rho }^{2})$$

ρ is related to *β* and the AR1 coefficient *α* of *T*_*l*_ by

8 $$\beta =\frac{\alpha \ast var({T}_{l})+\rho \ast var(n)}{var({T}_{l})+var(n)}.$$

Solved for ρ, this yields

9 $$\rho =(\beta -\alpha )\ast \frac{var({T}_{l})}{var(n)}+\beta =(\beta -\alpha )\ast \frac{{r}^{2}}{1-{r}^{2}}+\beta $$

Last, we created additional pseudoproxies with realistic correlations with the hemispheric field mean target *T*_*t*_ and correct proxy autocorrelations. Note that this approach does not imply that we claim a direct influence of hemispheric mean temperatures *T*_*t*_ on the observed proxy variables such as tree growth. Instead, it considers the proxy to be a combination of the target of reconstruction (hemispheric mean) and in part local effects away from the hemispheric mean in addition to proxy noise (see also ref.^20^). As stated above, the PCR and CPS reconstruction methods used herein effectively model the predictor time series to consist of a signal (in this case *T*_*t*_) plus noise. Thus, for the purpose of testing different approaches of constructing statistical pseudoproxies and motivated by the unrealistically low correlations of local model temperature with hemispheric means (see main text), we consider this a reasonable sensitivity test. These pseudoproxies are created using information from *T*_*t*_ and *T*_*l*_ as

10 $$PP(t)=\frac{({T}_{l}(t)+{T}_{t}(t))}{2}+n(t)$$

And the target correlation r is now given by

11 $$\begin{array}{rcl}{r}_{t} & = & cor(PP,{T}_{t})=\frac{cov(PP,{T}_{t})}{{\sigma }_{PP}\ast {\sigma }_{{T}_{t}}}\\  & = & \frac{cov(\frac{{T}_{l}}{2},{T}_{t})+cov(\frac{{T}_{t}}{2},{T}_{t})+cov(n,{T}_{t})}{\sqrt{var(\frac{{T}_{l}+{T}_{t}}{2})+var(n)+2\ast cov(\frac{{T}_{l}+{T}_{t}}{2},n)}\ast \sqrt{var({T}_{t})}}\end{array}$$

Assuming *n* is uncorrelated to any temperature data this yields

12 $$var(n)=\frac{{(cov({T}_{l},{T}_{t})+var({T}_{t}))}^{2}}{4\ast {r}_{t}^{2}\ast var({T}_{t})}-\frac{var({T}_{l}+{T}_{t})}{4}.$$

*ρ* is now obtained by

13 $$\rho =(\beta -\gamma )\ast \frac{var({T}_{l}+{T}_{t})}{4\ast var(n)}+\beta $$

where *γ* is the AR1 coefficient of ${T}_{l}+{T}_{t}$.

Note that in practice, there can be a trade-off between obtaining realistic target correlations *r* and realistic AR1 coefficients *β*. For example, if the target correlation is high (i.e. the variance of the noise needs to be small), it is impossible to get pseudoproxies with low autocorrelation, because autocorrelation of local and field mean temperature is usually high in model data. To deal with this, we set 0 ≤ *ρ* ≤ 0.9 and repeat the pseudoproxy generation 100 times selecting the realization with the smallest product of the standardized differences from the target values for *β* and *r*. The median absolute differences between the target and obtained values are 0.015 for *β* and 0.090 for *r* in the *TargetCor* experiment.

The PPE used and described in the main text are experiments using AR1 pseudoproxies with SNR 1 based on local grid cell correlations (*LocalCor* experiment, #8 in Table S1) and AR1 pseudoproxies using individual SNR for each proxy based on its correlation with the field mean (*tTargetC cor* experiment, #7 in Table S1). These experiments were selected because they are closest to the real-world reconstructions in terms of reconstruction skill (Table S2). Plots for the different PPE and skill metrics are presented in the SM.

### Randomly sampled proxy locations

To test the influence of proxy locations on the PPE, we perform alternative reconstruction for the *NoNoise* experiment by randomly sampling pseudo proxy locations. We use the same number of records as in the other experiments (53 for the NH; 111 for the SH). Latitudes and longitudes (including land and ocean) from the model field are randomly sampled for each record (with replacement) within the target hemisphere. The sampling was repeated 1000 times to generate 1000 pseudoproxy matrices, which were each used to generate one single-member reconstruction using the original PCR (SH) and CPS (NH) reconstruction technique. The result illustrates to what extent the actual locations of proxies bias the reconstructions.

### Inter-hemispheric differences

Inter-hemispheric differences are generated as in ref.^11^: Reconstructions and model data are first detrended using a 200-year loess-filter, to evaluate the decadal to centennial coherence. Second, ten-year running averages are calculated and divided by the standard deviation over the full Millennium to allow relative comparison of hemispheric fluctuations. Finally, each of the 100 standardized filtered SH reconstruction ensemble members is subtracted from one of the 100 NH reconstruction ensemble members.

### Volcanic eruption superposed epoch analysis (SEA)

We select the largest volcanic eruptions of the last Millennium based on dataset from ref.^44^, selecting only the events with a forcing exceeding −7.5 w/m^2^, which yields 14 eruptions. For each of these events, time series of the temperature reconstructions between −5 and +9 years from the event were selected, detrended and converted to anomalies with respect to the years −5 to −1 from the eruption. The mean of these time series from all 14 eruptions is used as estimate of the volcanic response for each reconstruction member. Best estimates and confidence ranges in the plots are the median and 5^th^-95^th^ percentiles of the reconstruction ensemble, respectively. This response is compared to temperature anomalies during years not affected by volcanic eruptions (i.e. not within −5 to +9 years from an eruption). From all these years, 14 time slices of 15 years are selected and processed as described above for the volcanic events. This was repeated 100 times to generate the confidence interval of temperature “response” during non-volcanic years. For the model simulations and PPE, we used the volcanic forcing time series used in each simulation to select the events. These are the “Gao” forcing dataset^79^ for the CESM-CAM5 simulations and the “Crowley” forcing dataset^80^ for HadCM3. To have a comparable threshold for event selection, these forcing reconstructions were scaled to the forcing reconstruction from ref.^44^ based on the three large eruptions Samalas, Kuwae and Tambora. This yields 9 and 11 eruptions for the “Gao” and “Crowley” datasets, respectively. Note that these epoch results hence reflect noise and proxy locations, but not possible errors and uncertainties in the volcanic forcing itself.

### Detection and Attribution experiment

D&A is usually used to determine to what extent real proxy data show response to external forcing expected from model-based fingerprints and is used here to investigate to what extent noise and proxy location may bias detection results. The method used here is based on a total least squares regression^30,64^ and is the same as used by ref.^12^. To estimate the relative contribution from external forcings and internal chaotic variability in pseudoproxy reconstructions, the CESM and HadCM3 model ensemble means are used to determine “fingerprints” of forced change. A scaling factor β is estimated which best matches the time dependent fingerprint X(t) to the pseudoproxy reconstruction Y(t), taking into account uncertainty in the form of internal variability in the model fingerprint *v*(*t*) and pseudoproxy reconstruction *v*_0_(t).

14 $$Y(t)=(X(t)-v(t))\,\beta +{\nu }_{0}(t)$$

Uncertainty ranges for the scaling factor, *β* are determined by a Monte-Carlo technique, where random samples of internal variability from all available CMIP5 ‘piControl’ simulations of sufficient length are added to an estimate of the (noise-reduced) model fingerprints and reconstructions^30^. The scaling factors therefore estimate what amplitude of external forcing is consistent with the reconstruction (usually the real reconstruction, here the pseudo-reconstruction), given internal variability. If a scaling factor of zero can be significantly excluded it can be shown that the external forcing is detectable. If a scaling factor is small for the pseudoproxy reconstruction, this illustrates that the network and noise will make the fingerprint difficult to detect. Pseudoproxy results can be compared with D&A results using real reconstruction in order to determine if failure to detect a signal or detection of a significantly smaller signal could be due to proxy properties.

In many D&A analyses (see e.g. ref.^65^), a spatio-temporal fingerprint is used for the analysis. Here, because Y(t) is an annual mean temperature reconstruction of either the NH or SH, the fingerprints, X(t), are also annual hemispheric mean surface air temperatures and are calculated from the ensemble model mean, from the corresponding hemisphere. For the PPE experiments a pseudo-reconstruction calculated from a single model simulation from either the CESM or HadCM3, as described above, is used for Y(t), and an associated scaling range, *β*, is calculated using exactly the same method as for the real reconstructions. In these cases the model fingerprint X(t) is calculated as the mean from all remaining simulations.

### Data availability

The real-proxy data from the SH are available at: https://www.ncdc.noaa.gov/paleo-search/study/16196, the proxy records from the NH at: https://www.ncdc.noaa.gov/paleo-search/study/19743. Pseudoproxy data and reconstructions from this paper are available at the NOAA World Data Center for Paleoclimatology (www.ncdc.noaa.gov/paleo).

## Electronic supplementary material

Supplementary information
