# Supplementary material for: Possible causes of data model discrepancy in the temperature history of the last Millennium
Source: Sci Rep. 2018 May 15;8:7572. doi: 10.1038/s41598-018-25862-2 (PMC5953951; doi:10.1038/s41598-018-25862-2)
Supplement: Supplementary file 1 — Supplementary information [file 41598_2018_25862_MOESM1_ESM.pdf]

# SUPPLEMENTARY MATERIAL

## Possible causes of data model discrepancy in the temperature history of the last Millennium

**Raphael Neukom[1*], Andrew P. Schurer[2], Nathan. J. Steiger[3] and Gabriele C. Hegerl[2]**

[1]Oeschger Centre for Climate Change Research and Institute of Geography, University of Bern, Switzerland

[2]School of Geosciences, University of Edinburgh, Edinburgh, UK

[3]Lamont-Doherty Earth Observatory, Columbia University, Palisades, New York, USA

*Corresponding author (neukom@giub.unibe.ch)

## Content

1. Proxy maps and local and hemispheric mean correlations ........ 2
2. Alternative NH targets, proxy data and reconstructions ........ 4
   Sensitivity to NH target season and domain ........ 4
   Alternative NH proxy data and reconstructions ........ 5
3. Pseduoproxy generation ........ 9
4. Proxy-Sytem-Model – based pseudoproxies ........ 15
5. Reconstruction amplitude ........ 21
6. North – South correlations and differences ........ 25
7. Extreme decades ........ 27
8. Superposed Epoch Analysis for volcanic eruptions. ........ 28
9. Additional plots for the D&A scale factors ........ 30
10. Alternative reconstruction methods. ........ 32
11. Reconstruction time series for the individual model simulations ........ 42
12. Skill plots for the individual model simulations ........ 48
13. References ........ 76

## 1. Proxy maps and local and hemispheric mean correlations

In this section we provide maps of the SH and NH proxy locations and display the local and hemispheric mean correlations of the proxy data.

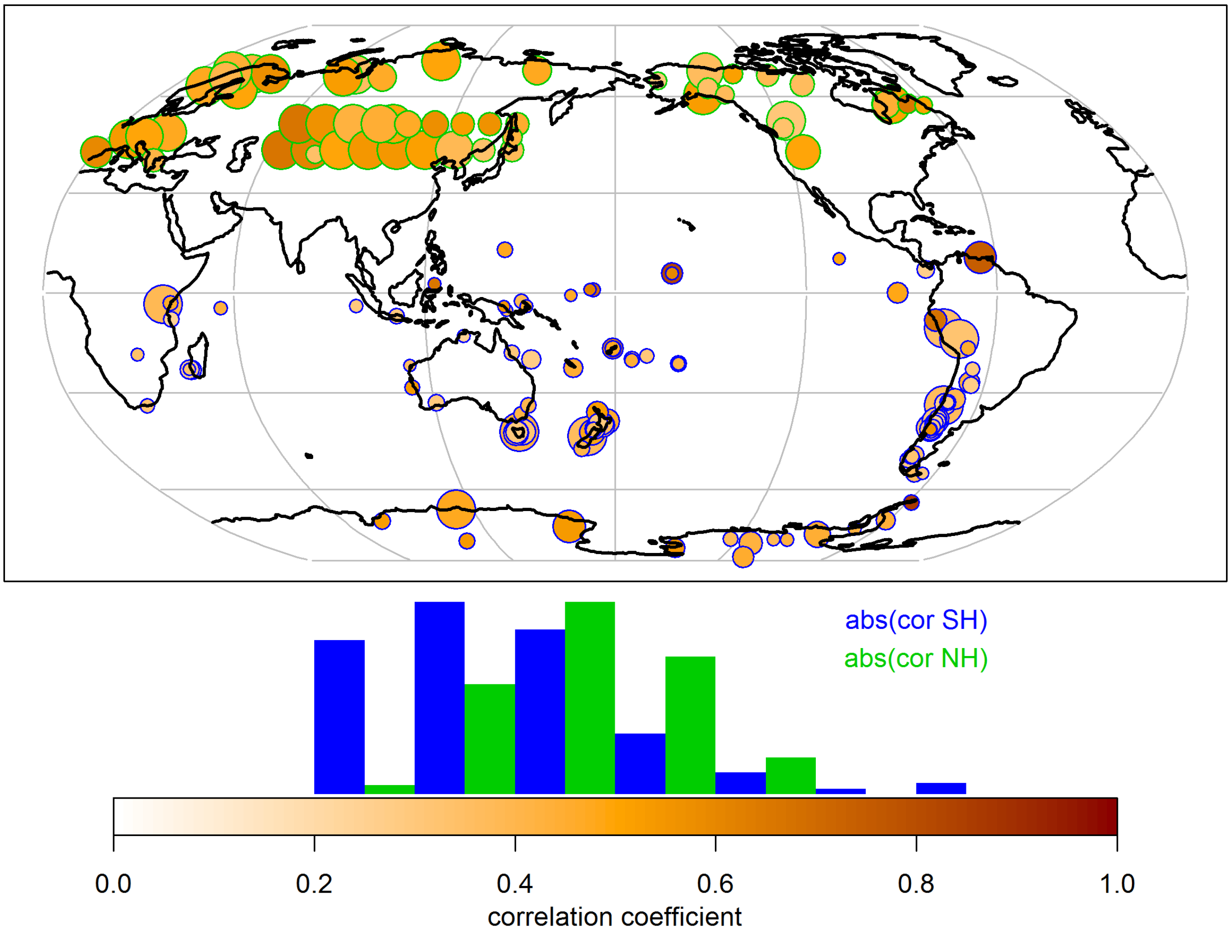


*Figure S1: Top: Location of proxy records used in this study. NH and SH proxies are taken from ref.[1] and ref.[2], respectively. The tropical records north of the equator are used for the SH reconstructions, because they have a stronger (and significant) relationship to SH mean temperature than to NH mean temperature[2]. Sizes of the circles represent the length of the record (between 111 and 1000 years) and the colors show the correlation with local temperature over 1911-1990 (details see Methods in the main text). Bottom: Green (blue) bars show the distribution of absolute correlations for the NH (SH) records. The map was generated using R (version 3.2.2, www.R-project.org).*

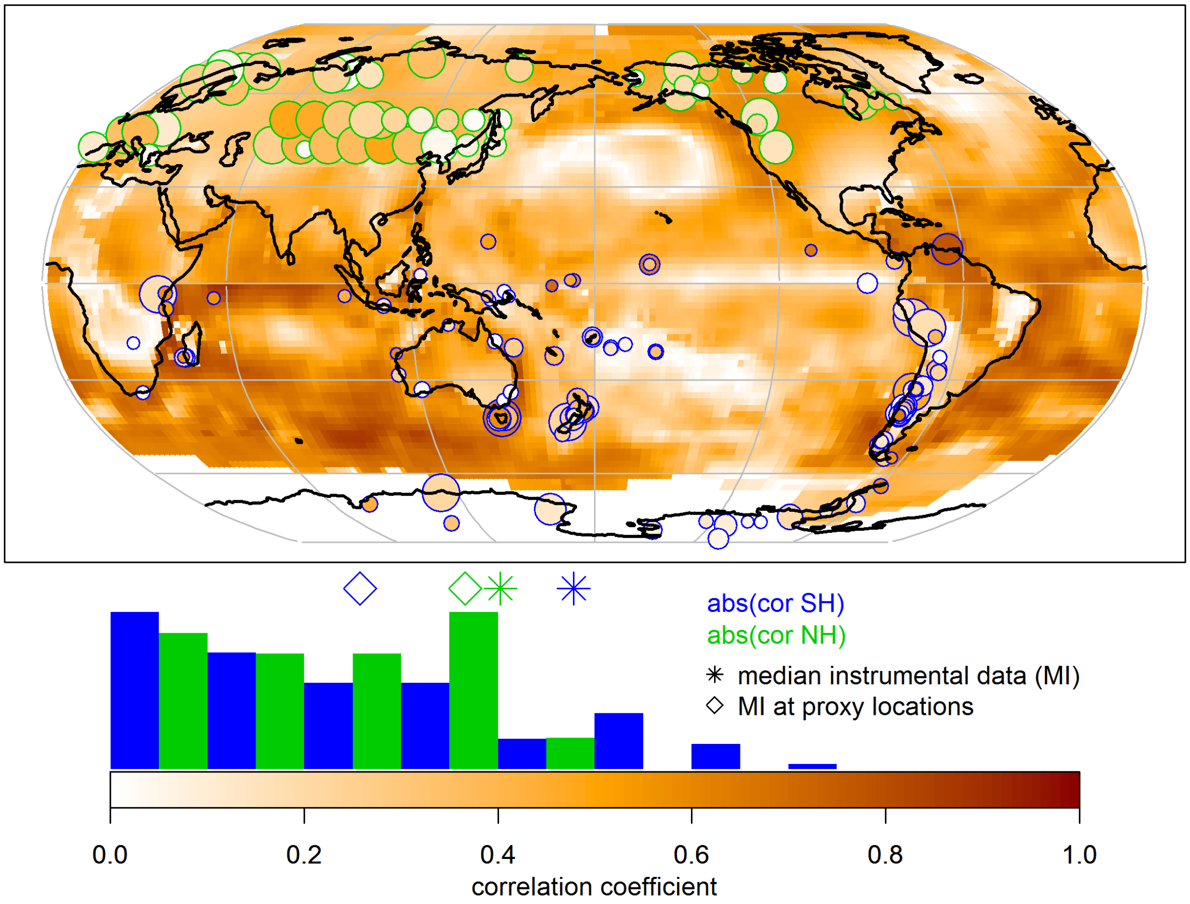


*Figure S2: Same as Figure S1 but showing correlations with the field mean target. Map shading represents correlation of grid-cell temperatures with the hemispheric mean in the GISTEMP target grid. Asterisks (diamonds) show the median correlations of instrumental grid-cell temperatures with the field mean for all locations (proxy locations only). The map was generated using R (version 3.2.2, www.R-project.org).*

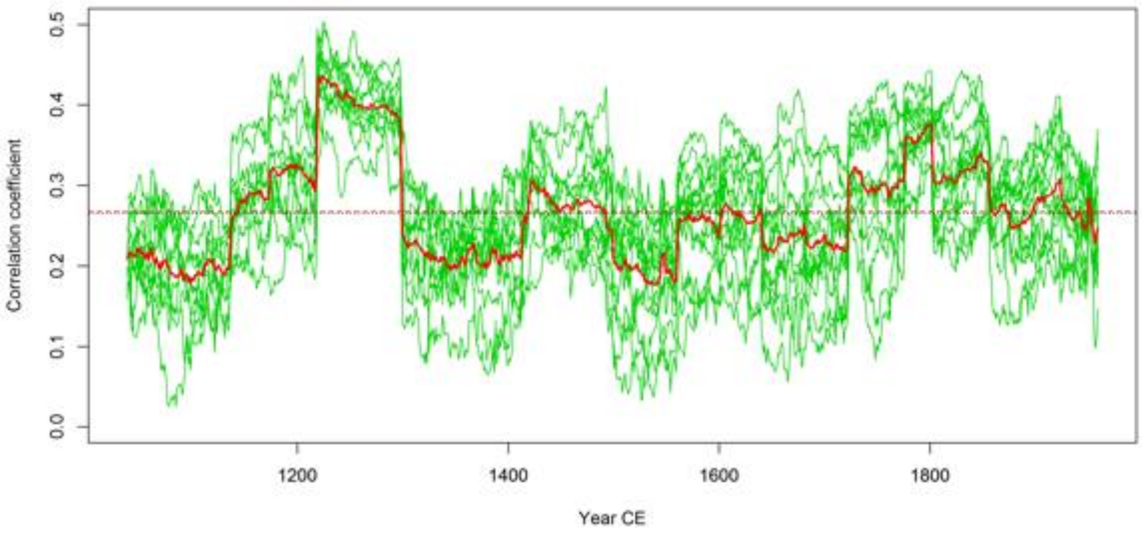


*Figure S3: 80-year running correlation of temperature at NH proxy locations with the NH average in the model data. Green lines: Median correlation of all proxy locations for each model simulation. Red: Mean of all model simulations. Black dotted horizontal line: Median value over the calibration period (1911-1990) as reported in the main text. Red dashed horizontal line: Mean of the red line over the last Millennium.*

## 2. Alternative NH targets, proxy data and reconstructions

### Sensitivity to NH target season and domain

We use a full hemispheric mean over the calendar year window as NH reconstruction target to allow comparing our results with ref.[2], who used the reconstruction ensemble of ref.[3] for the NH. Ref.[1] use a May-August (MJJJA) extra-tropical land-only target for their reconstruction. While this extra-tropical temperature composite has a much larger variance, the two targets are highly correlated (Figure S4) and so are the resulting reconstructions (Figure S5). Our results and conclusions are not sensitive to the choice of the NH reconstruction target (Figure S6 and Figure S28).

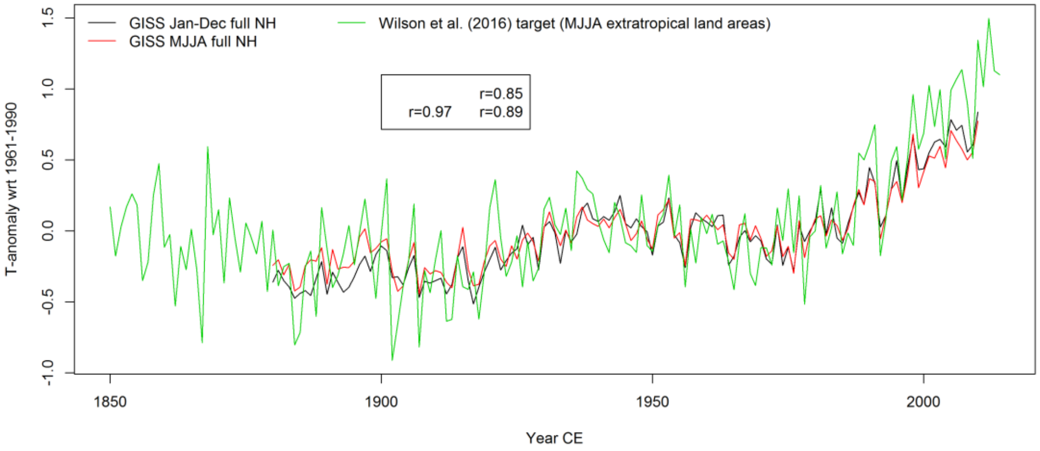


*Figure S4: Comparison of different NH temperature averages. Black: GISS calendar year full NH mean as used in this study as reconstruction target. Red: Same but using an MJJA seasonal window. Green: Reconstruction target used by ref.[1]. Correlations between the time series are indicated in the box.*

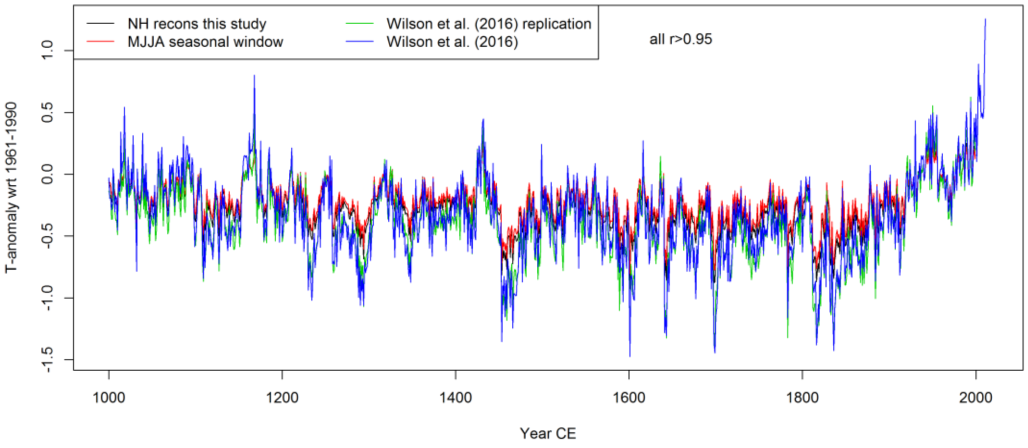


*Figure S5: Comparison of NH reconstructions based on different reconstructions targets. Black: NH reconstruction used herein with a calendar year full NH mean. Red: Same but using an MJJA target seasonal window. Green: Replication of the reconstruction of ref.[1] using their input target data but our CPS reconstruction methodology. Blue: Reconstruction of ref.[1].*

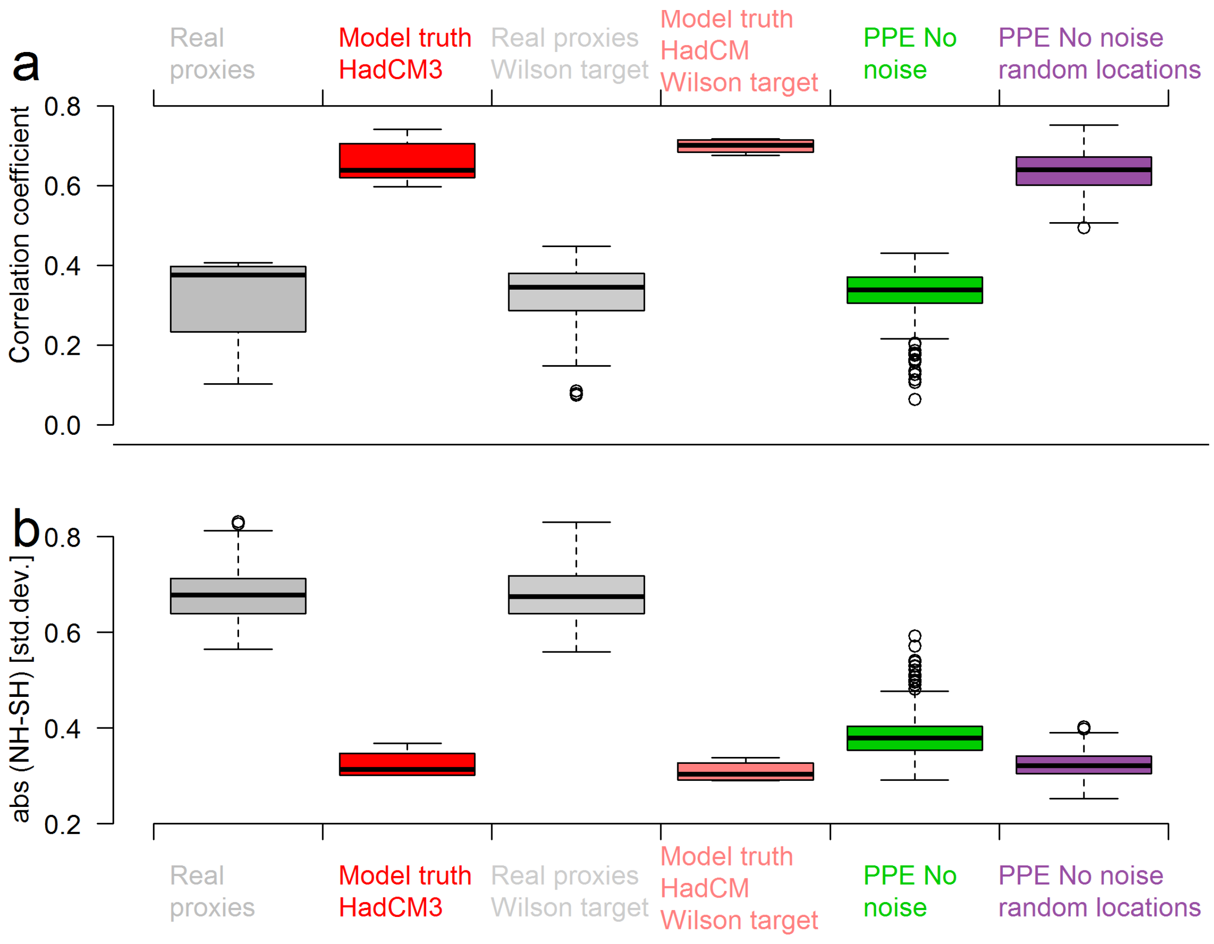


*Figure S6: Same as Figure 4 in the main text but comparing different NH mean target datasets for the HadCM3 simulations. The 3rd and 4th columns use an MJJA window and extra-tropical land-only target (target time series used in Wilson et al. (2016)). The other columns are based on the Jan-Dec window and full hemispheric mean target used in all Figures in the main text.*

## Alternative NH proxy data and reconstructions

Figure S7-Figure S9 compare the real-world NH reconstruction based on the proxy network of ref.[1] with alternative reconstructions: A reconstruction based on the same CPS methodology but using the new NH proxy collection of ref.[4] and the reconstruction ensemble of ref.[3].

The NH reconstruction used herein apparently has a relatively small Present-day – LIA amplitude compared to the other reconstructions (Figure S8). Note that this is mainly because this reconstruction has relatively warm values during 1600-1650, which was used as LIA baseline period. Using an alternative baseline period would yield much more similar amplitudes between the three reconstructions (not shown).

Despite these differences, our results and conclusions are not affected by the choice of the NH reconstruction as shown by Figure S9. The NH reconstruction based on the dataset of ref.[4] shows slightly better agreement with the model data (e.g. higher NH vs. SH correlations). Note that this database has a higher number of records that are more homogeneously distributed across the NH, confirming our interpretation of the importance of well distributed proxy locations.

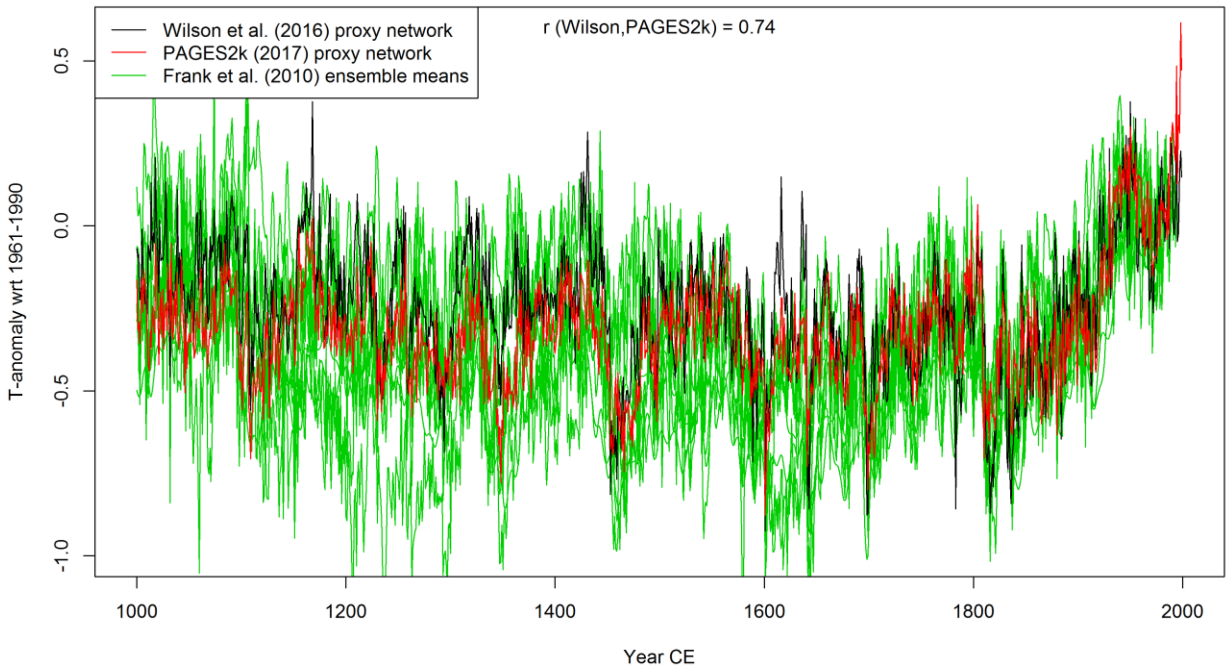


*Figure S7: Comparisons of different NH real proxy reconstructions. Black: Proxy network of ref.*[1] *as used in the main text. Red: NH reconstruction using the NH proxy data from ref.*[4] *and the same CPS method as for the black line. Green: Ensemble medians of the nine individual NH reconstructions that went into the NH ensemble of ref.*[3]*. Correlation between the black and red lines is indicated at the top.*

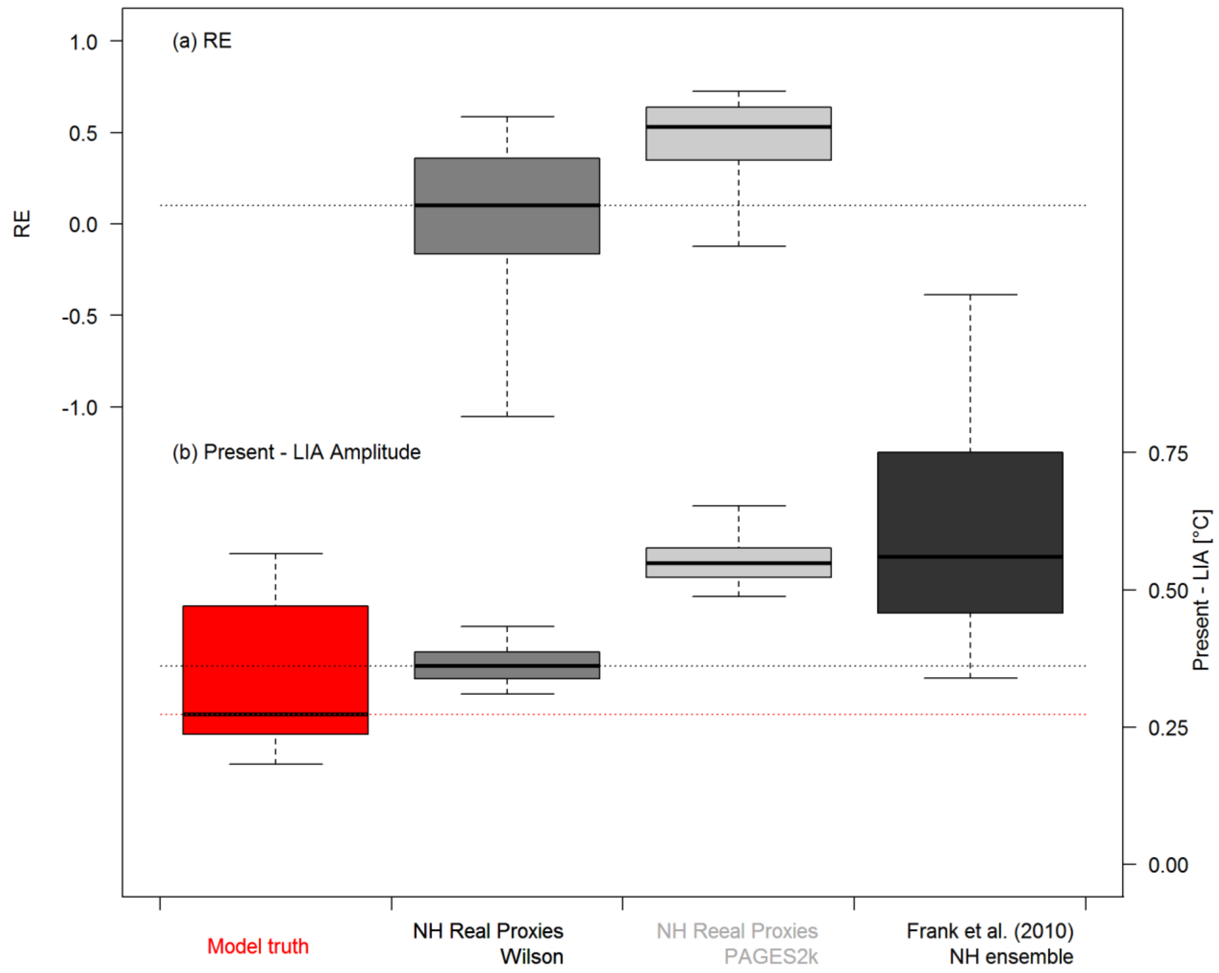


*Figure S8: Same as Figure 1 in the main text but for the different NH real proxy reconstructions.*

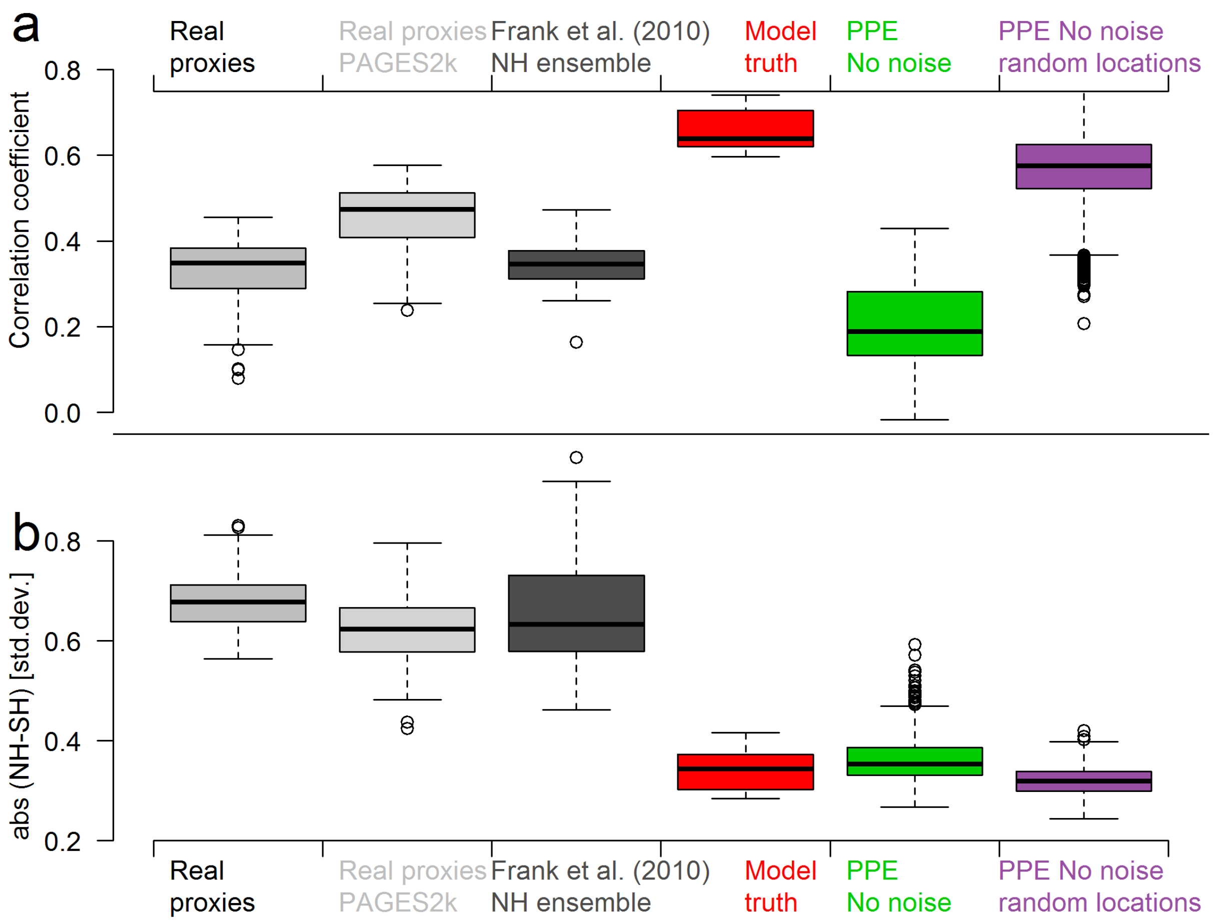


*Figure S9: Same as Figure 4 in the main text but including the different NH real proxy reconstructions.*

## 3. Pseduoproxy generation

In this section, we provide additional information and a more detailed description of the pseudoproxy generation described in the Methods section of the main text.

An overview of the different pseudoproxy generation methods applied herein is presented in Table S1. We generated a range of PPE using different concepts and amounts of noise. First, the model field is subsampled at the locations of the proxy records in the SH[2] (Figure S1) and NH[1]. At the time steps where the real proxies have missing values, the corresponding model grid-cell time temperatures were also replaced with missing values to have realistic proxy coverage over time. These grid-cell time series from the model simulations are then subjected to the reconstruction methods to estimate hemispheric mean temperatures (Perfect pseudoproxies, *NoNoise* experiment #1). In the other experiments #2-#9 white or AR1 noise is added to the sub-sampled model grid cell time series in order to mimic the behavior of real proxy data. Typically, white noise with signal to noise ratios (SNR, defined here as standard deviation ratio of the subsampled model time series and the added noise) of 1, 0.5 and 0.25 (experiments #2-#4) are used in the literature (ref.[5] and references therein). In addition, we also generate pseudoproxies with realistic noise levels for each proxy. This means that the SNR is calculated for each proxy individually based on its correlation with local temperatures in the real world (equation 5 in the Methods). This is in contrast to earlier studies[6,7], where SNR was sampled from a distribution generated based on the correlations of all proxies in the global proxy database. We use the individual empirical correlation for each proxy because the time period covered by the proxies in our database varies considerably. Hence, for the quality of the reconstruction at the beginning of the reconstruction period, it plays an important role whether the longest proxies have a high or low SNR. For realistic results, individual SNRs are therefore preferable. However, the realistic SNR values used herein (median 0.41 in the SH and 0.51 in the NH) are close to the ones reported by ref.[6], who found a mean SNR of 0.45 using a similar method.

In the real world, the noise on proxy data is probably not white, as non-proxy noise can arise from temporally and even spatially correlated causes. Therefore it may be important that the pseudoproxies have similar spectral properties as the real proxies. We therefore created pseudoproxies that have the same AR1 coefficient as the corresponding real proxies[8,9]. We generated two sets of such AR1 proxies, one with realistic temperature correlations (SNRr-AR1 experiment #6) and one with SNR 1 (SNR1-AR1, #8)

As described in the main text and methods, we generate additional pseudoproxies, where we set the signal to noise ratio in a way that replicates the correlations of the proxy with the field mean target instead of local temperatures. This is done again by creating pseudoproxies with realistic AR1 properties using realistic SNR (SNRrt-AR1 experiment #7) and SNR 1 (SNR1t-AR1 experiment #9).

As expected, the PPE experiments based on target correlations show improved results in terms of skill and amplitude, compared to the pseudoproxies based on local correlations, particularly for the SH (Figure S10 and Figure S11). Experiment #9, using target correlations and SNR1 yields skill values that are clearly higher than those from all other experiments including the *NoNoise* experiment and real proxy reconstructions. From these results we conclude that an SNR of 1 is unrealistically high when using target instead of local temperature correlation (experiment #9). In contrast, the underperformance (i.e. lower skill) of #5 and #6 (realistic local correlations) compared to the real proxies (Figure S10 and Figure S11) is at least partly caused by the reduced correlation of local temperatures with the field mean target in the model world.

In the main text, we limit our interpretations to the experiments #1 (*NoNoise*), #8 (SNR1-AR1, label *LocalCor* in the main text) and #7 (SNRrt-AR1 label *TargetCor*), as these are most consistent with the real-world reconstructions in terms of reconstructions skill. To evaluate this consistency, we use five different verification skill metrics (formulae see Methods section of the main text): RE (Figure 3) and $r^2$ for each ensemble member over the verification period sampled individually for each member; and RE, $r^2$ and RMSE of the ensemble median over the 1881-1910 early verification period. We calculate the mean difference between real proxy reconstructions and PPE for each skill metric across all model simulations used

(temporally averaged across the reconstruction period, see also Figure S10 and Figure S11). The PPE are then ranked using the average value across all five skill metrics (after reversing the sign for RMSE, Table S2).

Of the noise-perturbed PPE, the SRN1 and SNR1-AR1 experiment are closest to the real proxies in both hemispheres. These two experiments have similar skill metrics and reconstruction time series (not shown), but the AR1-based PPE have more realistic spectral properties leading to performance that is more similar to the real-world data for some metrics (e.g. Figure S26). We therefore select SNR1-AR1 as "best match" experiment. From the two experiments based on target correlations, the SNRrt-AR1 PPE is clearly favorable and is used as second "best match" experiment shown in the main text. Sensitivity plots for the Figures in the main text based on all experiments are provided in Figure S26.

Skill plots for each individual model simulation are shown below (Section S12).

Table S1: Overview of Pseudoproxy reconstruction experiments. AR1 noise is generated such that the resulting pseudoproxies have the same AR1 coefficient as the corresponding real proxies. Realistic SNR means that the correlation of the pseudoproxy with the local or target temperature time series is the same as for the real proxies. Correlation indicates whether the SNR is calculated based on the correlation of the pseudoproxy with local or target (i.e. hemispheric mean) temperatures. Bolded experiments are the ones shown and discussed in the main text.

| # | Name | Noise type | SNR | Correlation |
|---|---|---|---|---|
| **1** | ***NoNoise*** | **none** | **∞** | **-** |
| 2 | SNR1 | white | 1 | Local |
| 3 | SNR0.5 | white | 0.5 | Local |
| 4 | SNR0.25 | white | 0.25 | Local |
| 5 | SNRr | white | Realistic | Local |
| 6 | SNRr-AR1 | AR1 | Realistic | Local |
| **7** | **SNRrt-AR1 (*TargetCor*)** | **AR1** | **Realistic** | **Target** |
| **8** | **SNR1-AR1 (*LocalCor*)** | **AR1** | **1** | **Local** |
| 9 | SNR1t-AR1 | AR1 | 1 | Target |

Table S2: Rank of the PPE in terms of realistic performance. Numbers in brackets indicate the mean difference in reconstruction skill between the PPE and the real-world proxies, averaged across five skill measures.

| Rank | SH | NH | SH+NH |
|---|---|---|---|
| 1 | SNR1 (0.13) | ***NoNoise* (0.14)** | SNR1 (0.28) |
| 2 | **SNR1-AR1 (0.15)** | **SNR1-AR1 (0.15)** | **SNR1-AR1 (0.31)** |
| 3 | SNRr-AR1 (0.15) | SNR1 (0.16) | ***NoNoise* (0.35)** |
| 4 | SNR0.5 (0.19) | **SNRrt-AR1 (0.16)** | **SNRrt-AR1 (0.35)** |
| 5 | SNRr (0.19) | SNRr (0.21) | SNRr-AR1 (0.36) |
| 6 | **SNRrt-AR1 (0.2)** | SNRr-AR1 (0.21) | SNRr (0.4) |
| 7 | ***NoNoise* (0.21)** | SNR0.5 (0.21) | SNR0.5 (0.4) |
| 8 | SNR0.25 (0.28) | SNR0.25 (0.29) | SNR0.25 (0.57) |
| 9 | SNR1t-AR1 (0.38) | SNR1t-AR1 (0.37) | SNR1t-AR1 (0.75) |

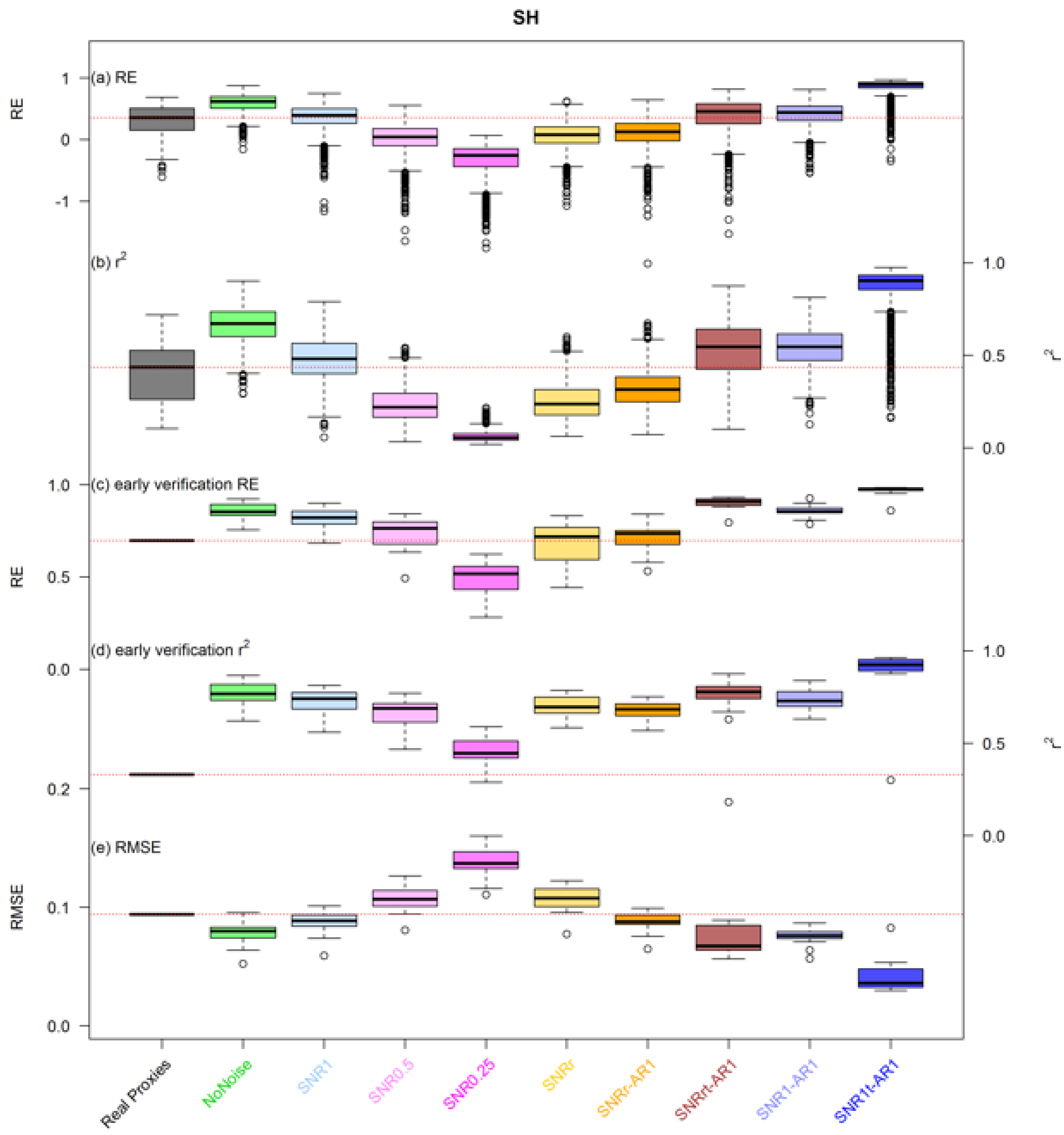


*Figure S10: Skill metrics for the SH real-world and PPE reconstructions. The data shown in Figure 3a in the main text are from the Real proxy, NoNoise, SNR1-AR1 (LocalCor) and SNRt-AR1 (TargetCor) experiments in panel (a).*

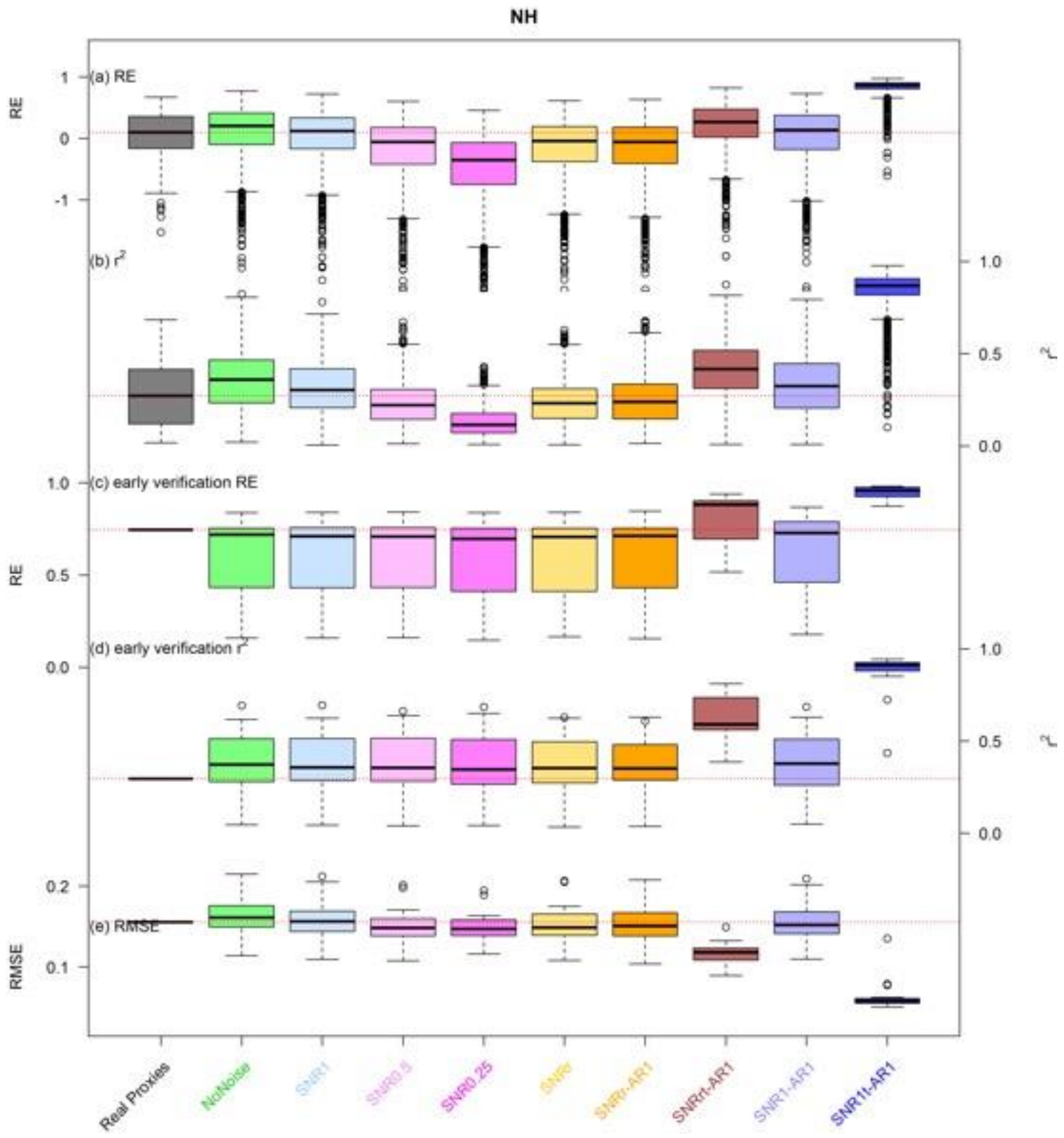


*Figure S11: Same as Figure S10 but for the NH.*

## 4. Proxy-Sytem-Model – based pseudoproxies

As an additional validation to assess the robustness of our results to pseudoproxy generation, we calculate a set of additional PPE based on pseudoproxies derived using proxy system models (PSM).

We use tree-ring width and coral $\delta^{18}O$ PSM pseudoproxies (PSM-PP) as described in Ref.[10]. Similar to Ref.[10], PSM pseudoproxies are computed for tree-ring width using the VS-lite model[11] and the model of Ref.[12] for coral $\delta^{18}O$. The PSM pseudoproxies are generated for the CESM1-CAM5 model ensemble, using the input variables of monthly temperature and precipitation for the trees and annual sea surface temperature and sea surface salinity for the corals. The proxy network is based on that of the updated PAGES2k proxy network[4] and realistic PSM parameters are derived from the PAGES2k proxy data and historical observational data, as in Ref.[10]. For the PSM-based PPE, we use 397 tree-ring and 44 coral PSM-PP with latitude > 0°N to reconstruct NH mean climate (as opposed to the tree-ring network of Ref. [1] in the main text, see also Figures Figure S9-Figure S11). For the SH, we generate additional PSM-PP for those tree-ring records that were used as SH temperature predictors in the reconstruction of Ref.[2], but are not included in the PAGES2k database (31 records). In the SH reconstruction of Ref. [2], only three out of the eleven records extending back beyond 1400 are coral or tree-ring data. The other records are Lake sediments (3), marine sediments (1) or ice cores (4). To allow for a skillful reconstruction in the first few centuries of the reconstruction period, these data are required. The model simulations we use herein are not isotope-enabled and there are, to our knowledge, no generally applicable PSMs for sediment archives. Therefore, we use the same noise-based pseudoproxy data as in the main text (*LocalCor* experiment) for the 32 non-tree-ring and -coral records in the SH, leading to a total of 124 records. Across both hemispheres, this results in a total of 565 records used for this experiment, 94% of which are PSM-based. We use the same target temperature datasets and seasonal windows as for the other PPE.

The PSM-PP have a median SNR of 0.52 [5$^{th}$ percentile: 0.05, 95$^{th}$ percentile: 3.17], thus slightly higher than those in our noise-based PPE with realistic local proxy correlations. The correlations with our reconstruction targets are 0.22 [0.03, 0.52] for the NH and 0.31 [0.03, 0.60] for the SH, thus also a bit higher than the real proxies (NH: 0.20 [0.03, 0.42]; SH: 0.20 [0.02, 0.58]).

Figure S12-Figure S15 show the same illustrations as in the main text but including the PSM PPE (cyan color). These figures show only results of the CESM model, but comparison with the figures in the main text shows that differences to the combined HadCM3 plus CESM results are marginal. The results of the PSM PPE are very similar to those of the *LocalCor* and *TargetCor* experiments for inter-hemispheric correlations, inter-hemispheric differences and the response to forcing indicating that our conclusions are robust to the choice of the method for pseudoproxy generation.

Interestingly, the PSM PPE show a larger loss of low-frequency variance (Figure S13b). This reduced present-day LIA amplitude is already evident in the PSM input proxy data (not shown). We hypothesize that it is caused by the fact that precipitation (tree-ring PSM) and sea surface salinity (coral PSM) are used besides local temperature to generate the PSM and these variables do not show the pronounced industrial-era warming trend that is inherent in temperature. Sea surface salinity incorporated in the coral PSM pseudoproxies (in addition to sea surface temperature) generally even trends in the opposite direction from temperature.

In contrast, the PSM PPE can reproduce the volcanic cooling and forcing response in D&A of the CESM model truth in the NH very well and much better than the *LocalCor* and *TargetCor* and *NoNoise* experiments (Figures Figure S15Figure S16). This is most likely caused by the fact that the PSM PPE is

based on eight times more input proxy data (441 as opposed to 53) in the NH, which are also distributed over the entire hemisphere (except regions north of 72°N) in contrast to the mid-latitudes-only network used for the other PPE. This corroborates our conclusion that proxy distribution is key to capture the response to external forcing.

Along with the alternative reconstruction methods (SectionS10) and targets (section S3) and additional noise-based PPE (Section S2), this PSM PPE experiment using a different concept of pseudoproxy construction and a larger proxy network supports the conclusions drawn in the main text.

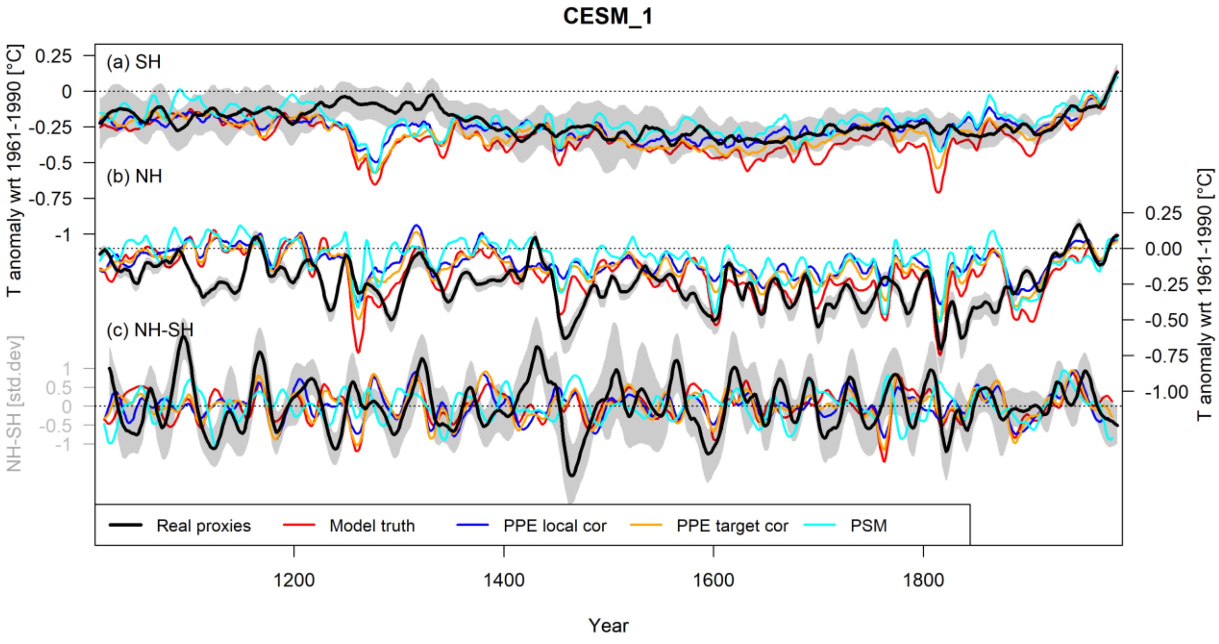


*Figure S12: Same as Figure 1 in the main text but including the PSM PPE (cyan).*

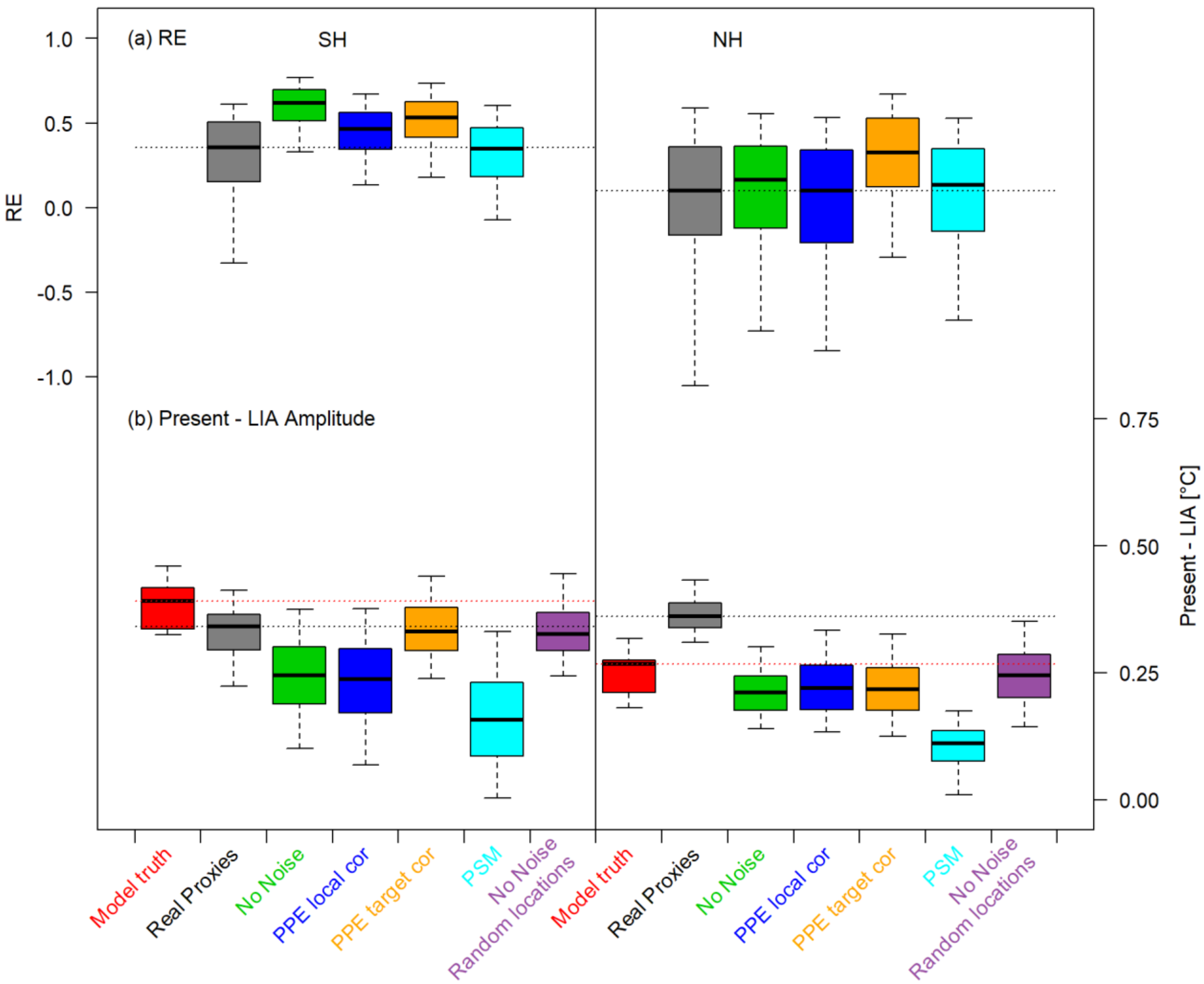


*Figure S13: Same as Figure 3 in the main text but including the PSM PPE (cyan) and only the 10 CESM model ensemble members.*

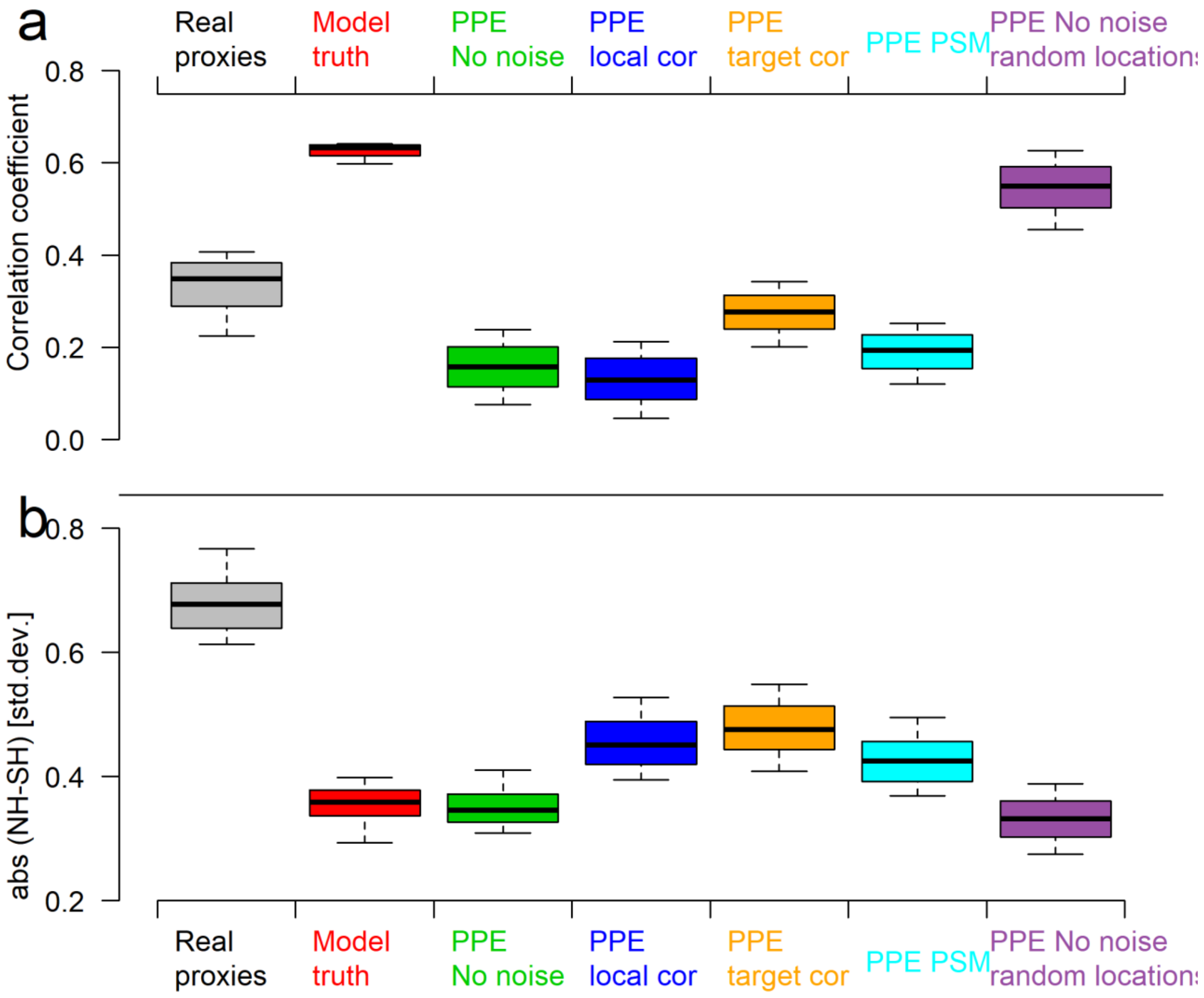


*Figure S14: Same as Figure 4 in the main text but including the PPE PSM (cyan) and only the 10 CESM model ensemble members.*

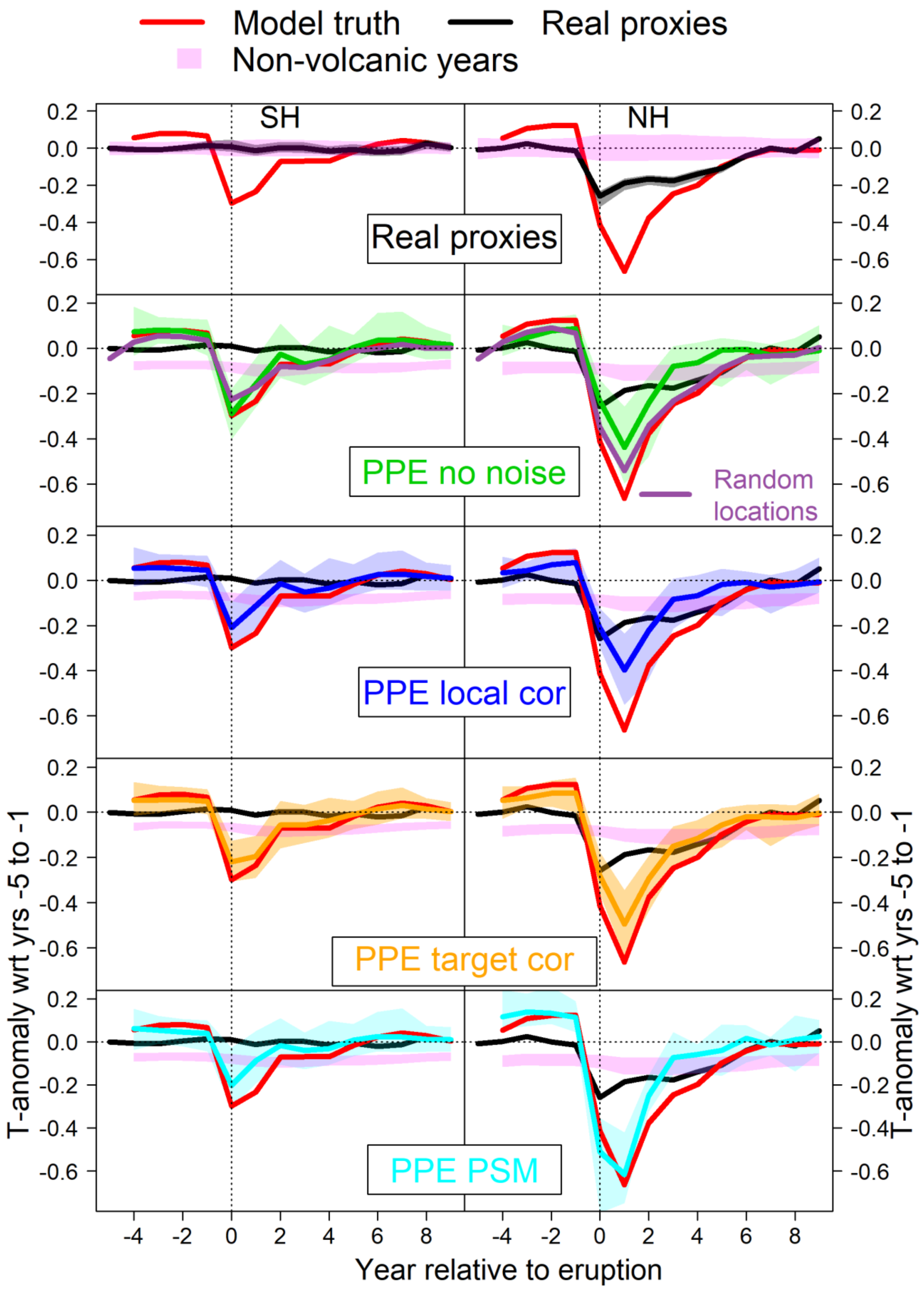


*Figure S15: Same as Figure 5 in the main text but including the PPE PSM (cyan, bottom panel) and only the 10 CESM model ensemble members.*

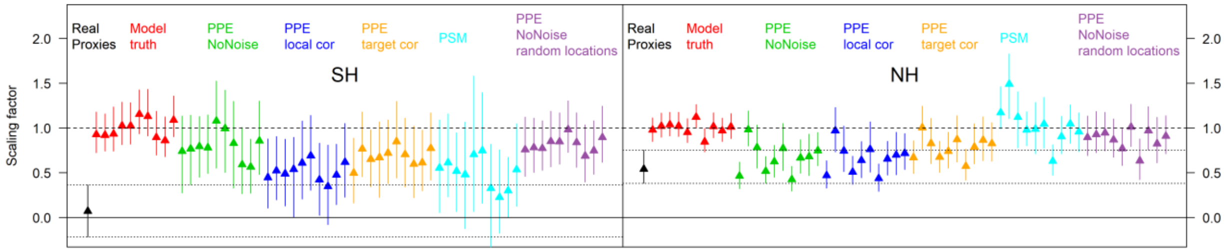


*Figure S16: Same as Figure 6 in the main text but including the PPE PSM (cyan) and only the 10 CESM model ensemble members.*

## 5. Reconstruction amplitude

In this section, we show additional plots for the present-day minus LIA amplitude (Figure 3 in the main text). We show the amplitudes for each experiment listed in Table S1 across all model simulations and the results for each model ensemble (HadCM3 and CESM1-CAM5) separately. The effect of the number of proxies available in PPE with randomly sampled locations is also shown in Figure S23 and Figure S24.

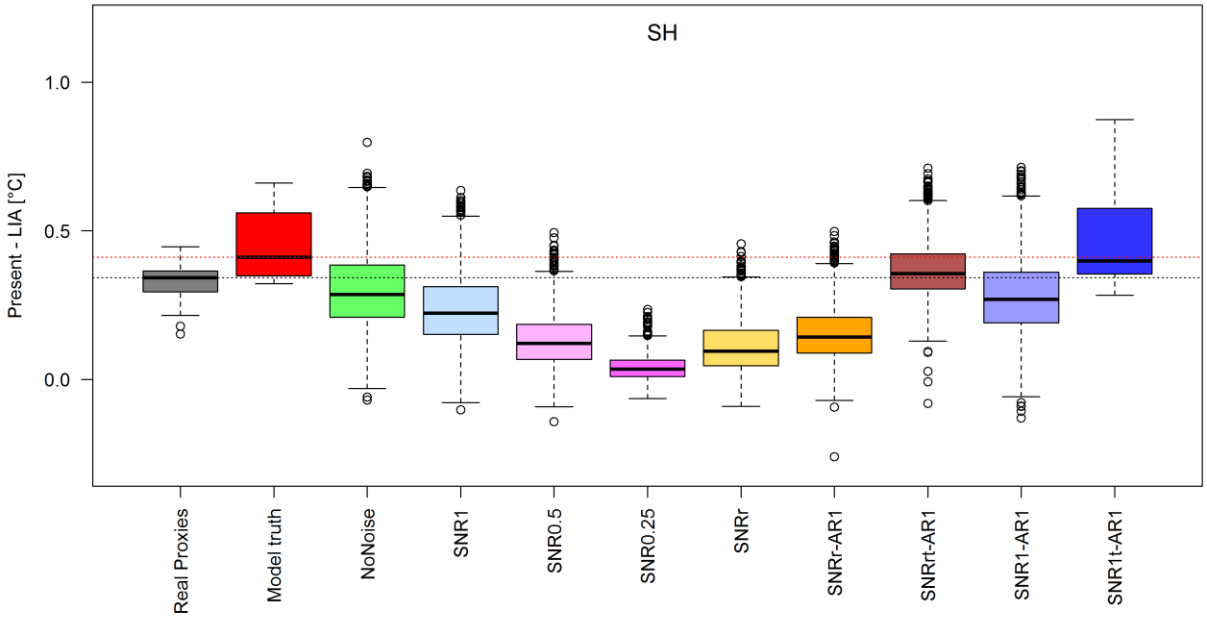


*Figure S17: Low frequency amplitudes of the SH reconstructions as in Figure 3b but for all PPE listed in Table S1.*

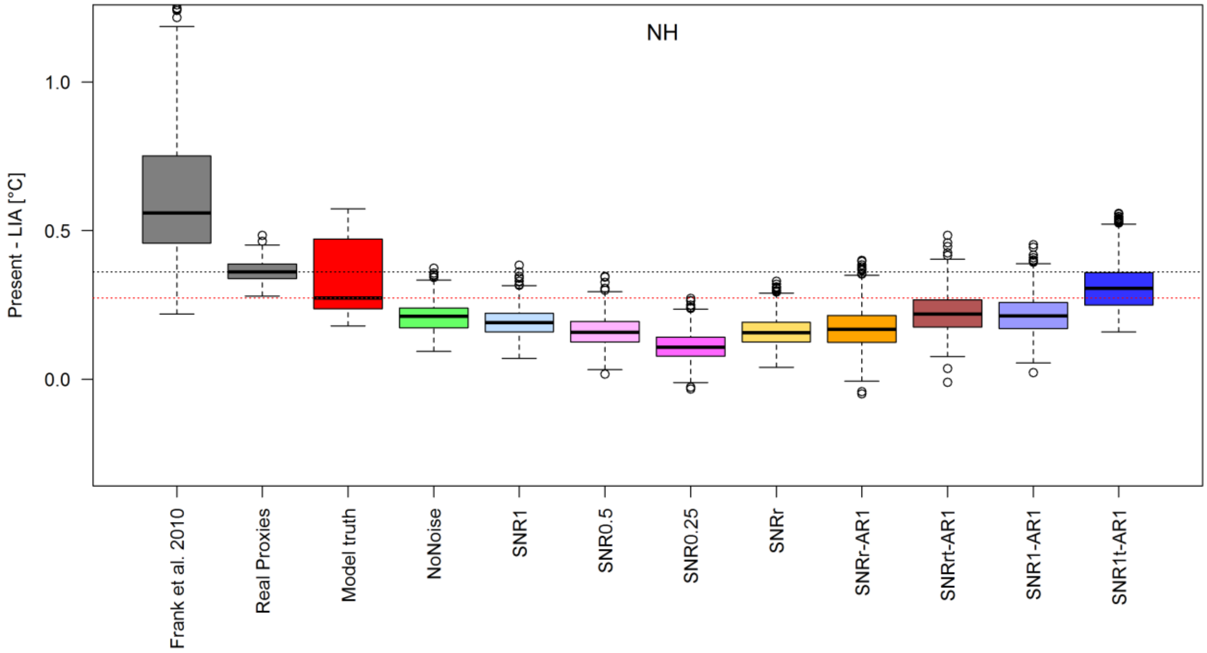


*Figure S18: Same as Figure S17 but for NH. Data from the NH real proxy reconstruction of Frank et al. [3] are shown in the first box on the left.*

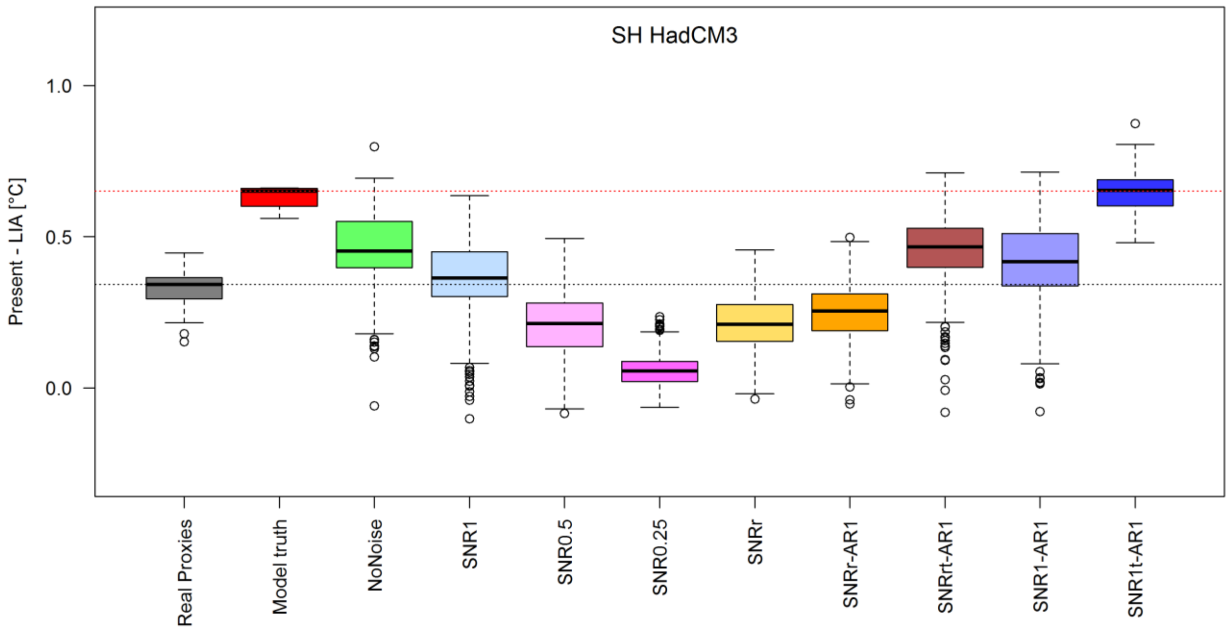


*Figure S19: Same as Figure S17 but only for the four simulations of the HadCM3 model.*

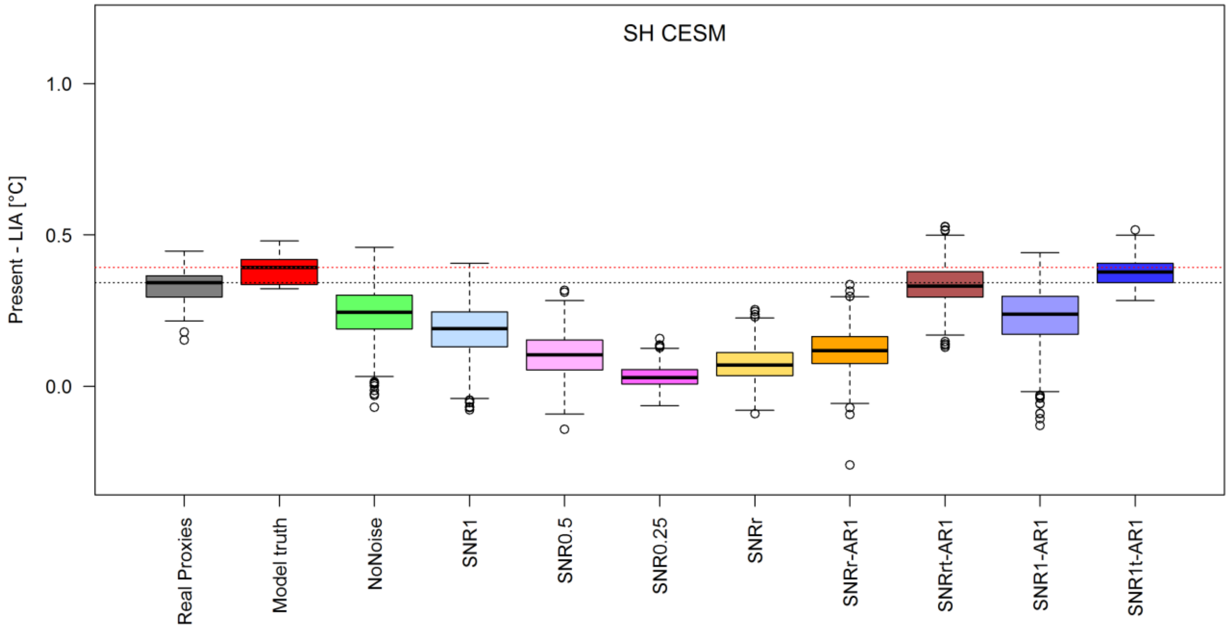


*Figure S20: Same as Figure S17 but only for the ten simulations of the CESM model.*

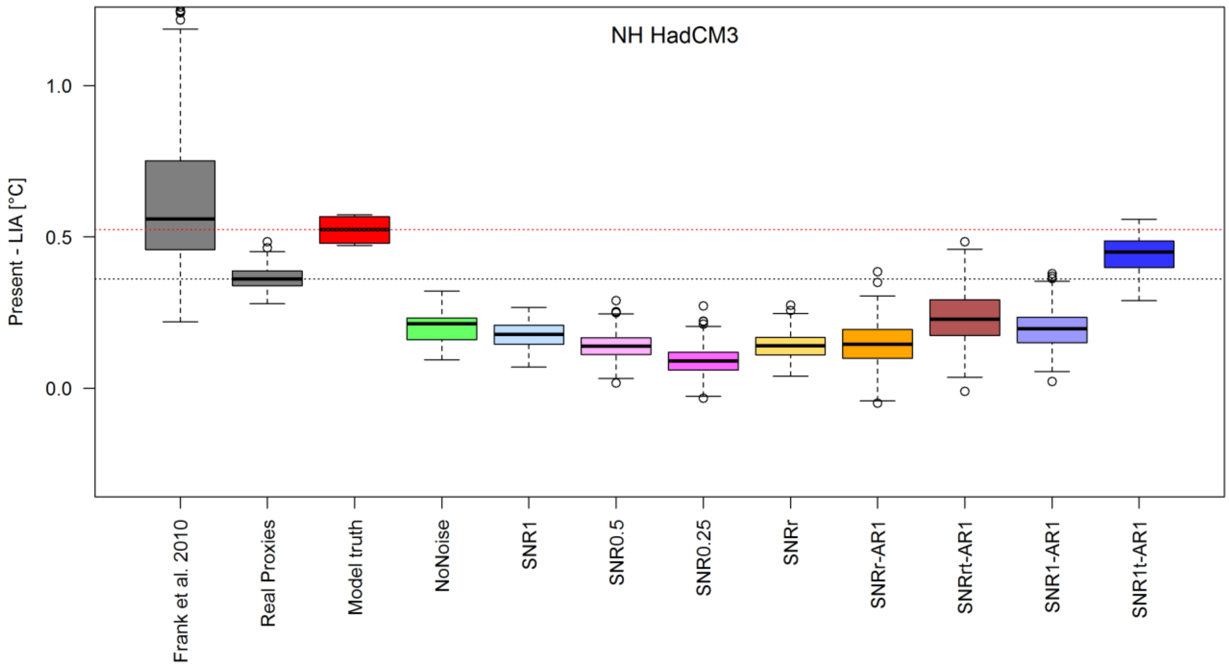


*Figure S21: Same as Figure S18 but only for the four simulations of the HadCM3 model.*

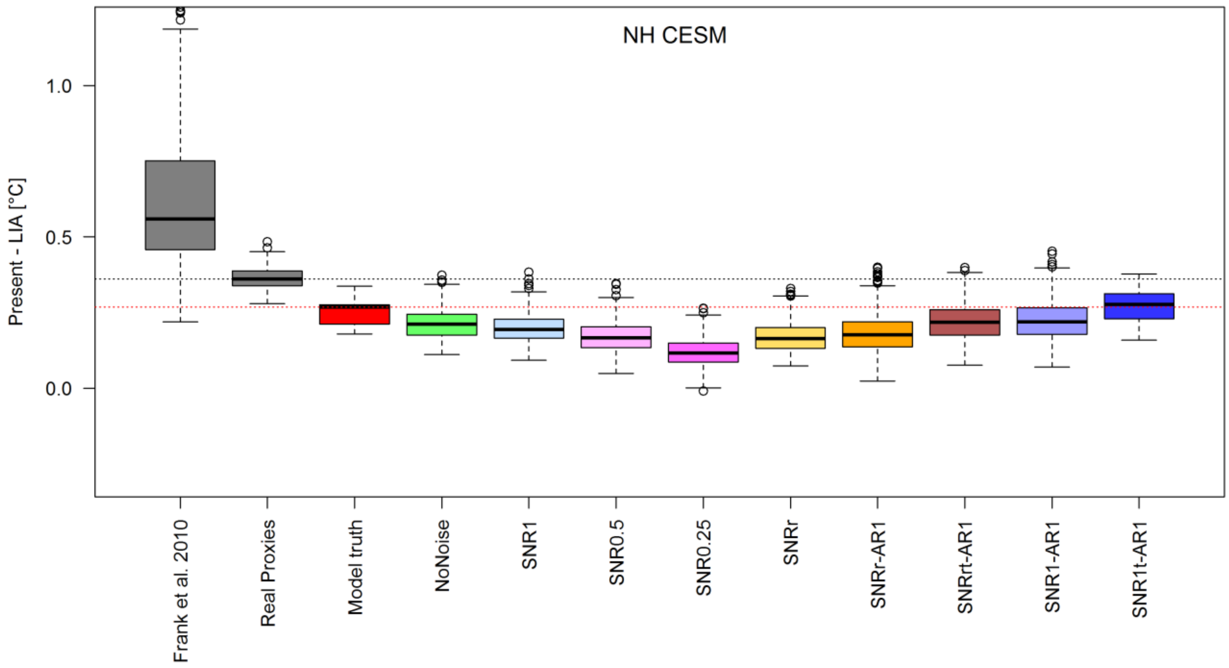


*Figure S22: Same as Figure S18 but only for the ten simulations of the CESM model.*

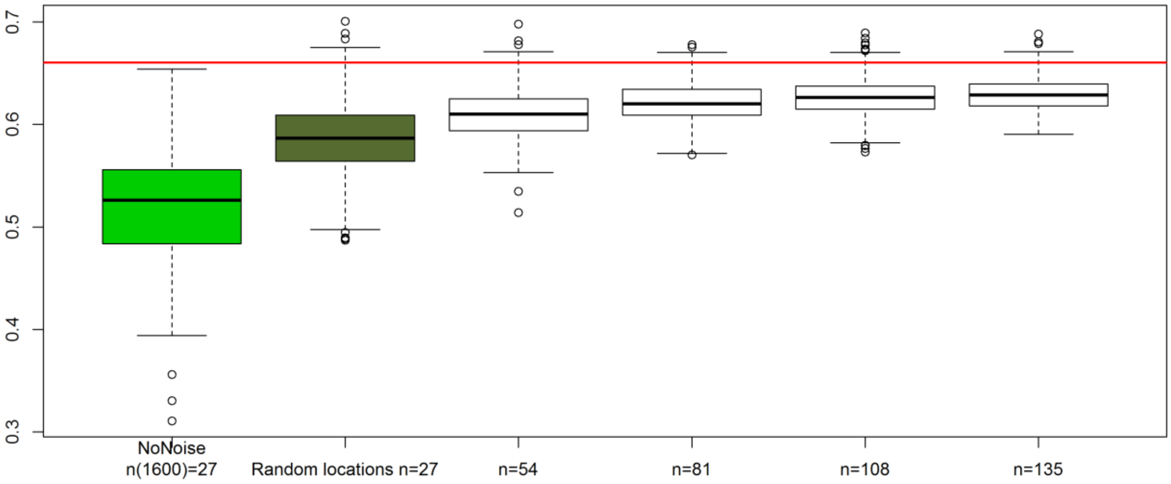


*Figure S23: Effect of the number of proxies in the SH on the present-day – LIA amplitude in the HadCM3 simulation. The two boxes on the left (NoNoise and Random locations n=27 in the Year 1600 CE) are based on the same experiment as shown in Figure 3 in the main text. The other boxplots are based on multiplying the number of randomly sampled proxy locations by 2, 3, 4 and 5. The red line marks the amplitude of the model truth.*

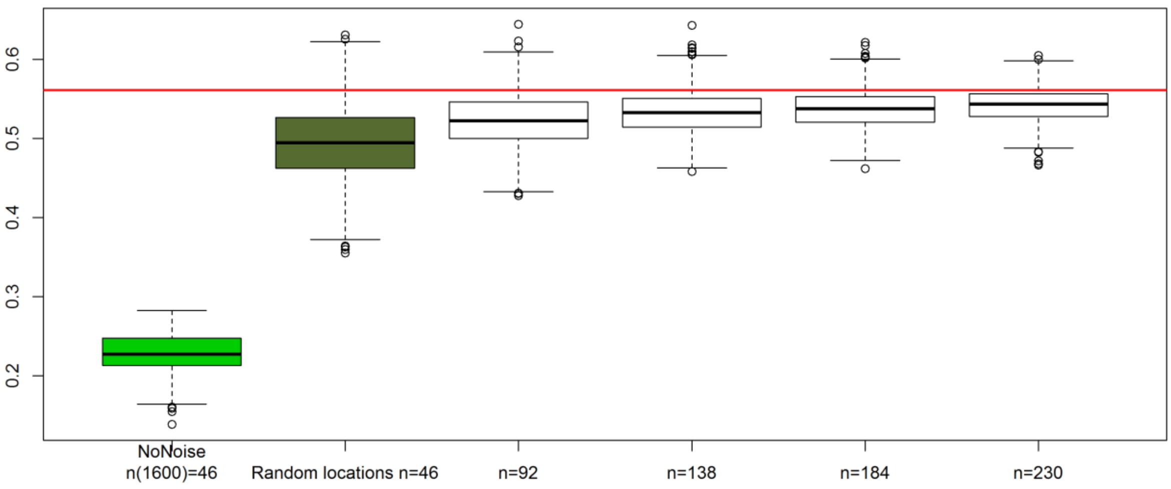


*Figure S24: Same as Figure S23 but for the NH.*

## 6. North – South correlations and differences

Figure S25 compares the NH vs. SH differences and correlations with the results from ref.[2], who used a large ensemble (n=524) of NH reconstructions based on different reconstruction techniques and proxy datasets, partially also including decadal-scale and lower frequency proxies[3]. The results are similar to the ones from the NH dataset used herein (black solid), providing further evidence that our results are robust to the choice of reconstruction method and proxy database.

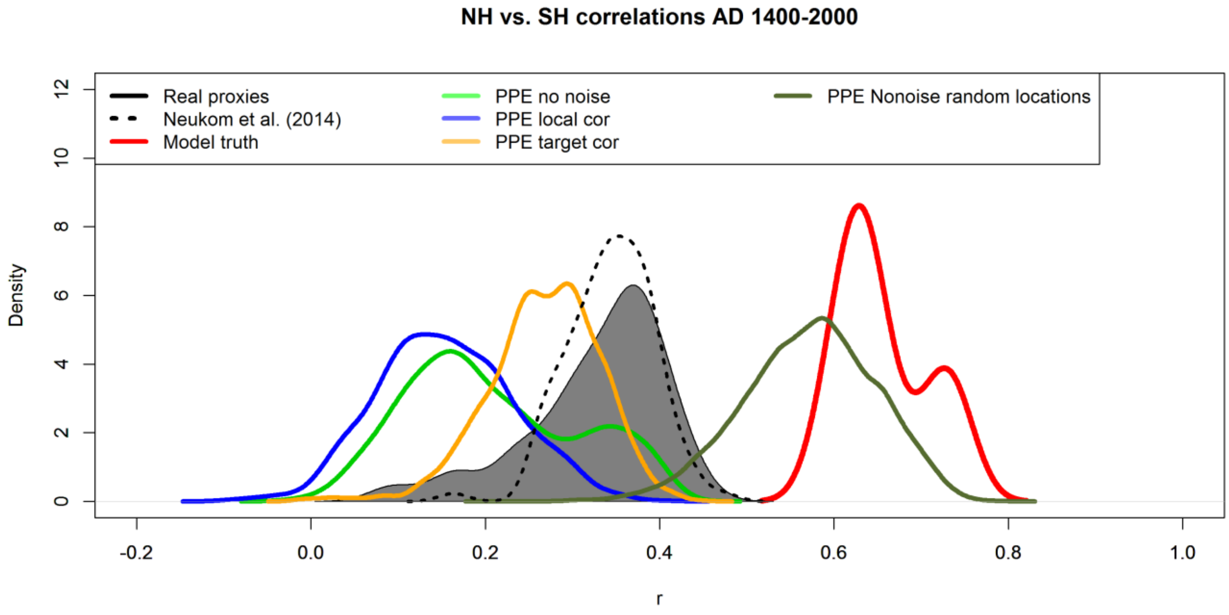


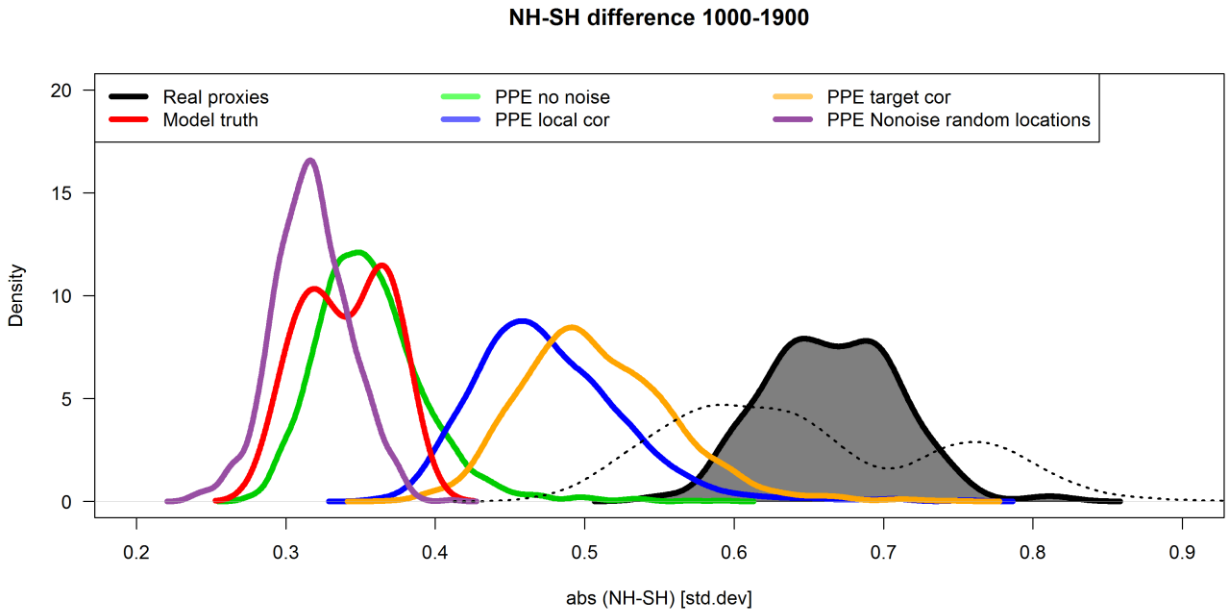


*Figure S25: Inter-hemispheric correlations (top) and differences (bottom), including the results from ref.[2] as dashed black line. Figure corresponds to Fig. 4 in the main text, but distributions are shown instead of boxplots.*

Figure S26 includes all PPE and shows that our conclusions are robust to the choice of optimal noise levels. Note that the SNR1t-AR1 experiment has unrealistically low noise levels, as shown by the strongly overestimated verification skill (Figure S10 and Figure S11). Only the AR1 experiments are able to generate NH-SH differences that are substantially different from the model truth.

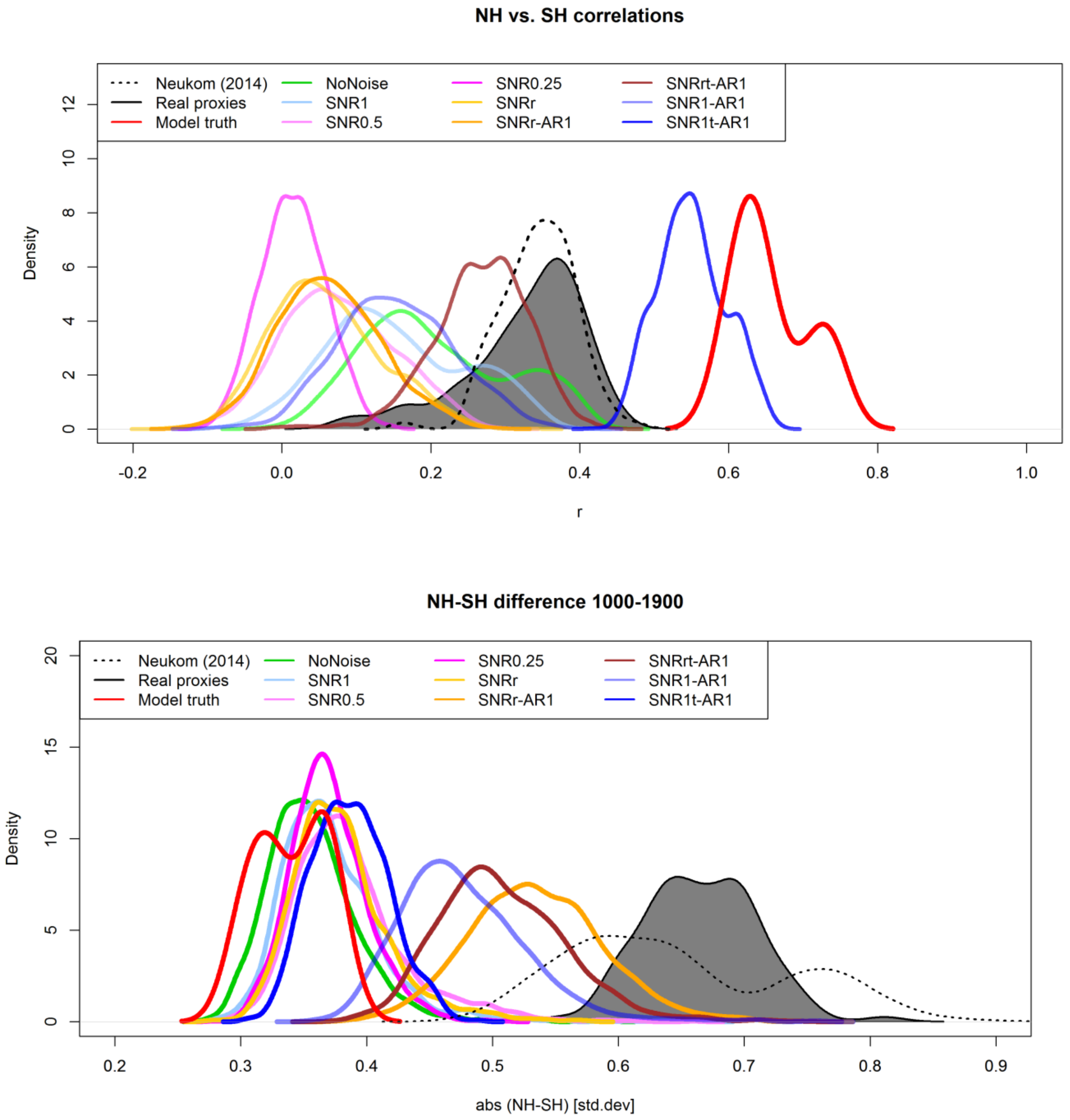


*Figure S26: Same as Figure S25 but including all experiments listed in Table S1.*

## 7. Extreme decades

Figure S27 shows the timing of global extreme decades as calculated as in ref.[2]. First, the fraction of decadally smoothed reconstruction ensemble members that exceed the ±1 std.dev. threshold is calculated for each hemisphere. Fractions from the SH and NH are then multiplied to yield global probabilities for extreme cold (blue) and warm (red) decades.

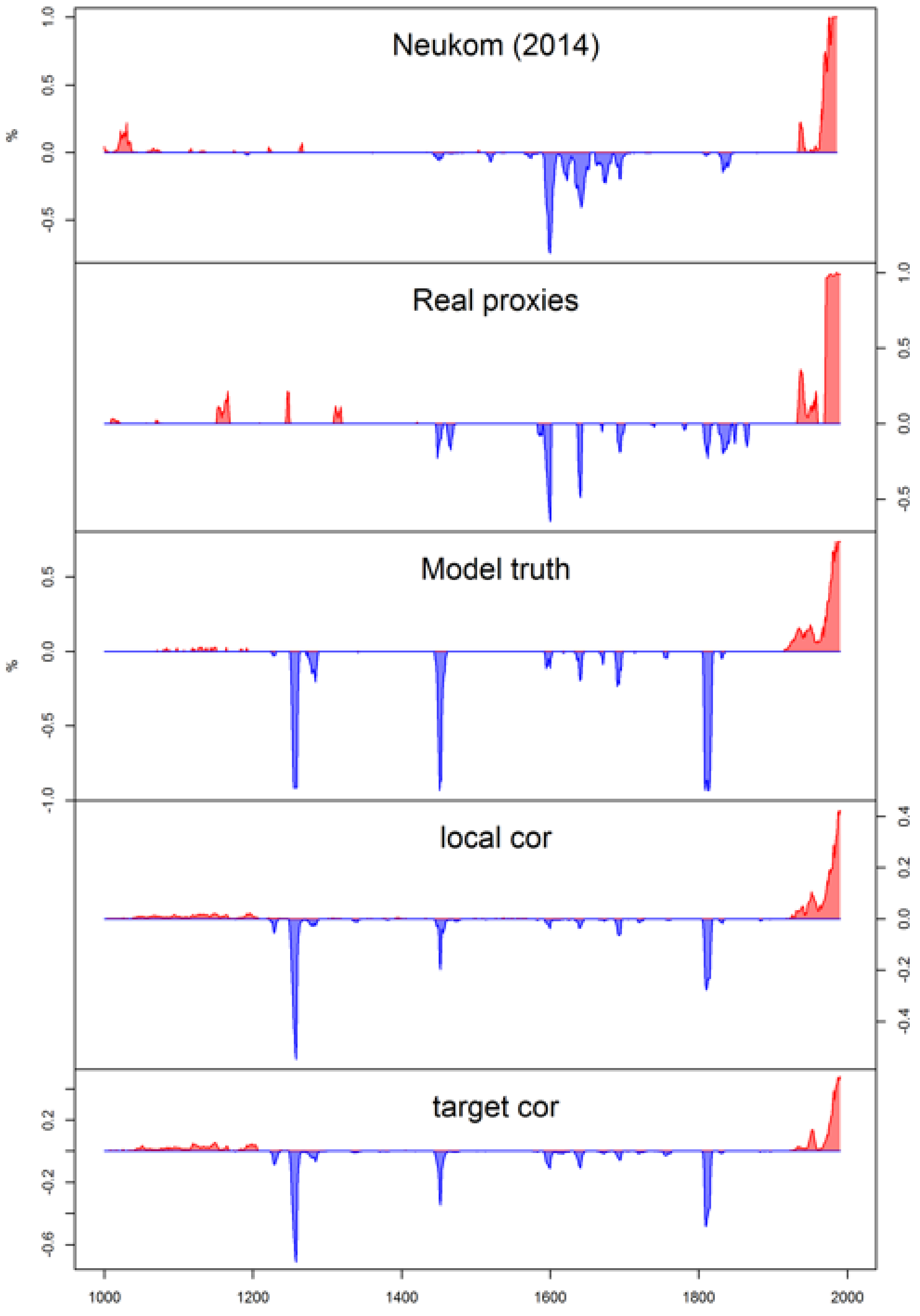


*Figure S27: Timing of extreme decades globally. See Figure 3 in ref.[2].*

## 8. Superposed Epoch Analysis for volcanic eruptions.

This section provides additional Figures for the SEA analysis.

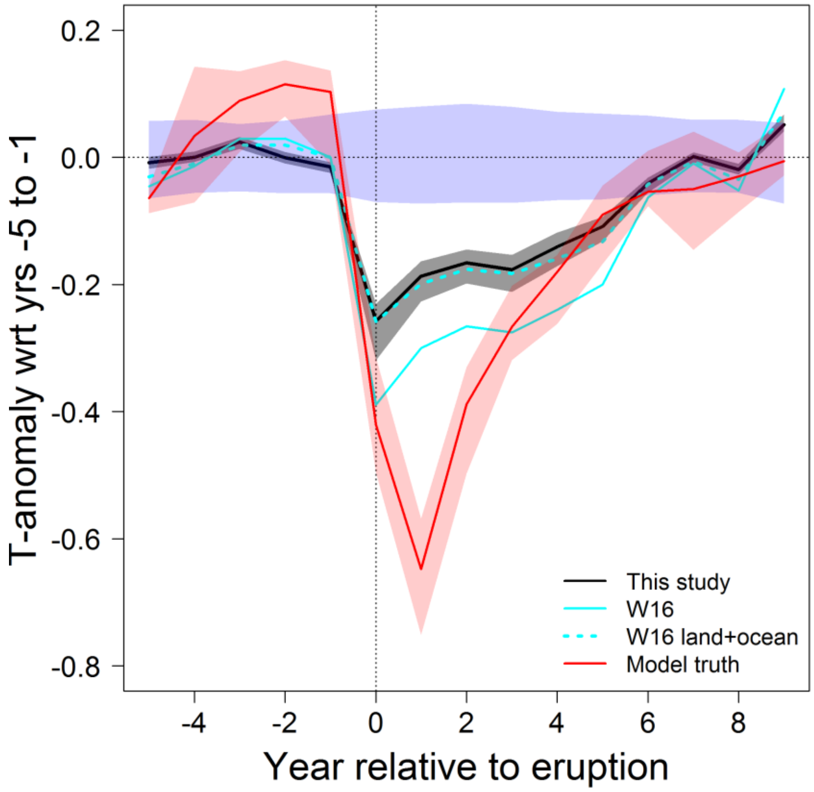


*Figure S28: Superposed epoch analysis of volcanic eruptions for the real-world proxies and model truth in the NH (same as top right panel in Figure 5 in the main text). Data of ref [1] are shown in cyan color: solid using their original land-only reconstruction target and dashed scaled to the combined land-ocean target used herein.*

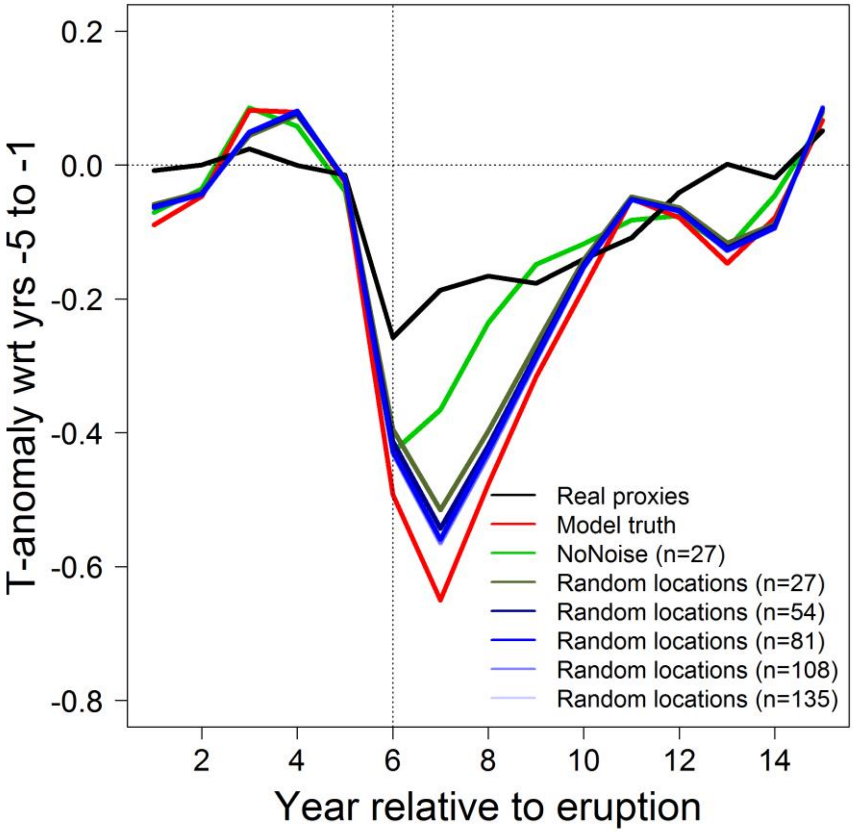


*Figure S29: Superposed epoch analysis of volcanic eruptions for the real-world proxies, model truth and NoNoise PPE in the NH (same as right panel in the second row of Figure 5 in the main text). Figure includes data from NoNoise PPE based on randomly sampled locations using the same number of proxies as in the real world (olive green) and multiples of this number (blue lines). n refers to the number of proxies at 1600 CE. Results for n>27 are nearly identical and thus hardly distinguishable in the plot. The Figure shows that increasing the number of proxies has a minor effect on the results of the SEA.*

## 9. Additional plots for the D&A scale factors

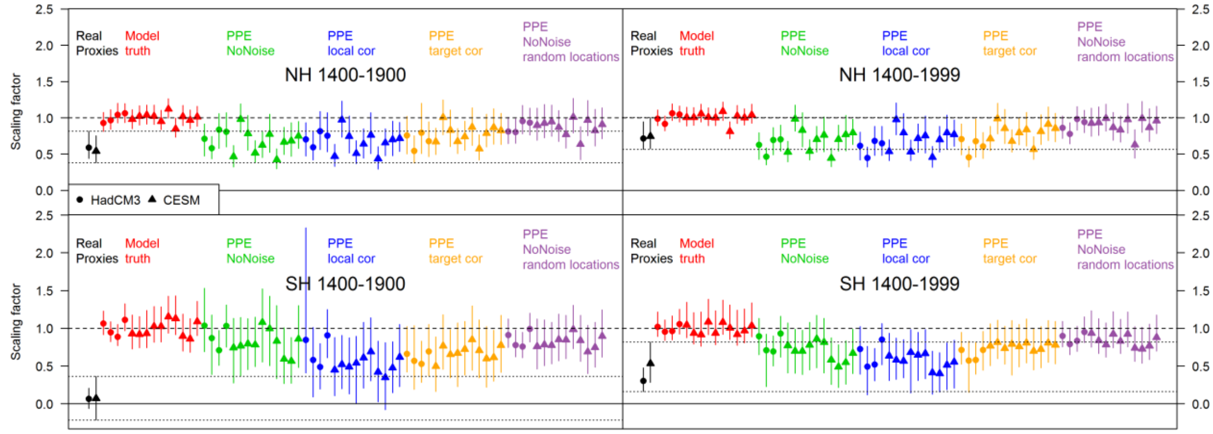


Figure S30: Same as Figure 6 in the main text but including also the period 1400-1999 for the D&A analysis (right panels). The stronger response to forcing during this period is due to the strong and common anthropogenic warming trend in models and reconstructions in both hemispheres.

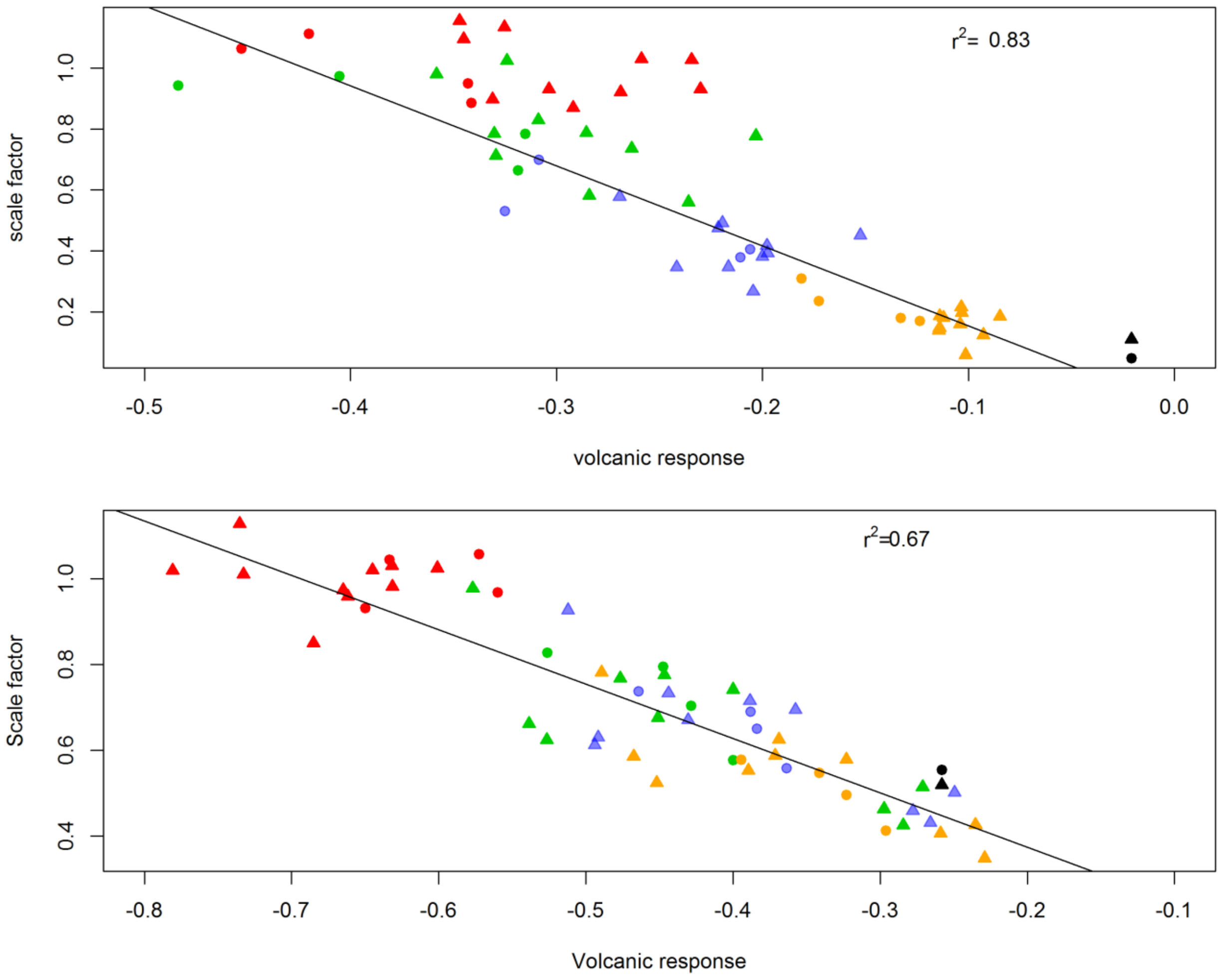


Figure S31: D&A scale factors (1400-1900) vs. volcanic response in SH (top) and NH (bottom). Colours mark the different experiments as in Figure 7 in the main text.

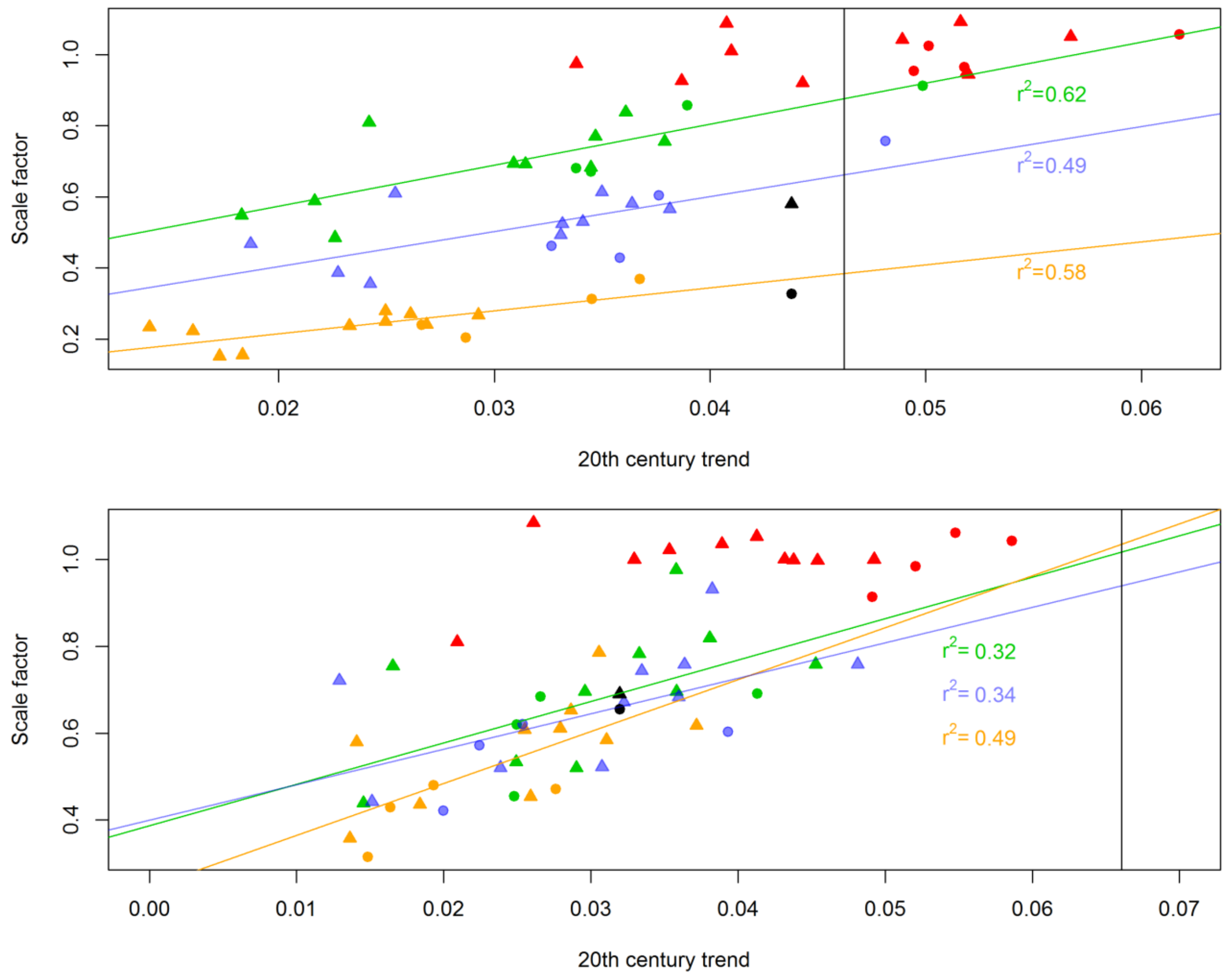


*Figure S32: D&A scale factors over 1400-1999 vs. 20th century trend in SH (top) and NH (bottom). Colours mark the different experiments as in Figure 7 in the main text. Black vertical lines are the instrumental data for the trend.*

## 10. Alternative reconstruction methods.

In this section we present plots based on the alternative reconstruction methods PaiCO[13] and BHM[14].

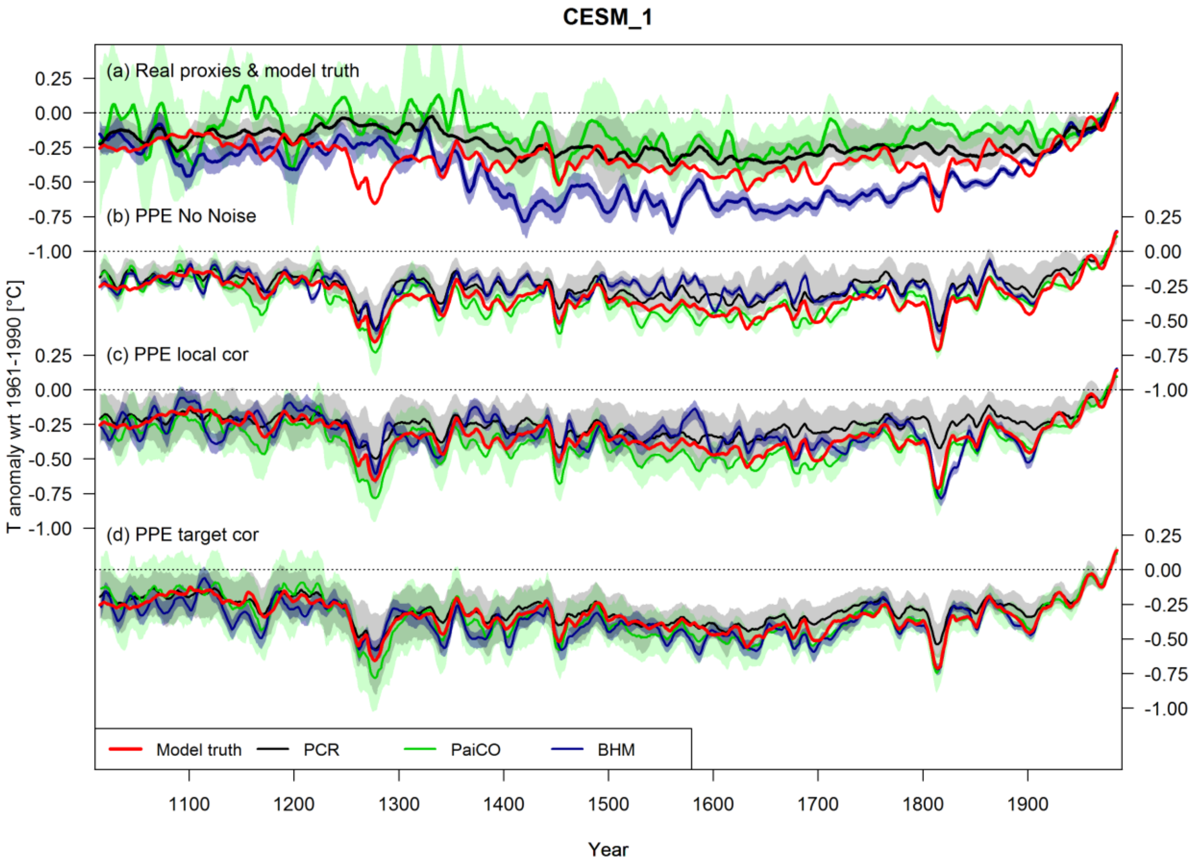


*Figure S33: SH real proxy (a) and PPE (b-d) temperature reconstructions using PCR (black) PaiCO (green) and BHM (blue) reconstruction methods. Ensemble uncertainties are shaded. Red line marks the model truth. Model data and PPE are from the CESM1-CAM5 (member 1) simulation.*

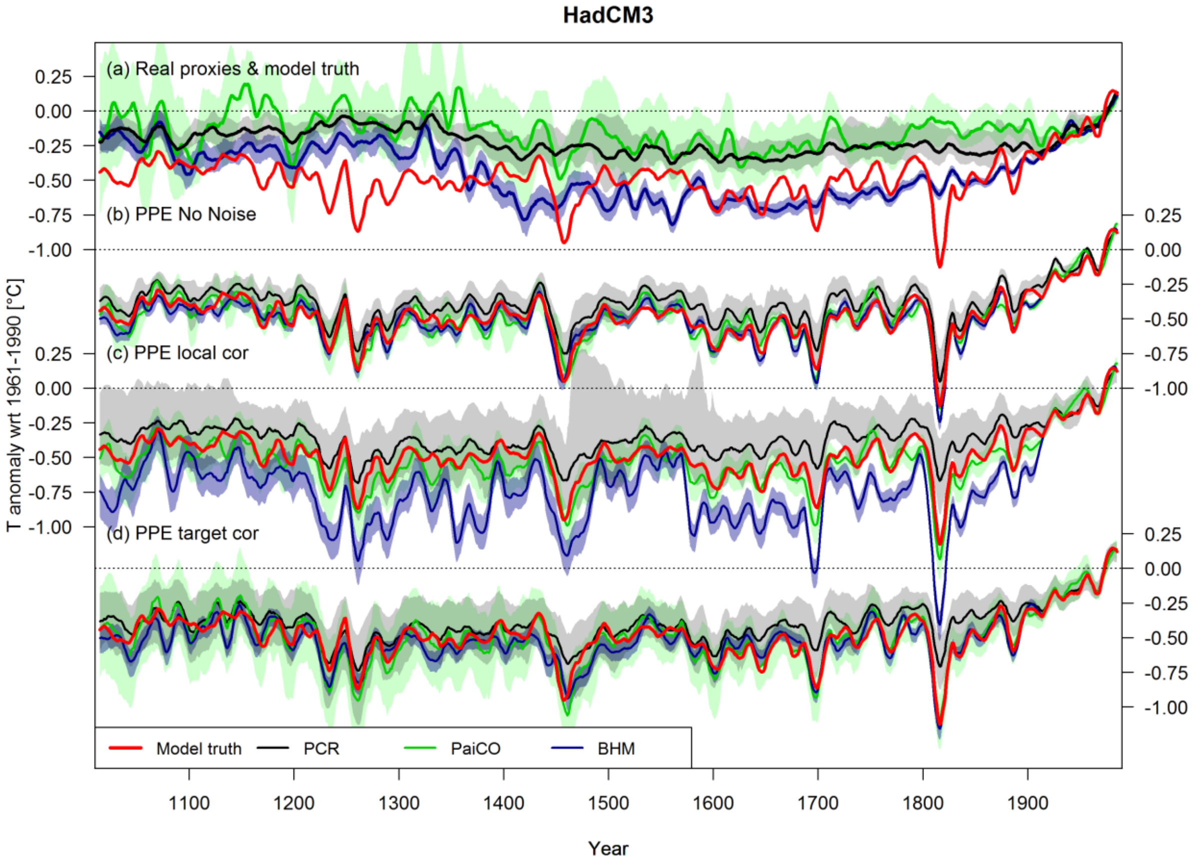


*Figure S34: Same as Figure S33 but for the HadCM3 last millennium simulation.*

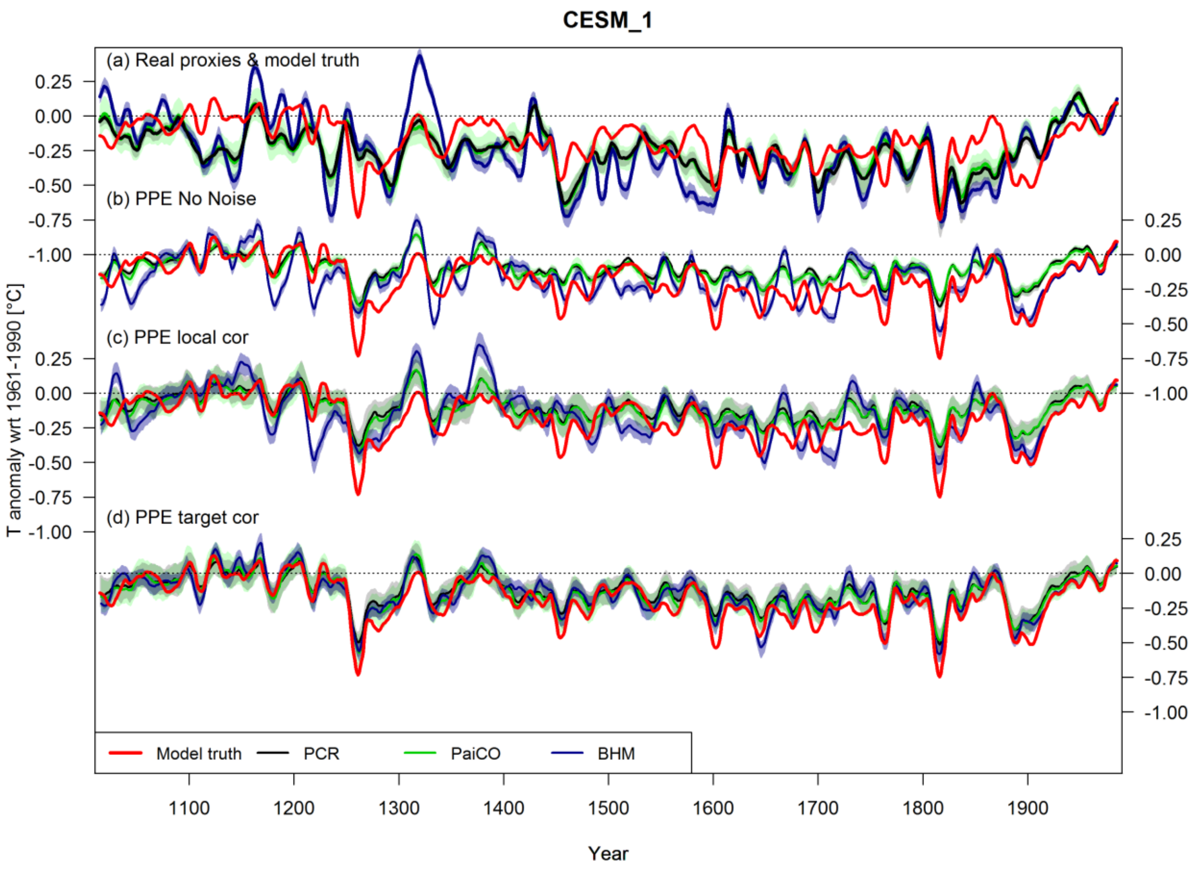


*Figure S35: Same as Figure S33 but for the NH.*

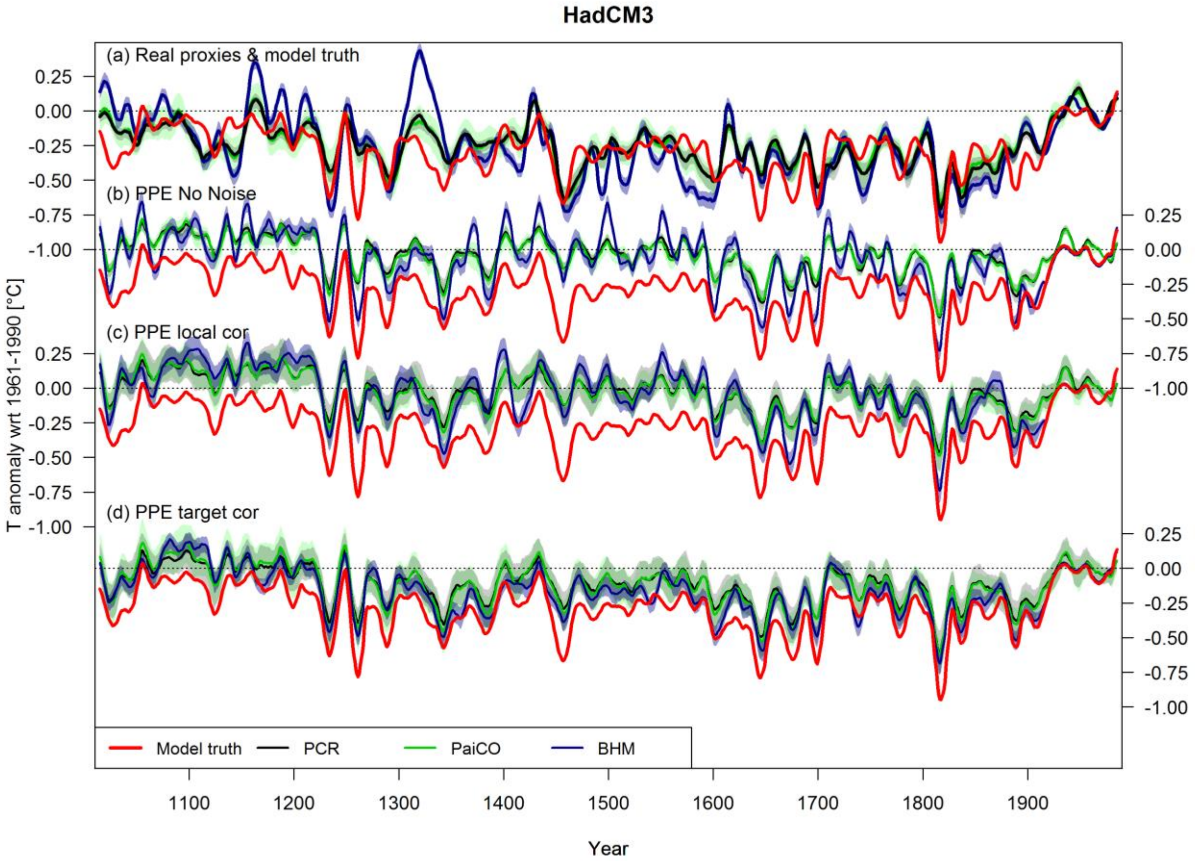


*Figure S36: Same as Figure S34 but for the NH.*

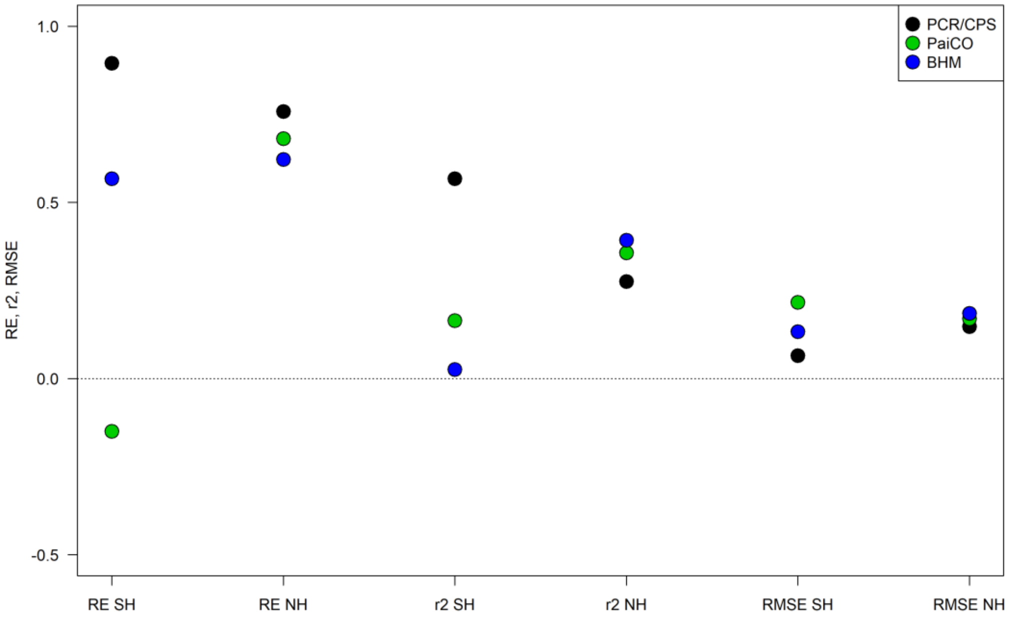


*Figure S37: Early verification skill of Real proxy reconstructions using the standard methods (PCR in the SH, CPS in the NH), and the alternative reconstruction methods PaiCO (green) and BHM (blue). In contrast to the other Figures showing reconstruction skill, results are only for the most replicated nest (including all proxies). Note that for RE and r² higher numbers mean better skill, whereas for RMSE it is the opposite.*

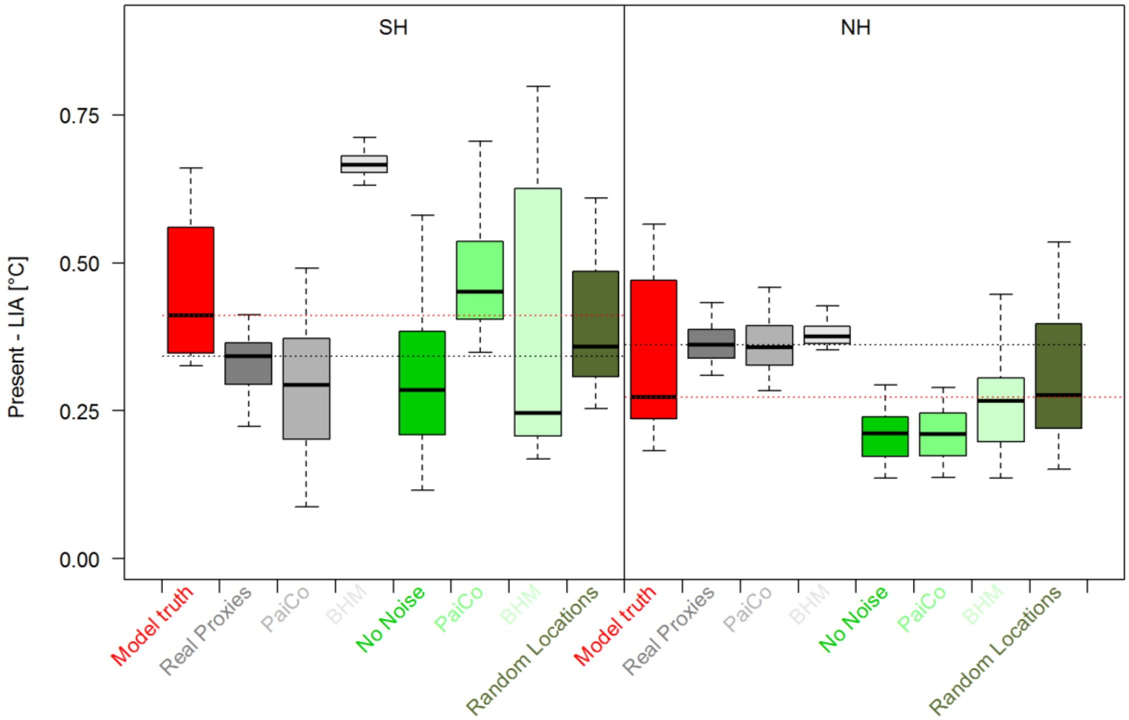


*Figure S38: Present-day minus LIA temperature amplitudes for different reconstruction methods. Same as Figure 3b in the main text but including results for PaiCO and BHM for the real proxy and NoNoise experiments.*

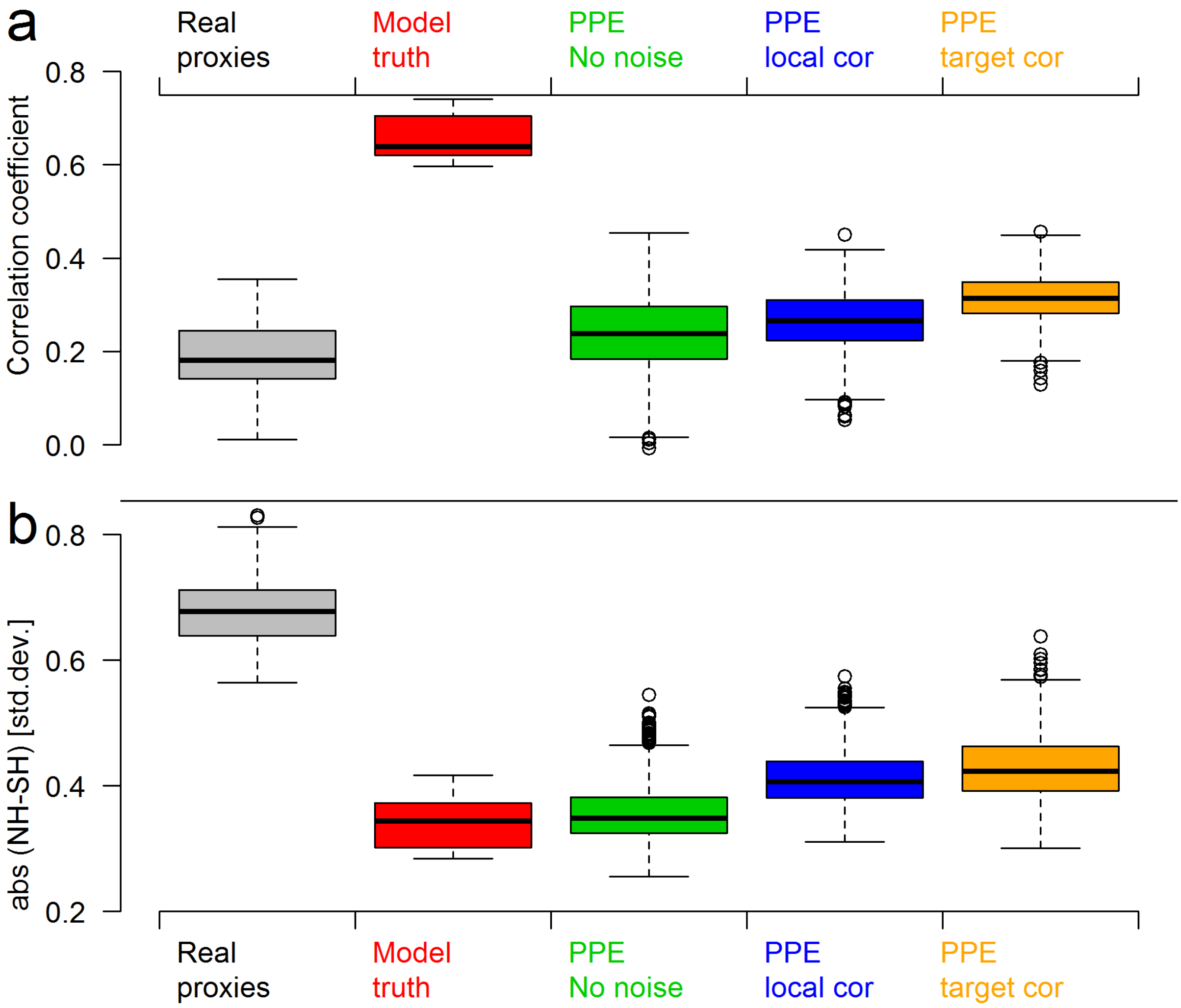


*Figure S39: Same as Figure 4 in the main text but based on the PaiCO reconstruction method.*

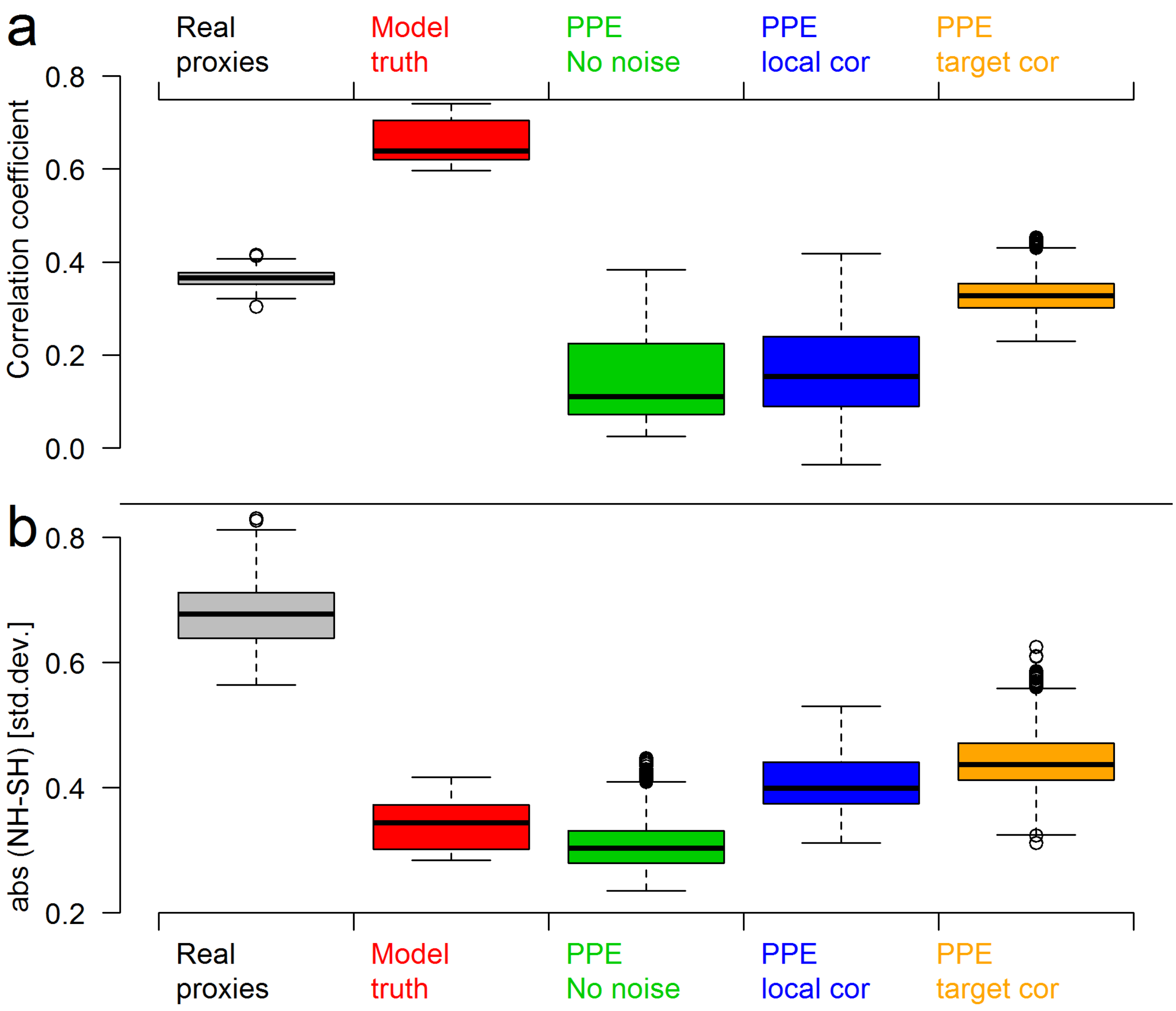


*Figure S40: Same as Figure 4 in the main text but based on the BHM reconstruction method.*

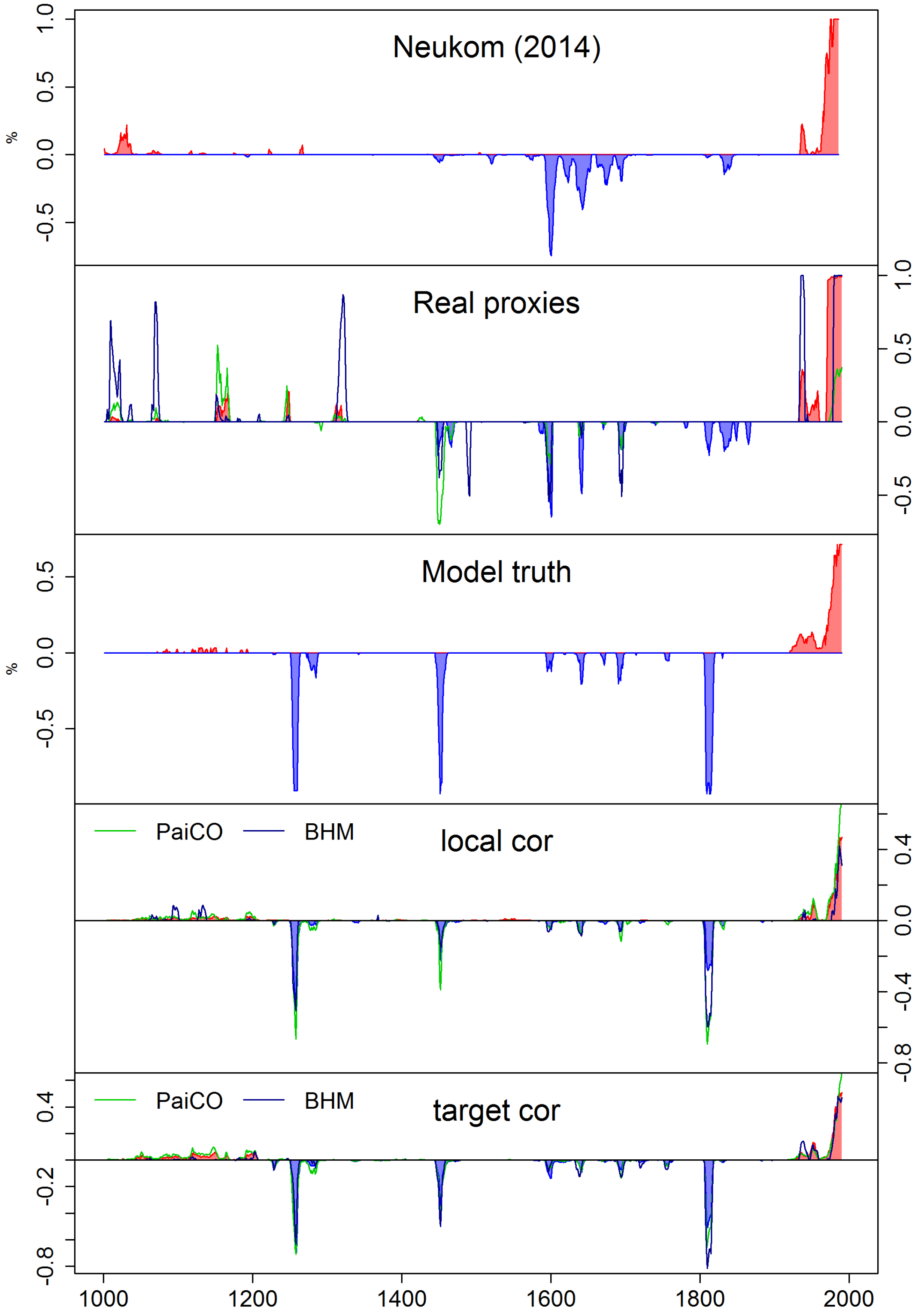


*Figure S41: Same as Figure S27 but including the results of the PaiCO (green line) and BHM (dark blue line) reconstruction methods.*

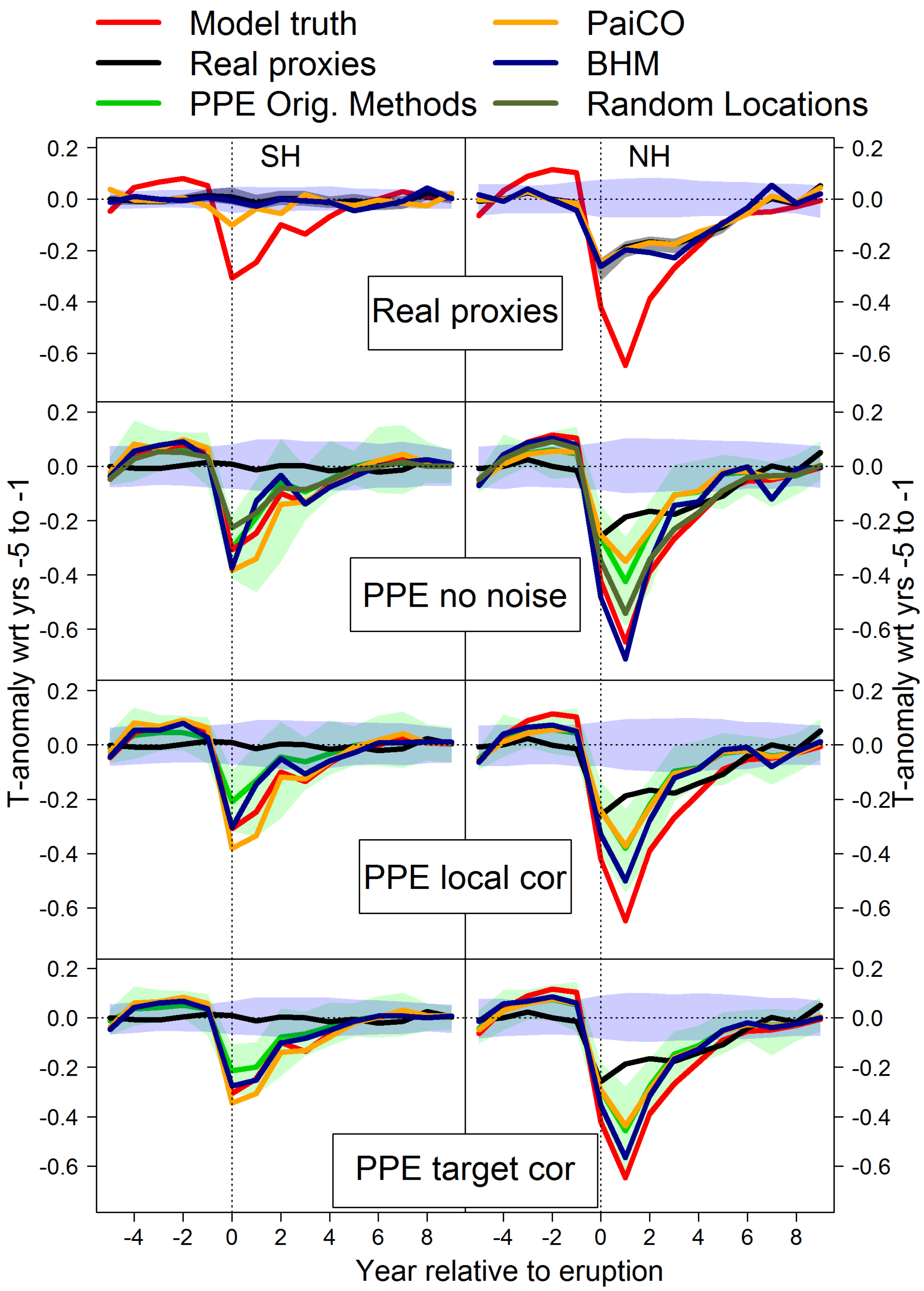


*Figure S42: Same as Figure 5 in the main text but showing also the results for the PaiCO and BHM reconstruction methods.*

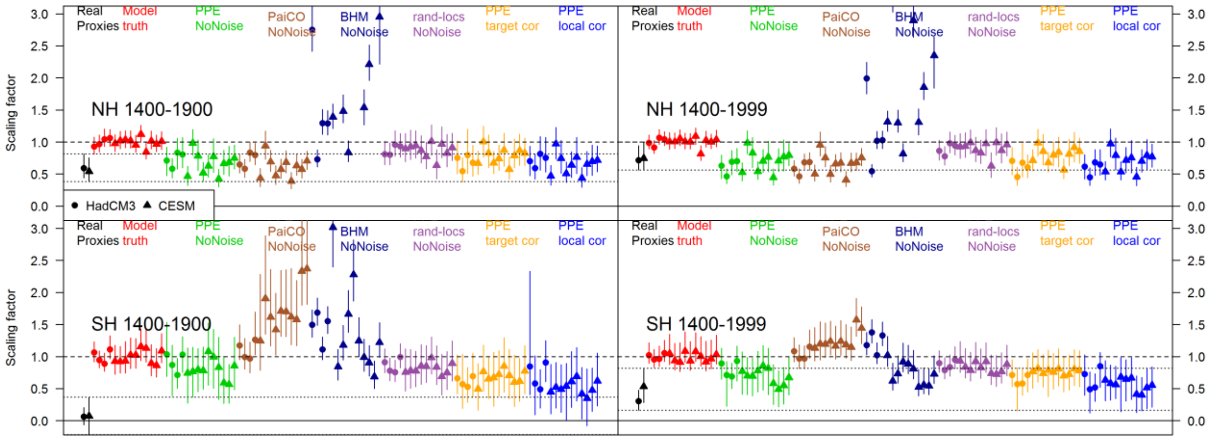


*Figure S43: D&A results including PaiCO and BHM reconstructions. Figure is analogous to Figure S30, the left panels correspond to the period shown in Figure 6 in the main text.*

## 11. Reconstruction time series for the individual model simulations

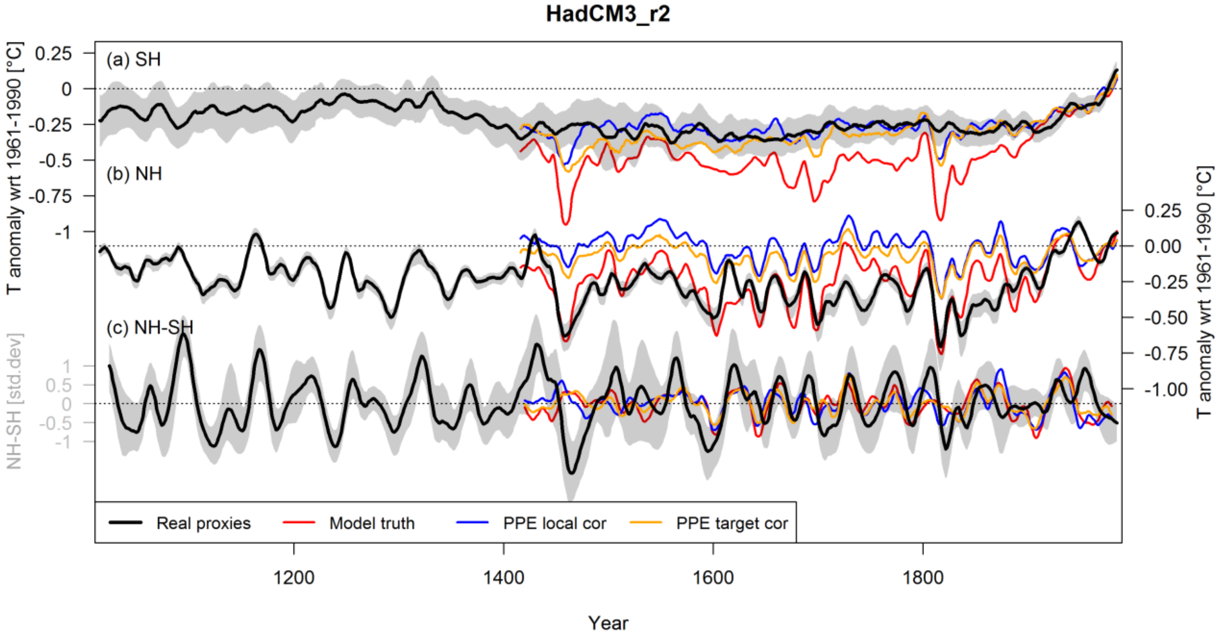


*Figure S44: Same as Figure 1 in the main text but for the simulation HadCM3 r2.*

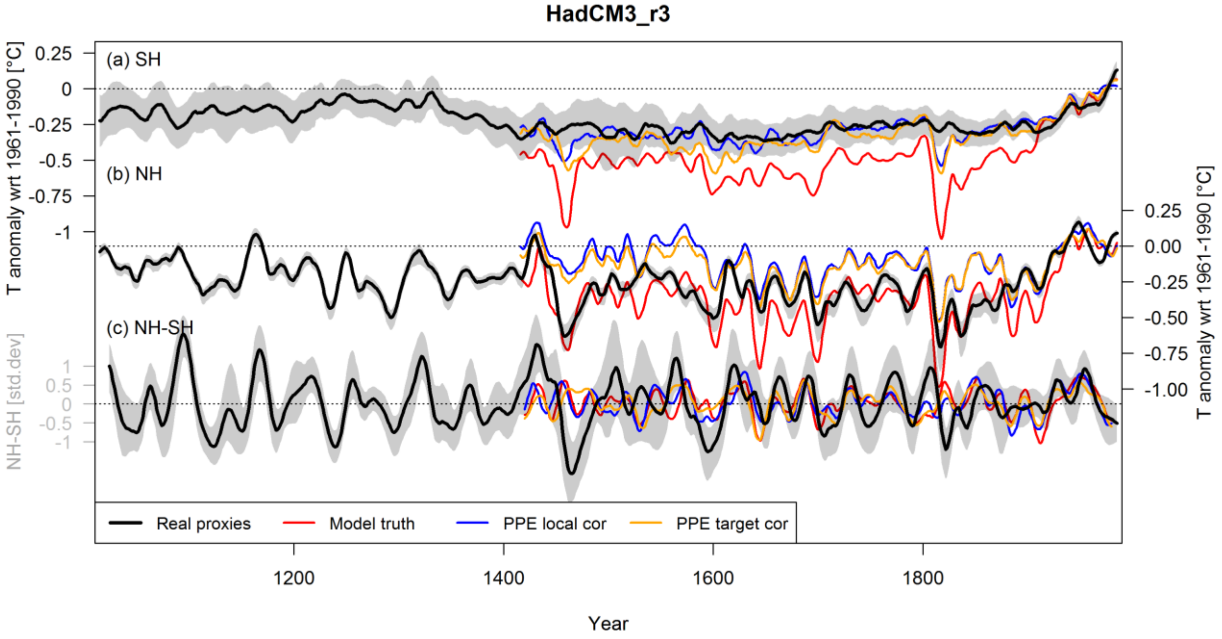


*Figure S45: Same as Figure 1 in the main text but for the simulation HadCM3 r3.*

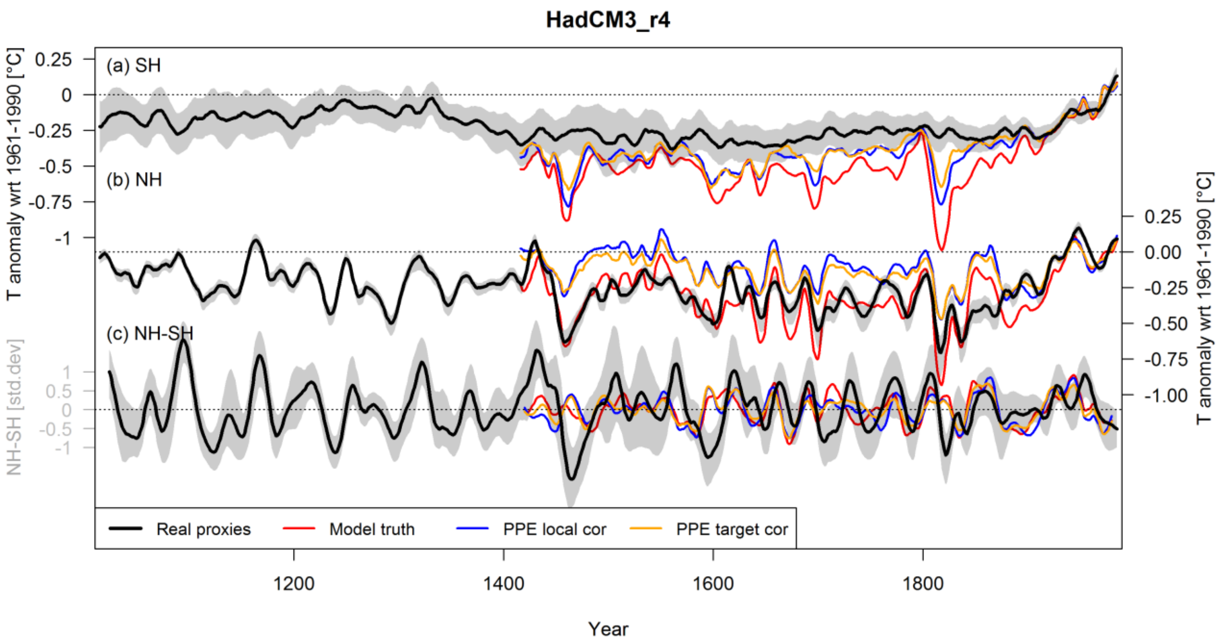


*Figure S46: Same as Figure 1 in the main text but for the simulation HadCM3 r4.*

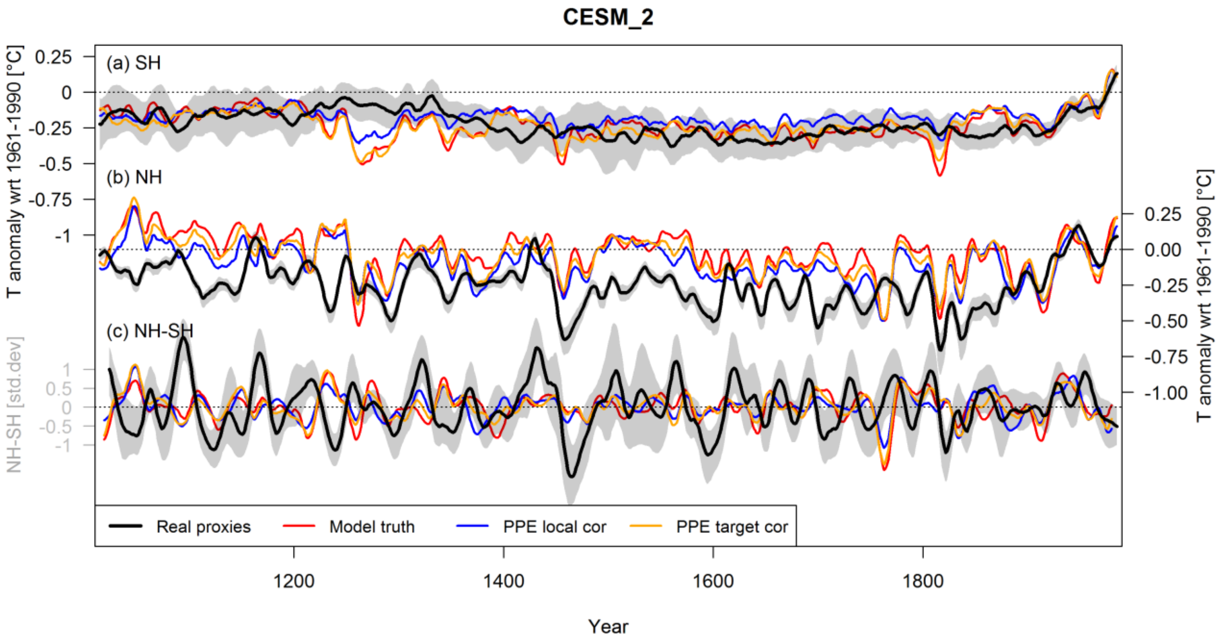


*Figure S47: Same as Figure 1 in the main text but for the simulation CESM1-CAM5 ensemble member 2.*

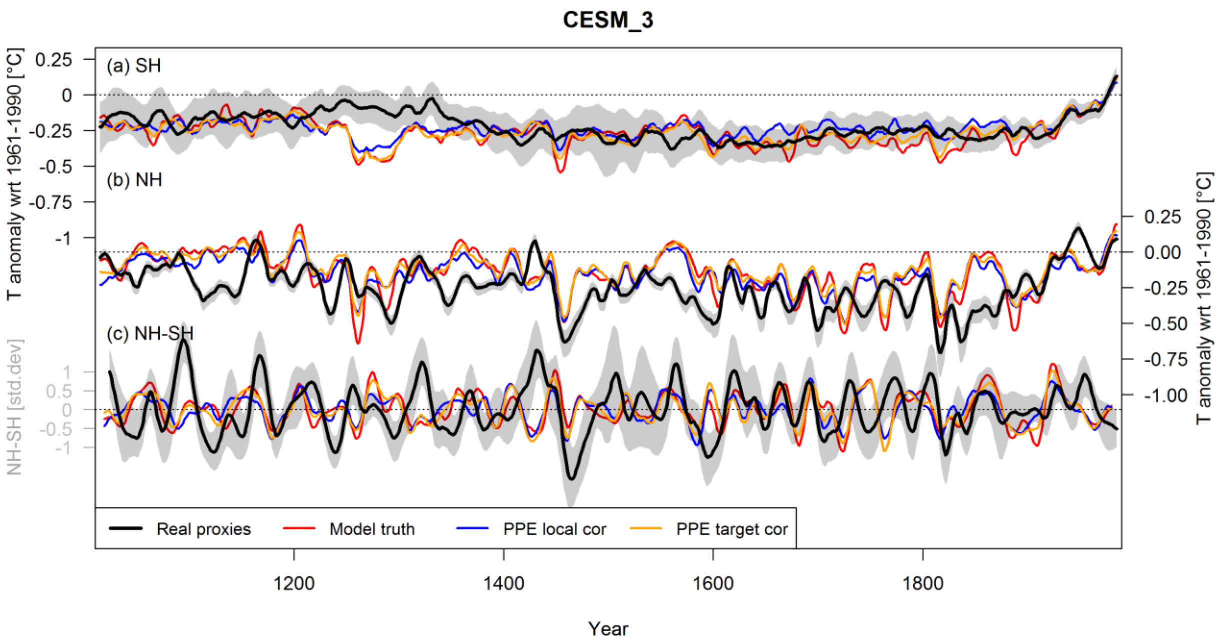


*Figure S48: Same as Figure 1 in the main text but for the simulation CESM1-CAM5 ensemble member 3.*

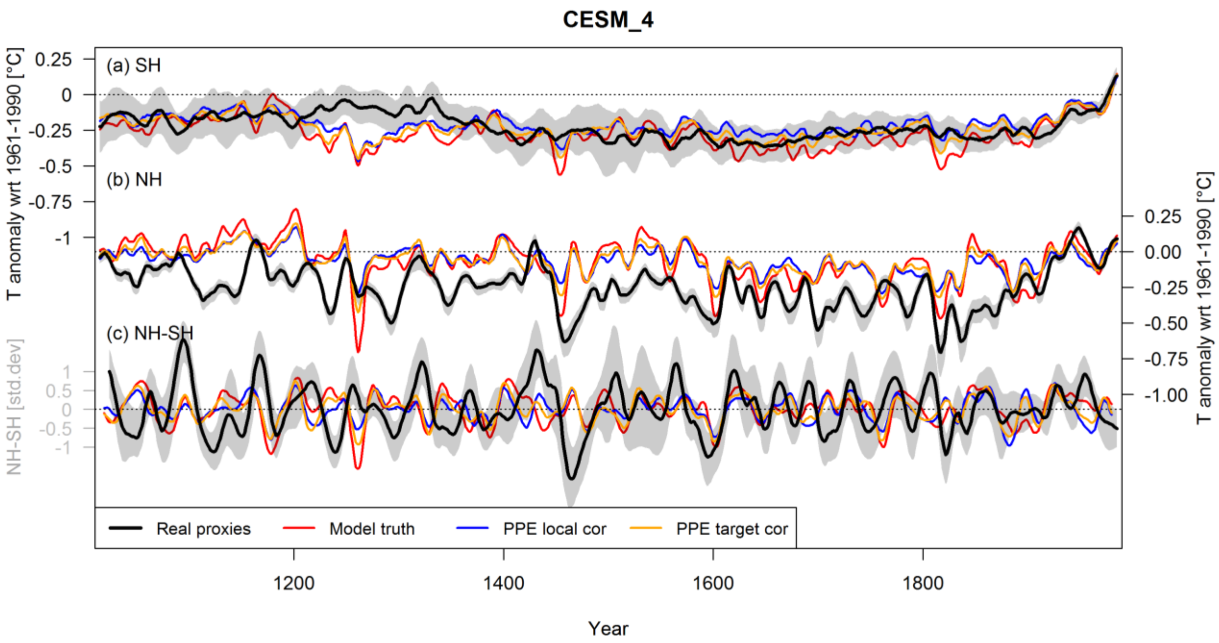


*Figure S49: Same as Figure 1 in the main text but for the simulation CESM1-CAM5 ensemble member 4.*

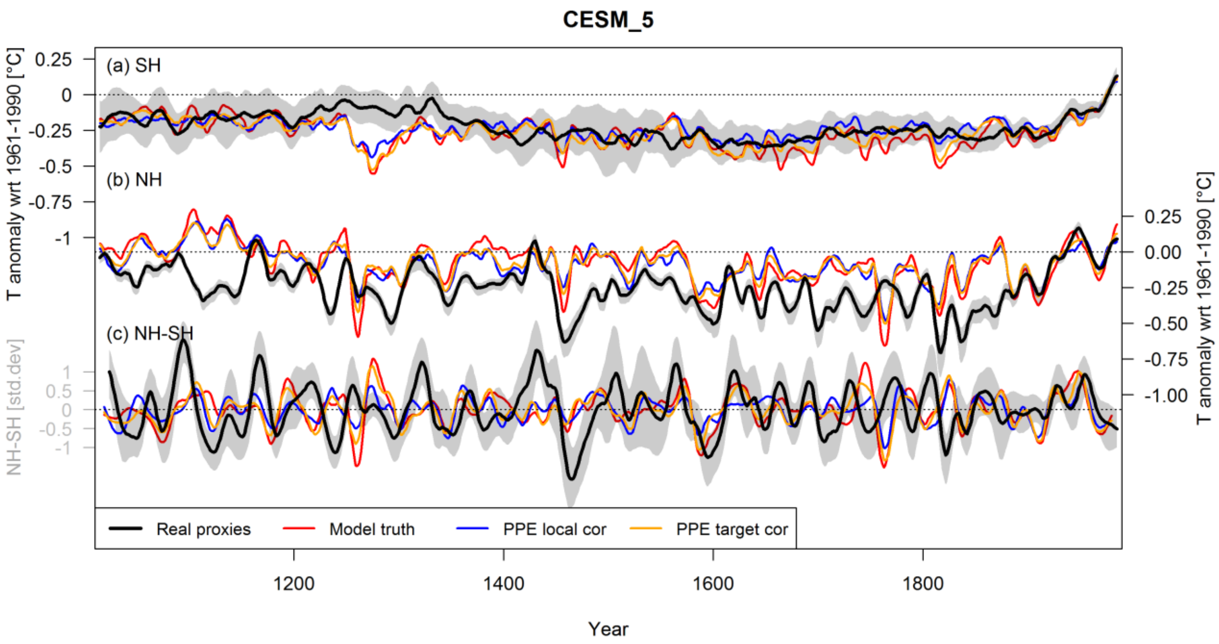


*Figure S50: Same as Figure 1 in the main text but for the simulation CESM1-CAM5 ensemble member 5.*

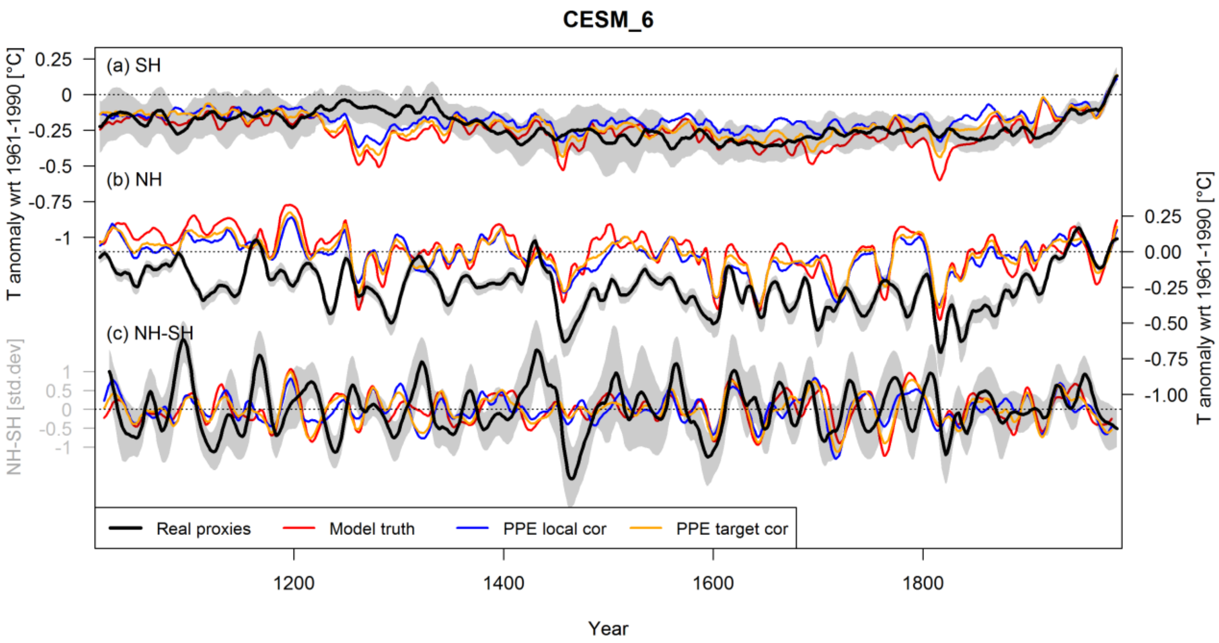


*Figure S51: Same as Figure 1 in the main text but for the simulation CESM1-CAM5 ensemble member 6.*

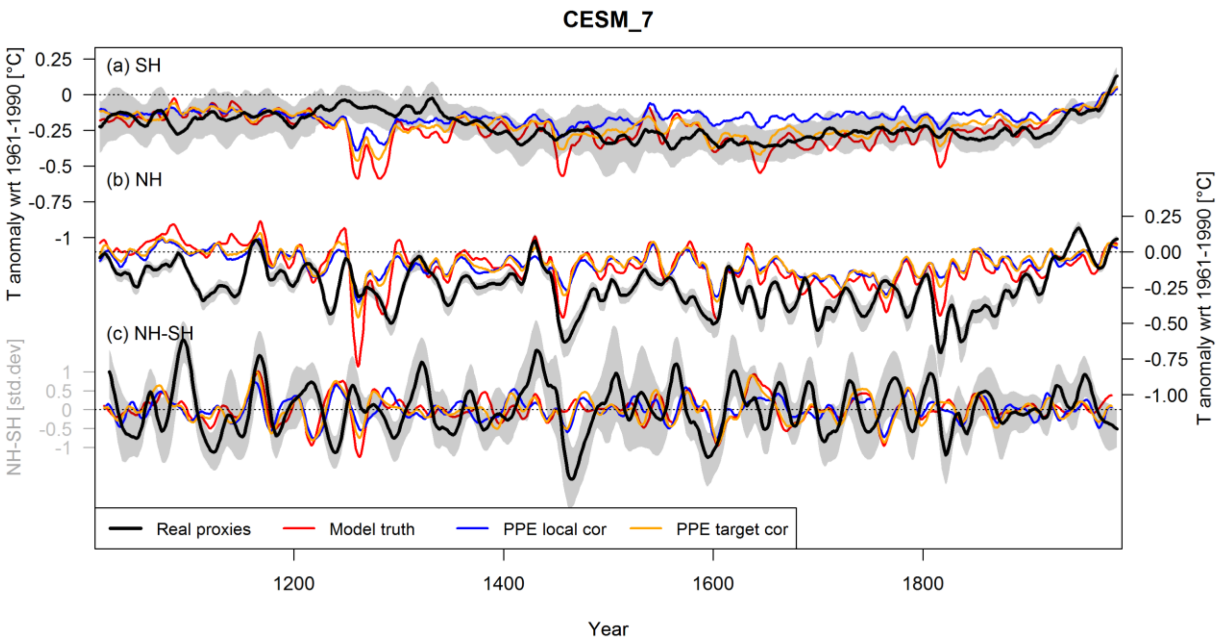


*Figure S52: Same as Figure 1 in the main text but for the simulation CESM1-CAM5 ensemble member 7.*

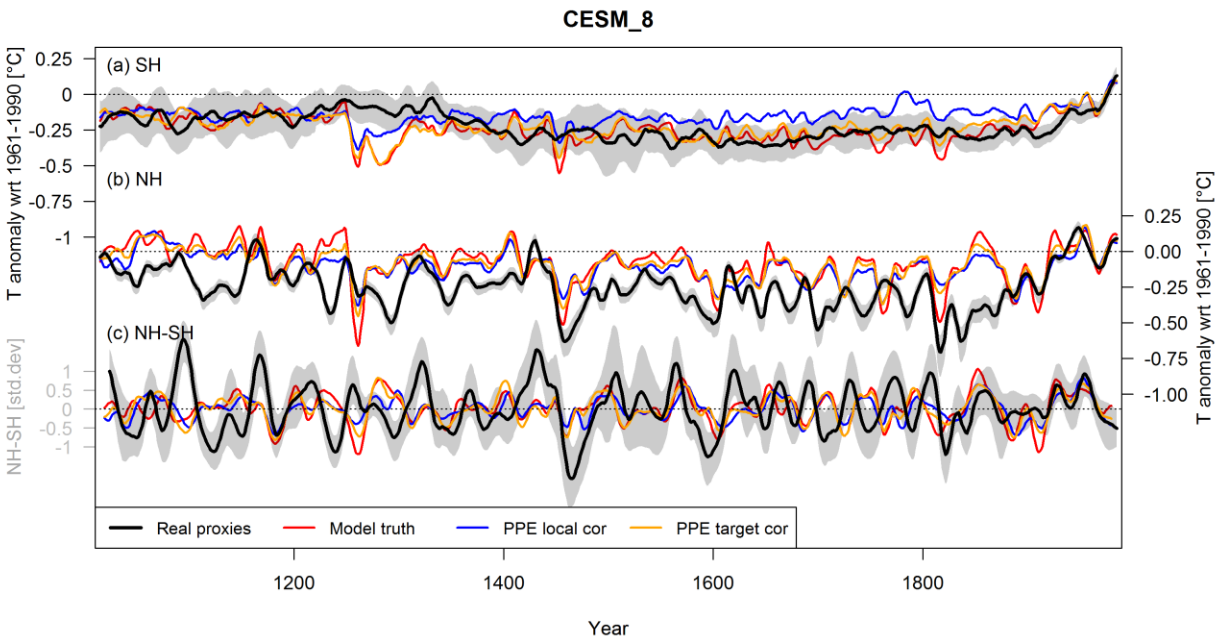


*Figure S53: Same as Figure 1 in the main text but for the simulation CESM1-CAM5 ensemble member 8.*

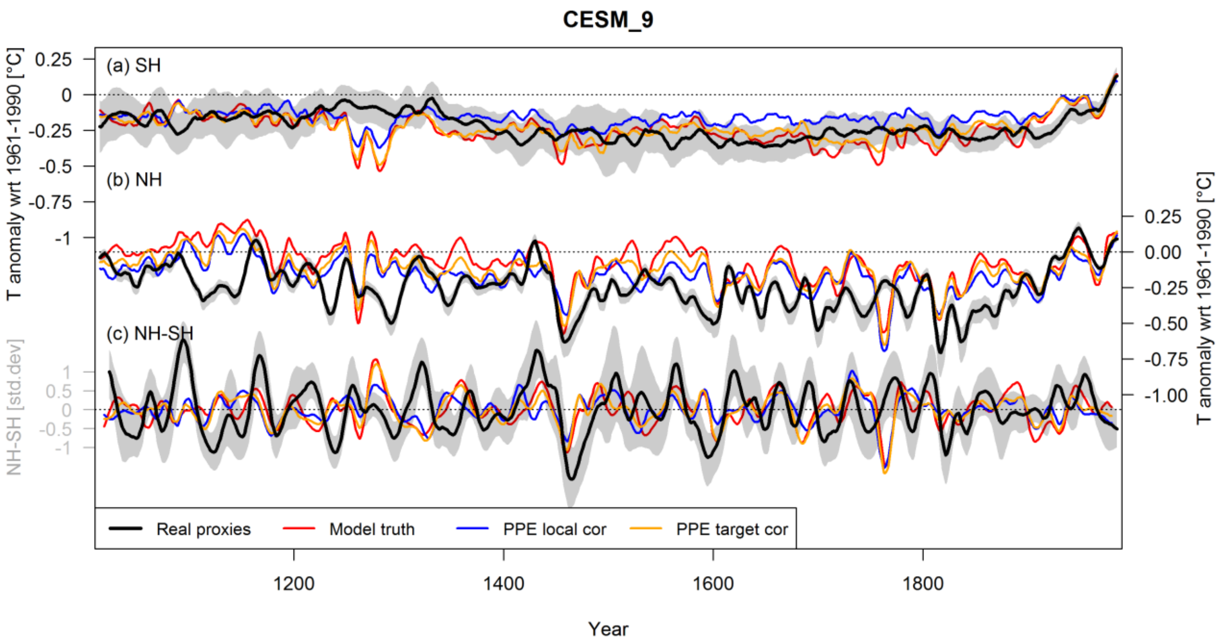


*Figure S54: Same as Figure 1 in the main text but for the simulation CESM1-CAM5 ensemble member 9.*

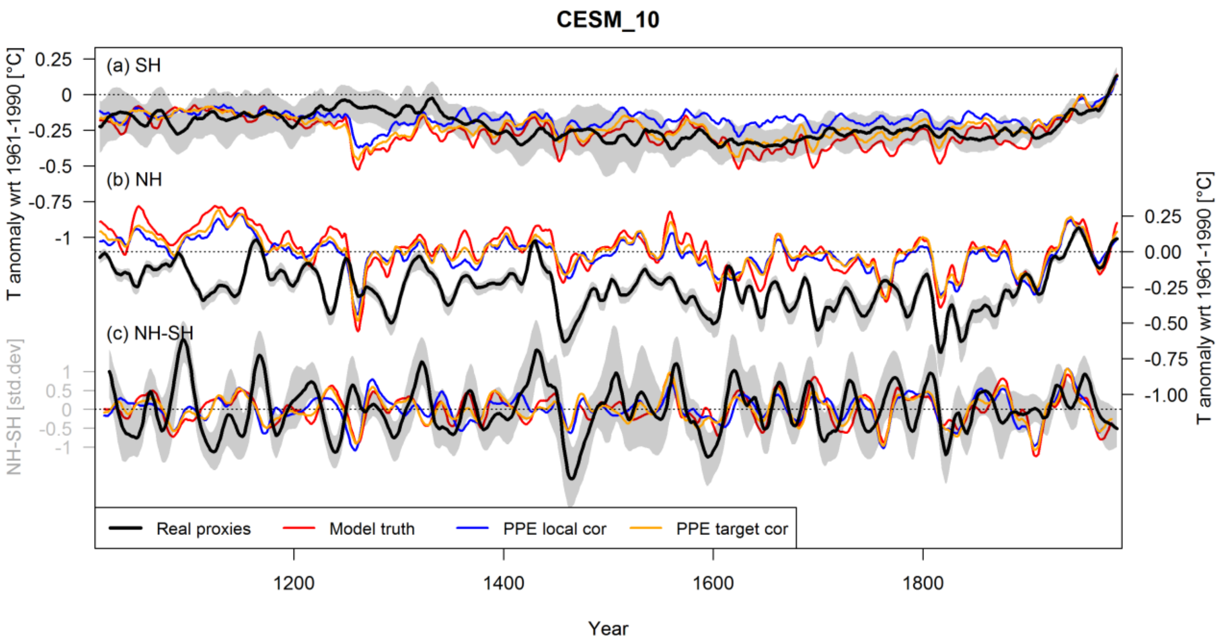


*Figure S55: Same as Figure 1 in the main text but for the simulation CESM1-CAM5 ensemble member 10.*

# 12. Skill plots for the individual model simulations

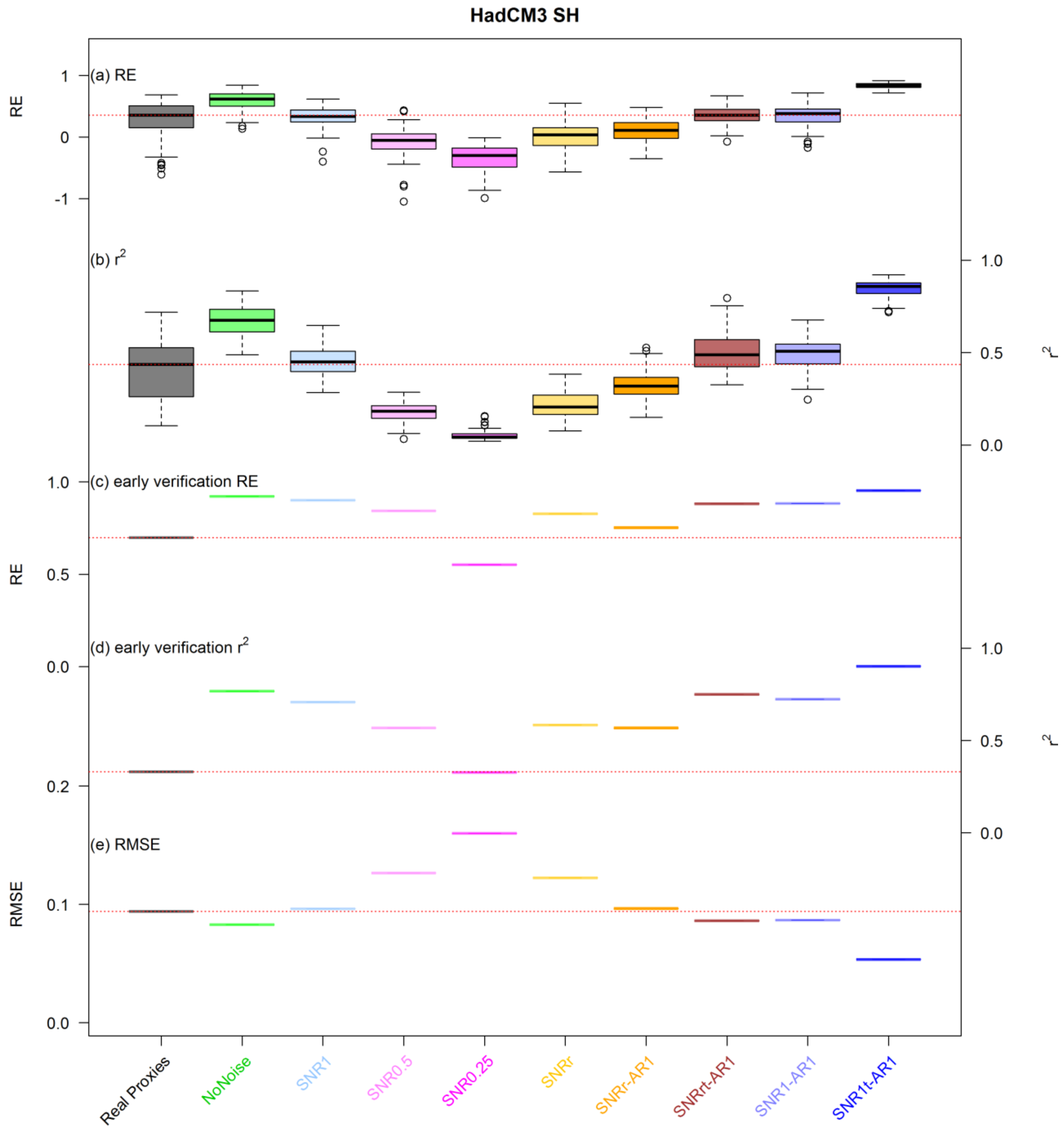


*Figure S56: Same as Figure S10 but for the HadCM3 simulation only. Note that the early verification and RMSE skill are calculated for the reconstruction ensemble mean, so yield only one value per PPE.*

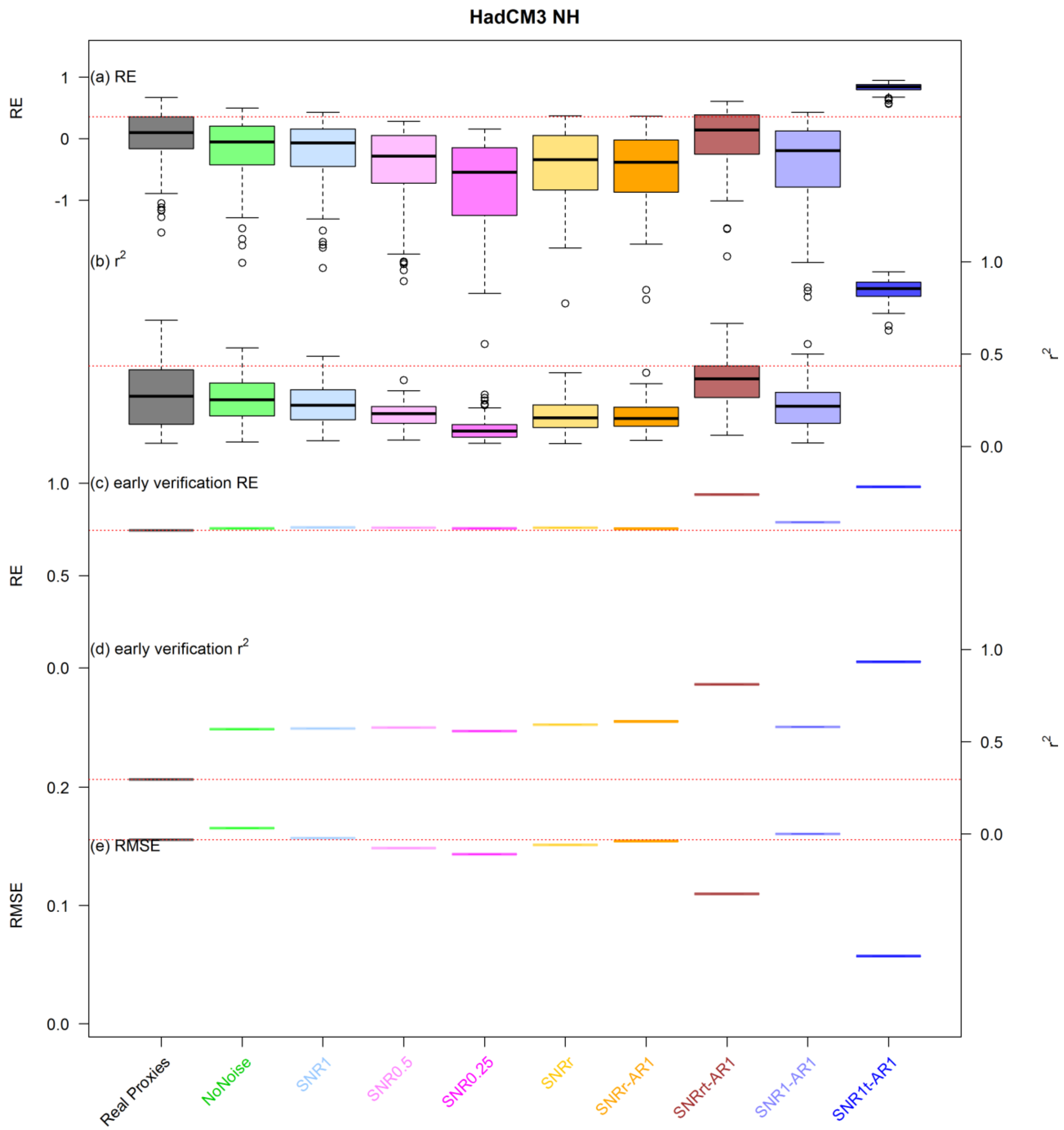


*Figure S57: Same as Figure S11 but for the HadCM3 simulation only.*

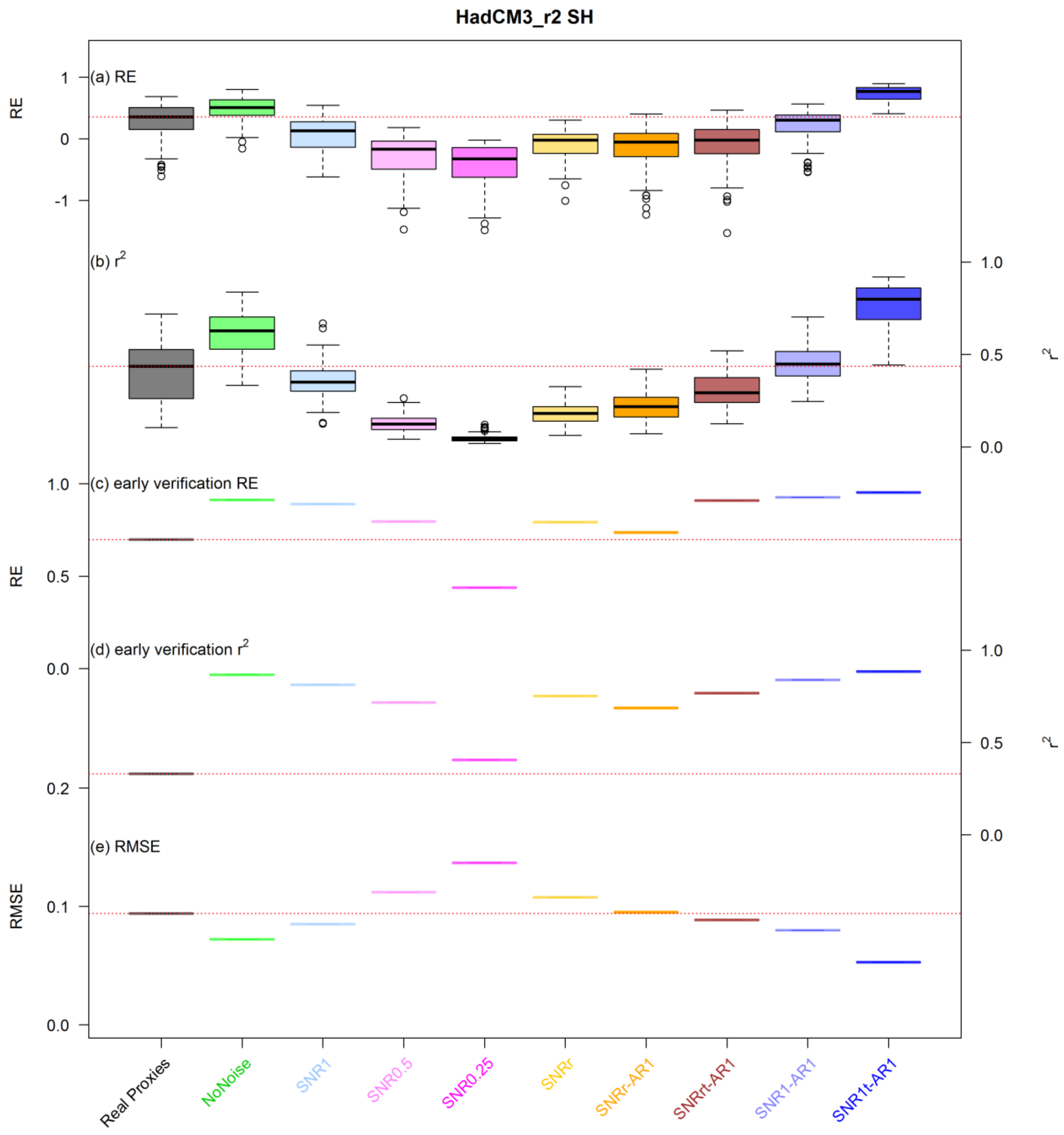


*Figure S58: Same as Figure S10 but for the HadCM3_r2 simulation only.*

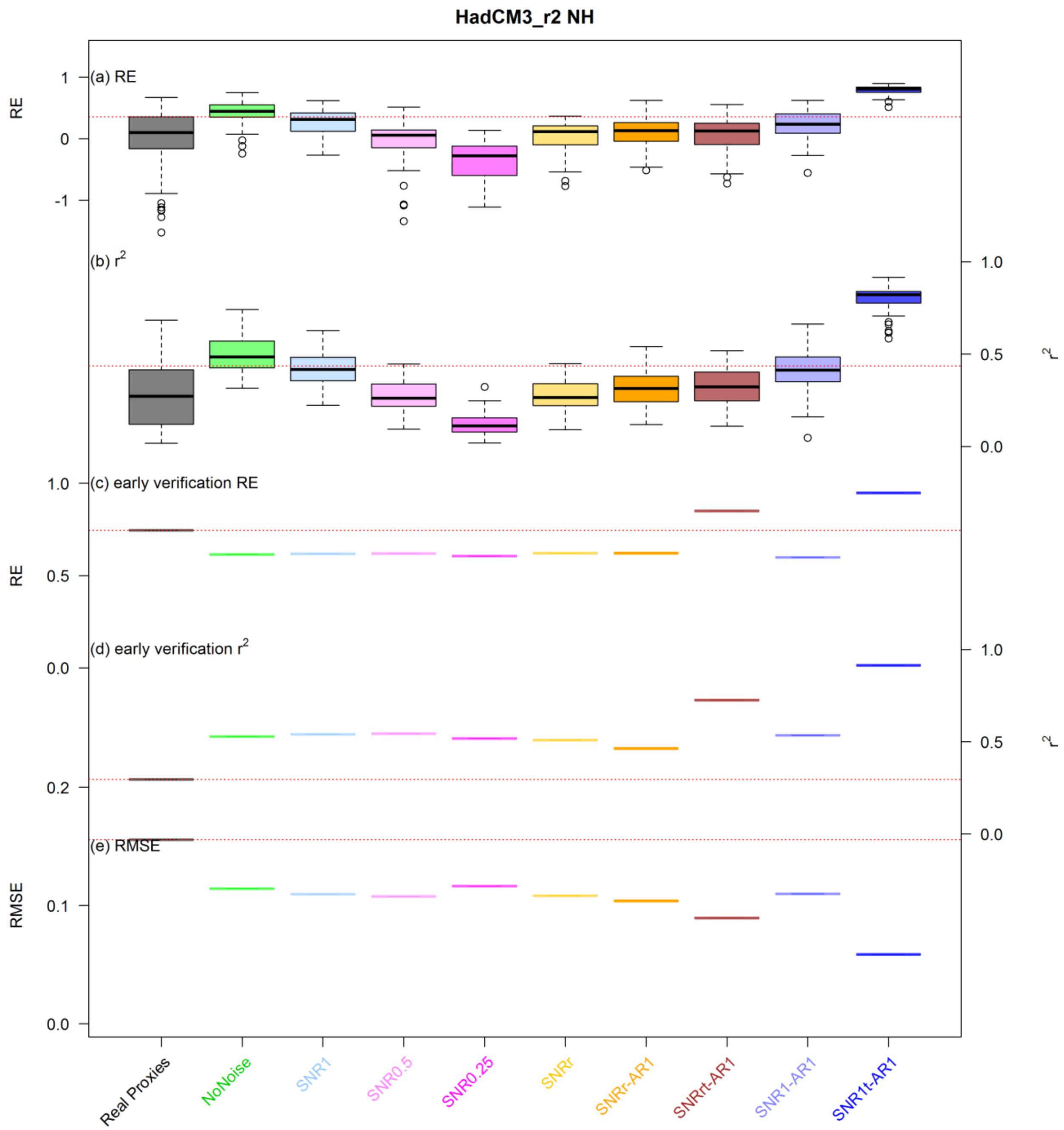


*Figure S59: Same as Figure S11 but for the HadCM3_r2 simulation only.*

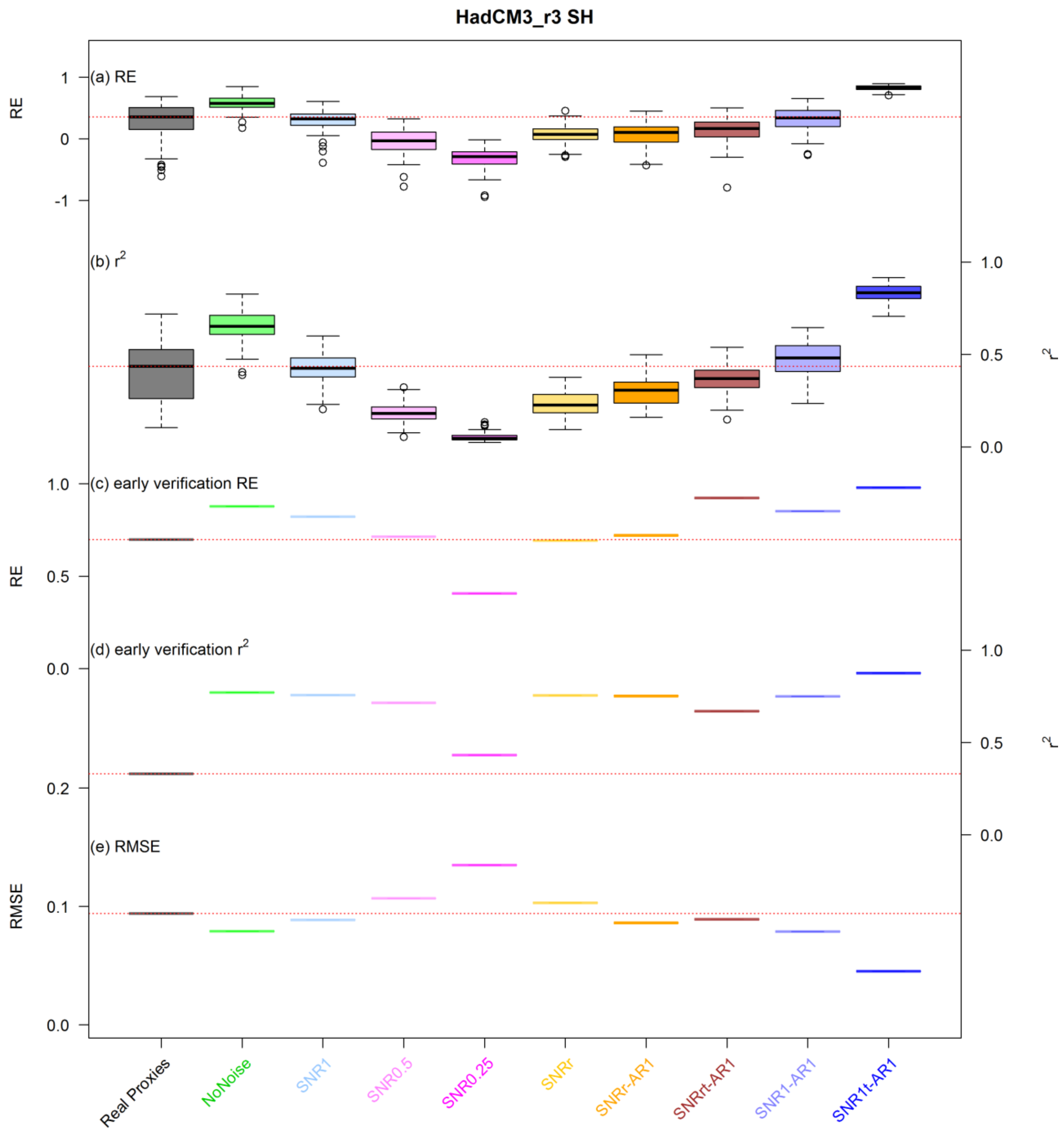


*Figure S60: Same as Figure S10 but for the HadCM3_r3 simulation only.*

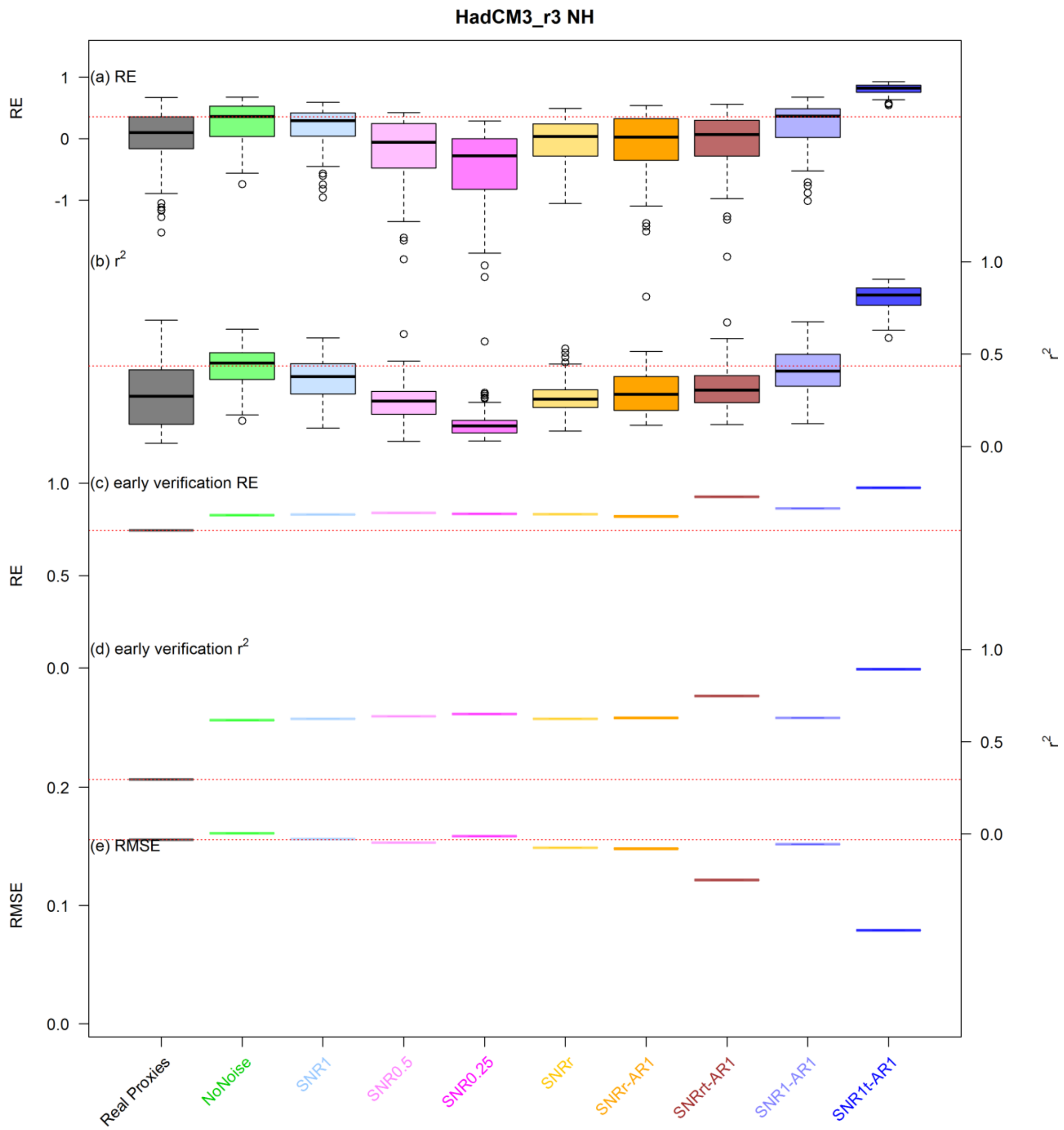


*Figure S61: Same as Figure S11 but for the HadCM3_r3 simulation only.*

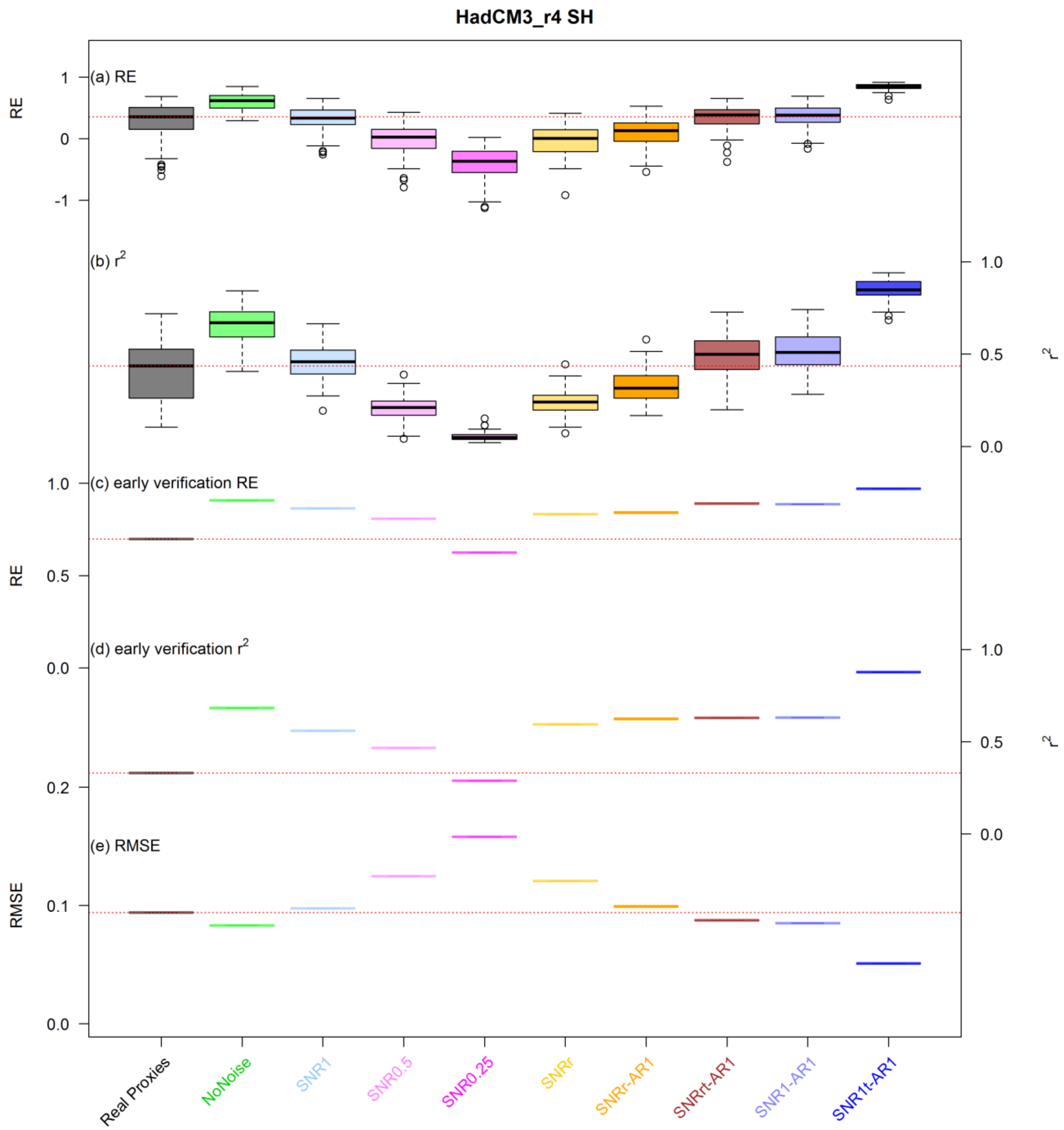


*Figure S62: Same as Figure S10 but for the HadCM3_r4 simulation only.*

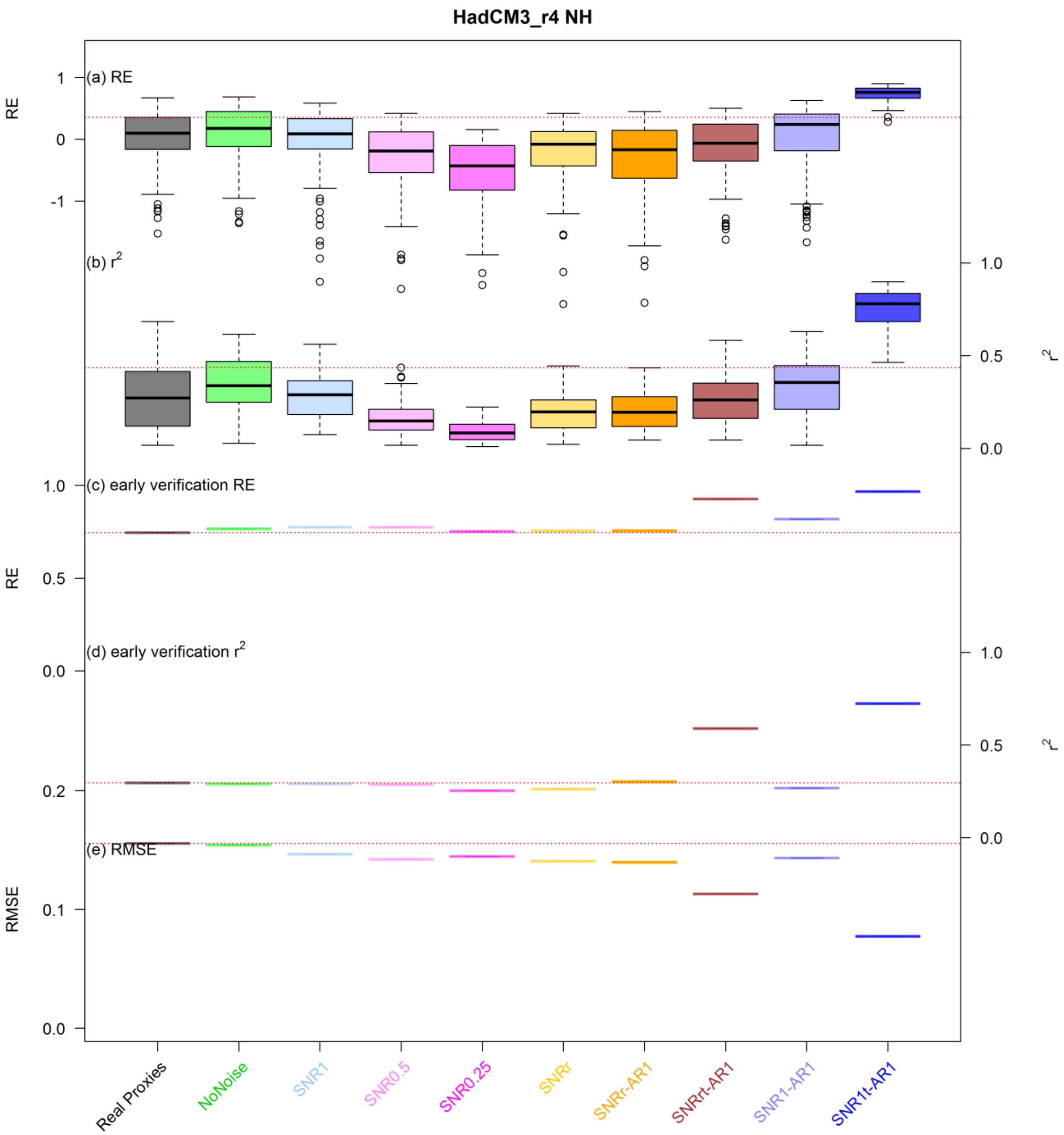


*Figure S63: Same as Figure S11 but for the HadCM3_r4 simulation only.*

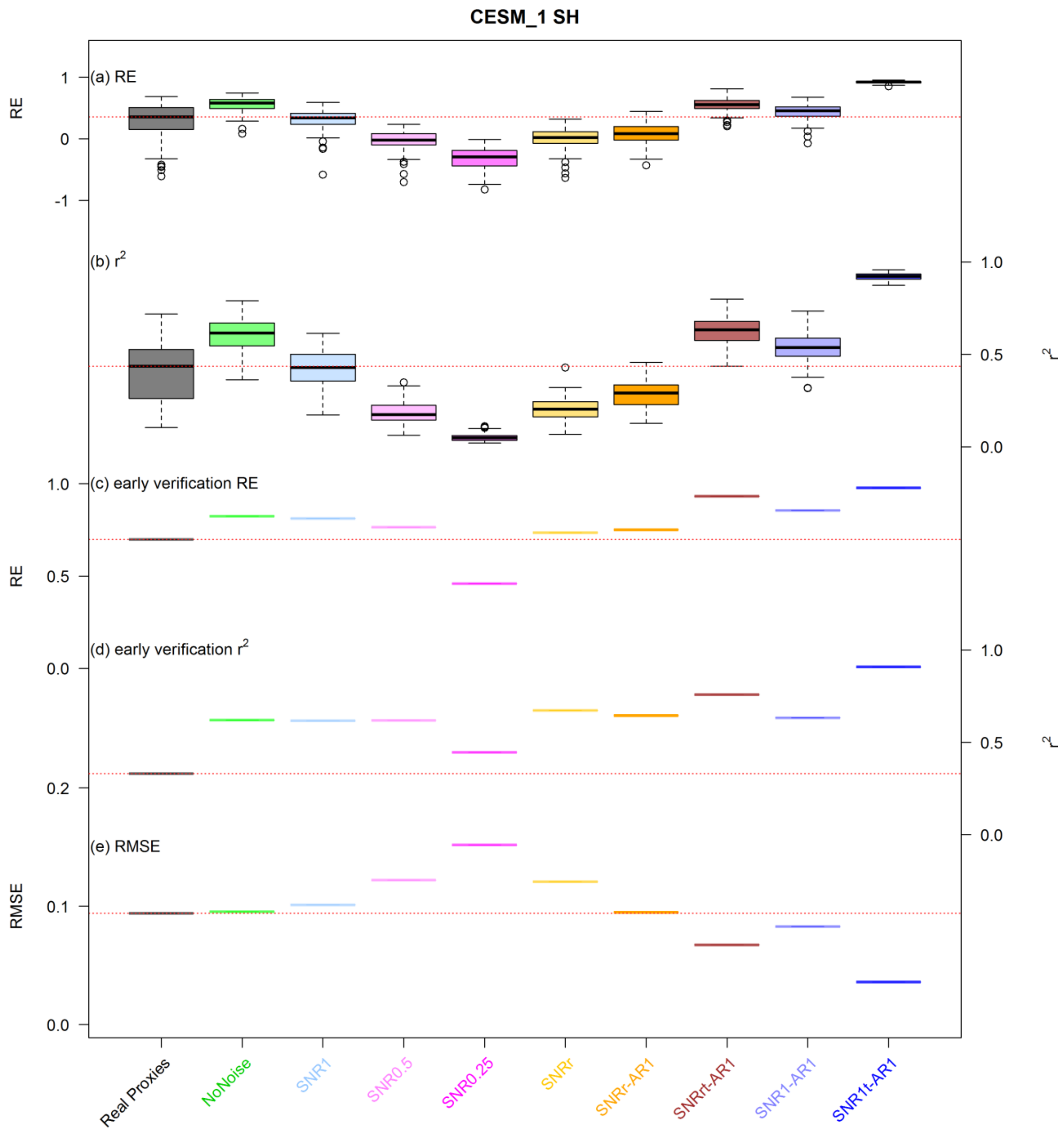


*Figure S64: Same as Figure S10 but for the CESM1-CAM5 (member 1) simulation only.*

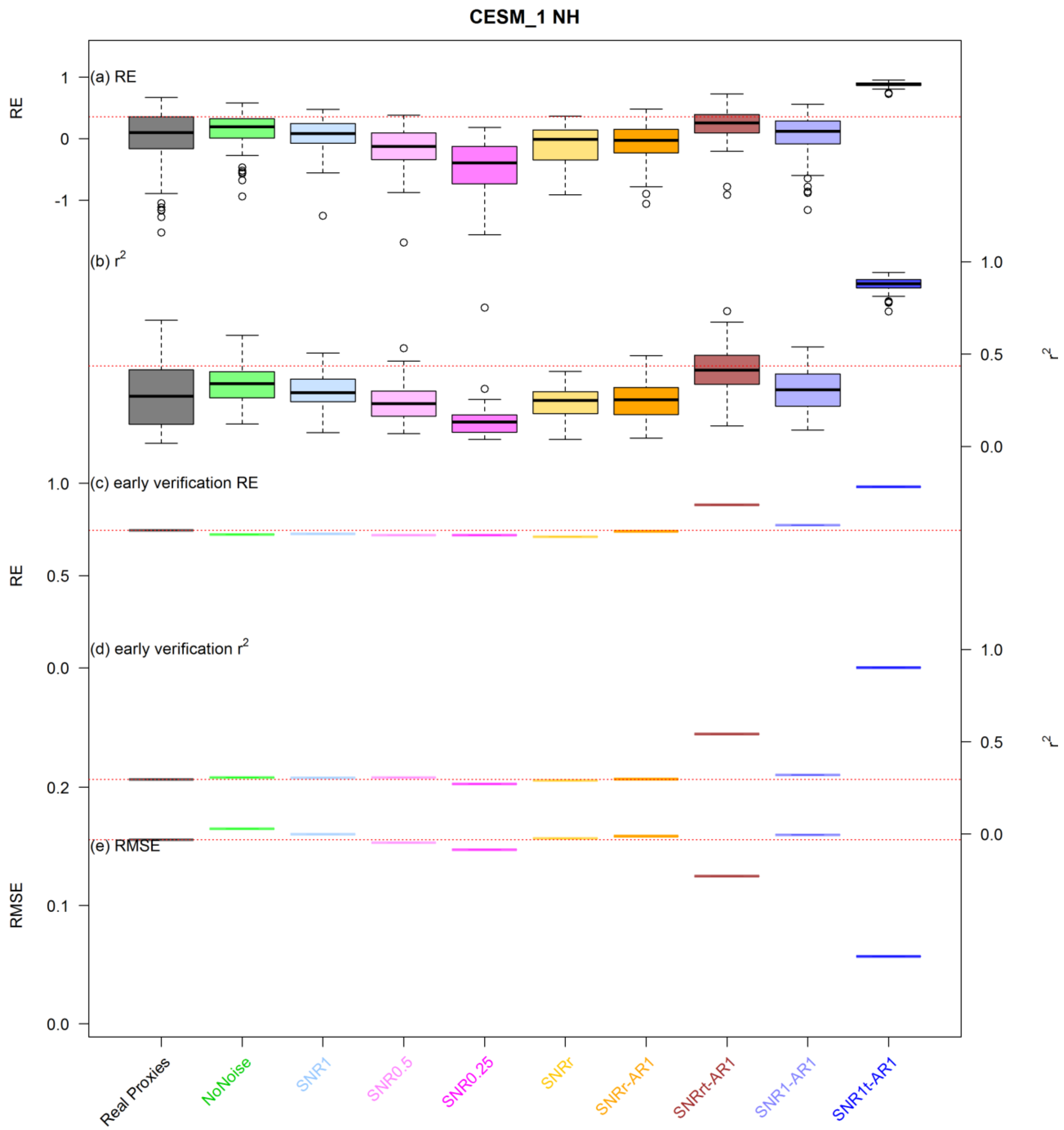


*Figure S65: Same as Figure S11 but for the CESM1-CAM5 (member 1) simulation only.*

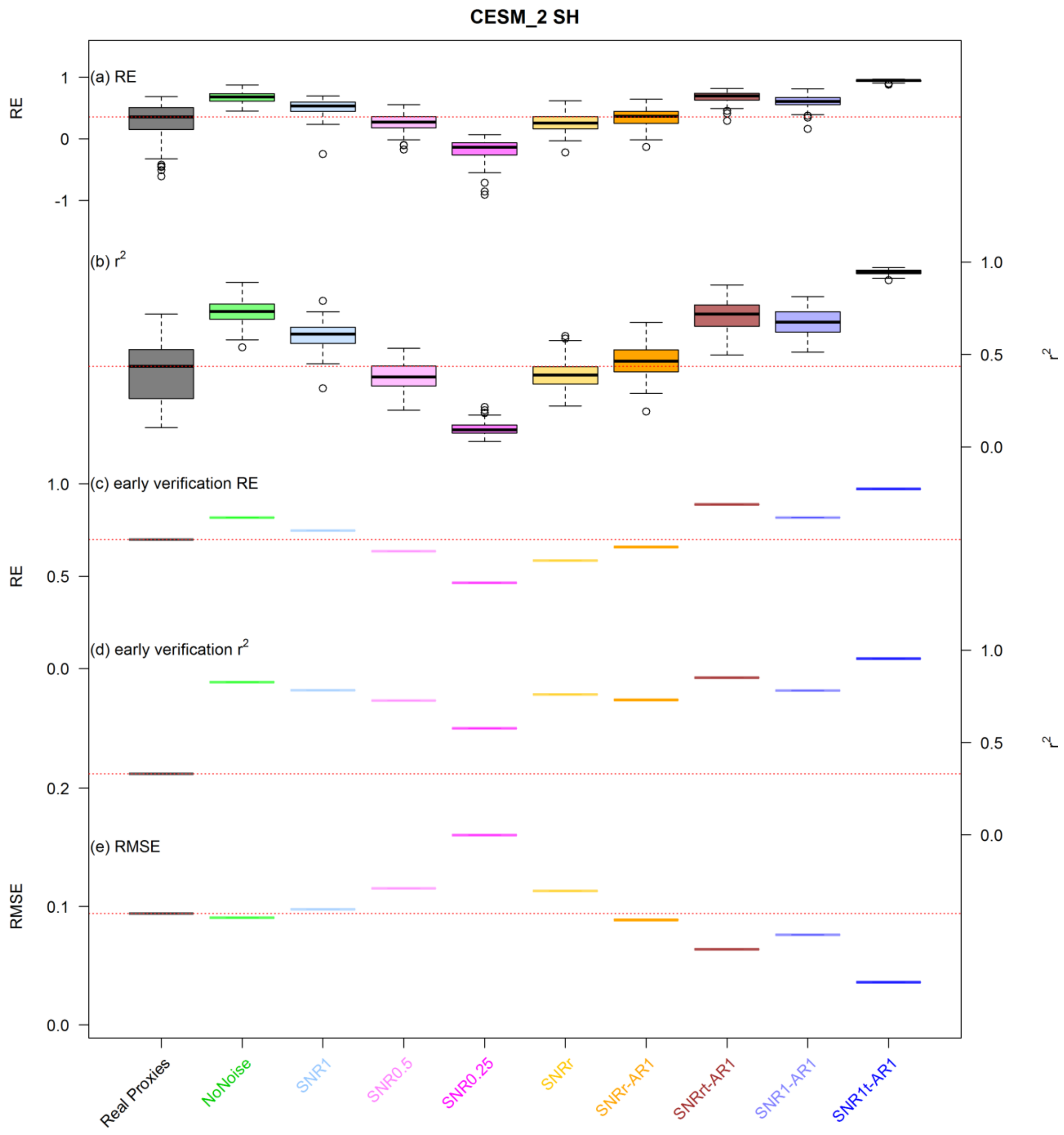


*Figure S66: Same as Figure S10 but for the CESM1 (member 2) simulation only.*

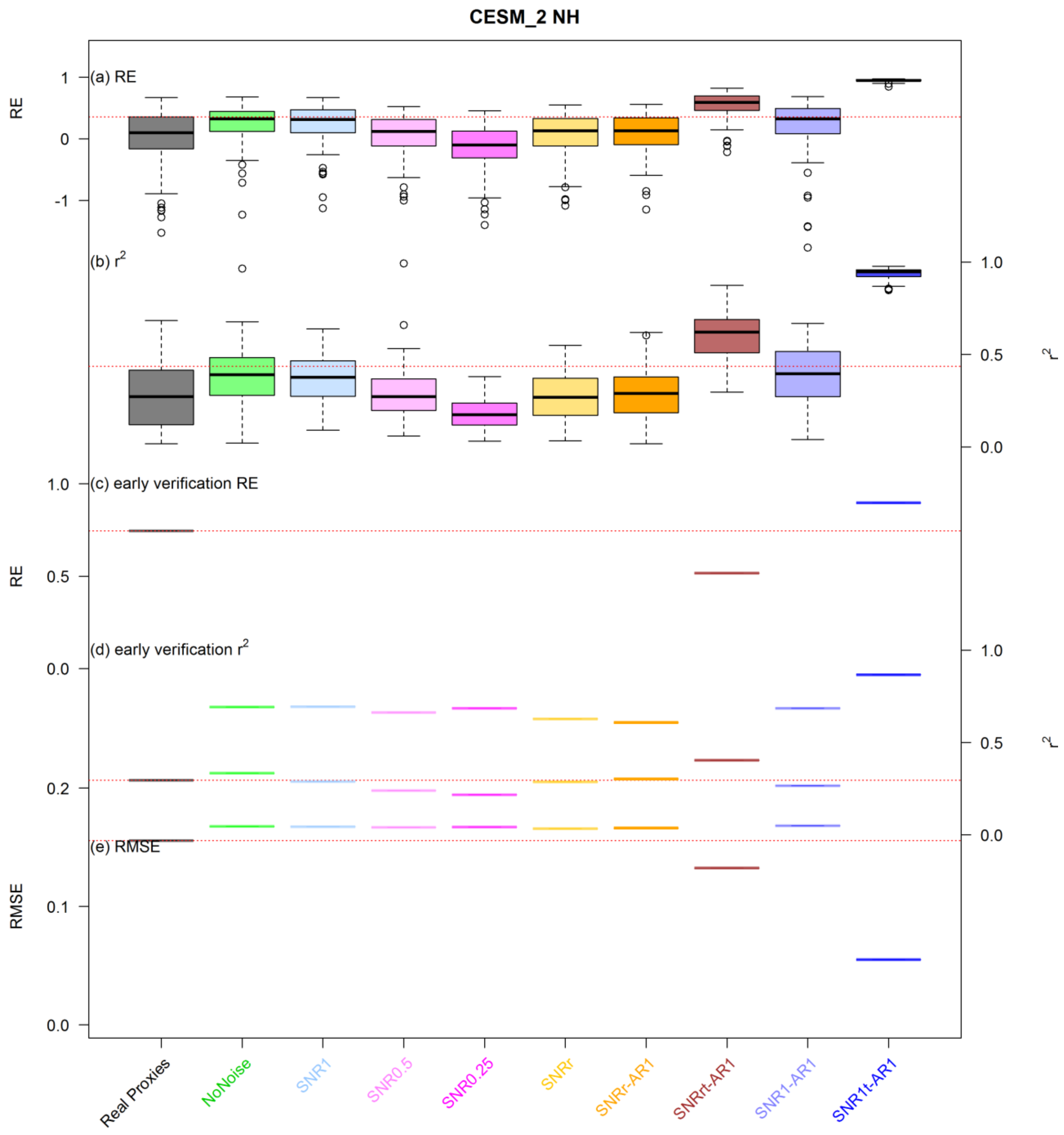


*Figure S67: Same as Figure S11 but for the CESM1-CAM5 (member 2) simulation only.*

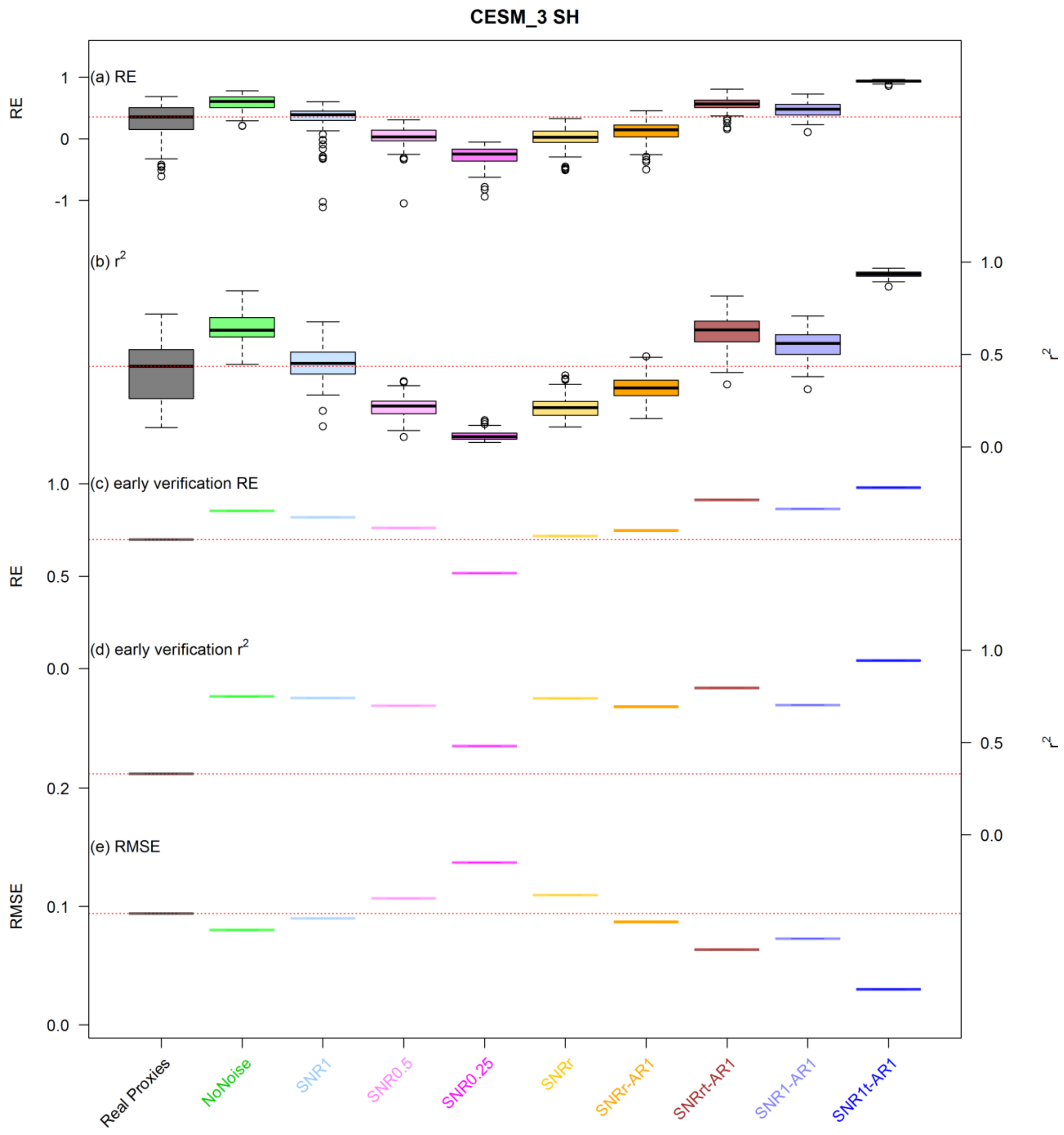


*Figure S68: Same as Figure S10 but for the CESM1-CAM5 (member 3) simulation only.*

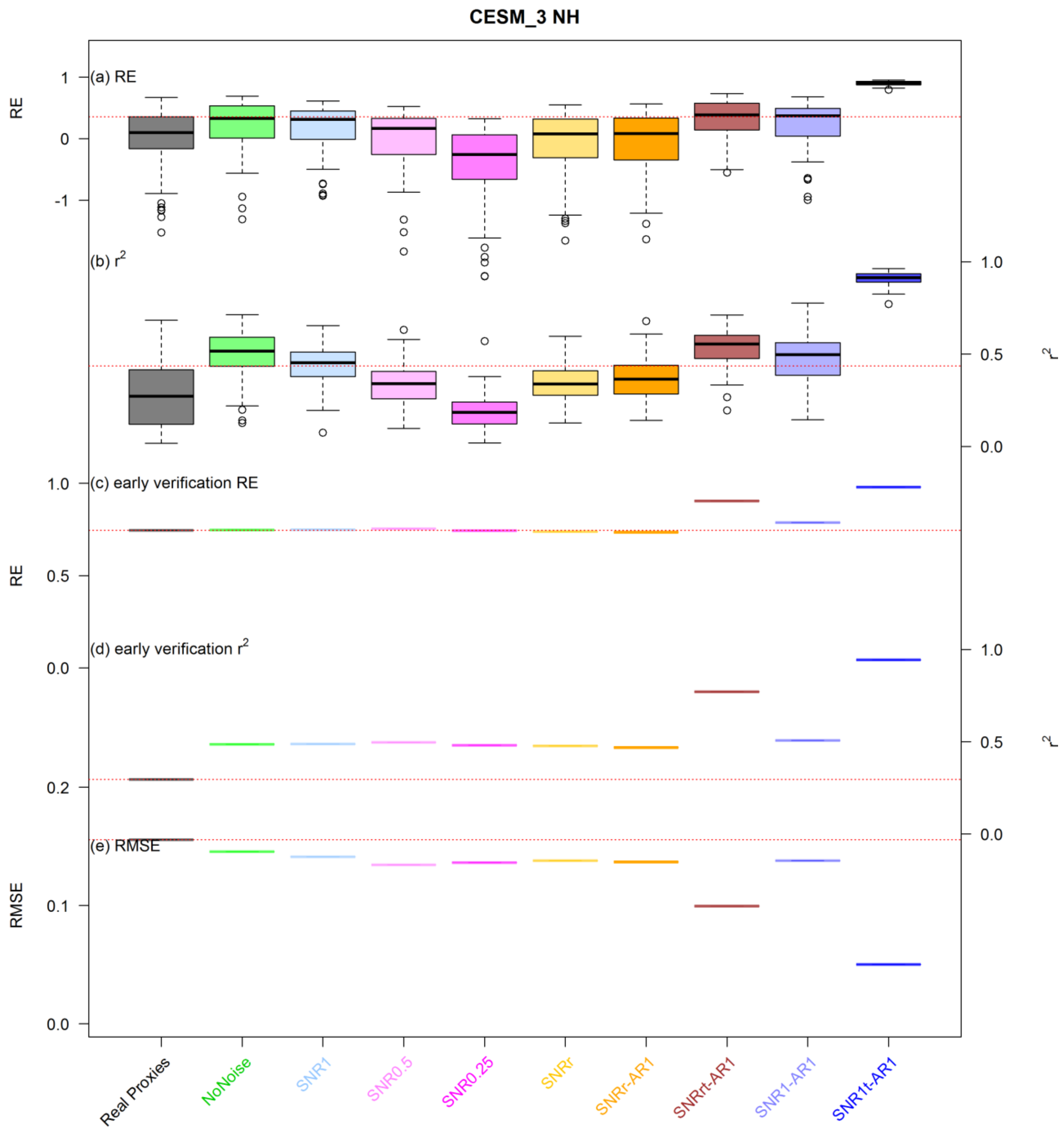


*Figure S69: Same as Figure S11 but for the CESM1-CAM5 (member 3) simulation only.*

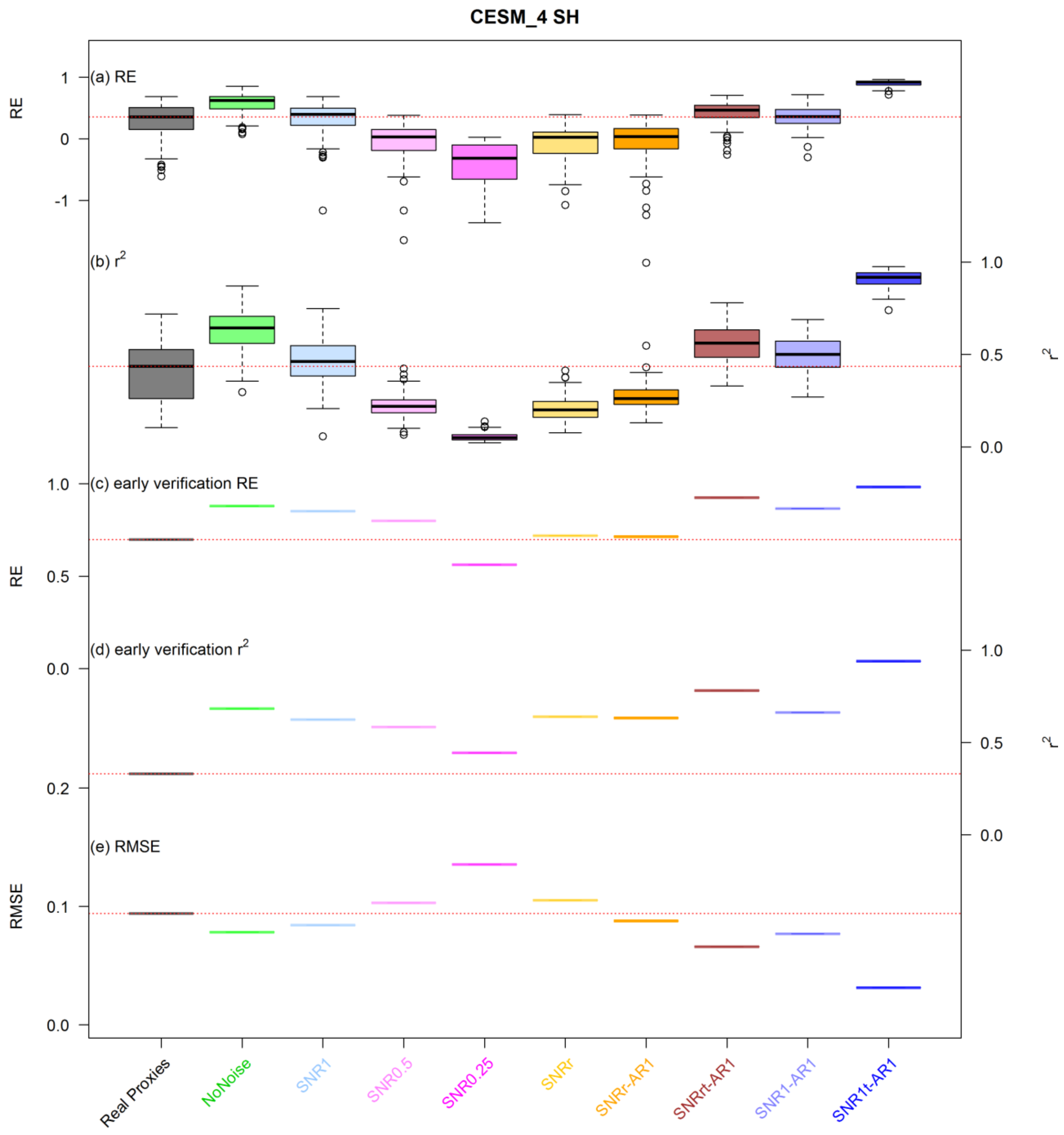


*Figure S70: Same as Figure S10 but for the CESM1-CAM5 (member 4) simulation only.*

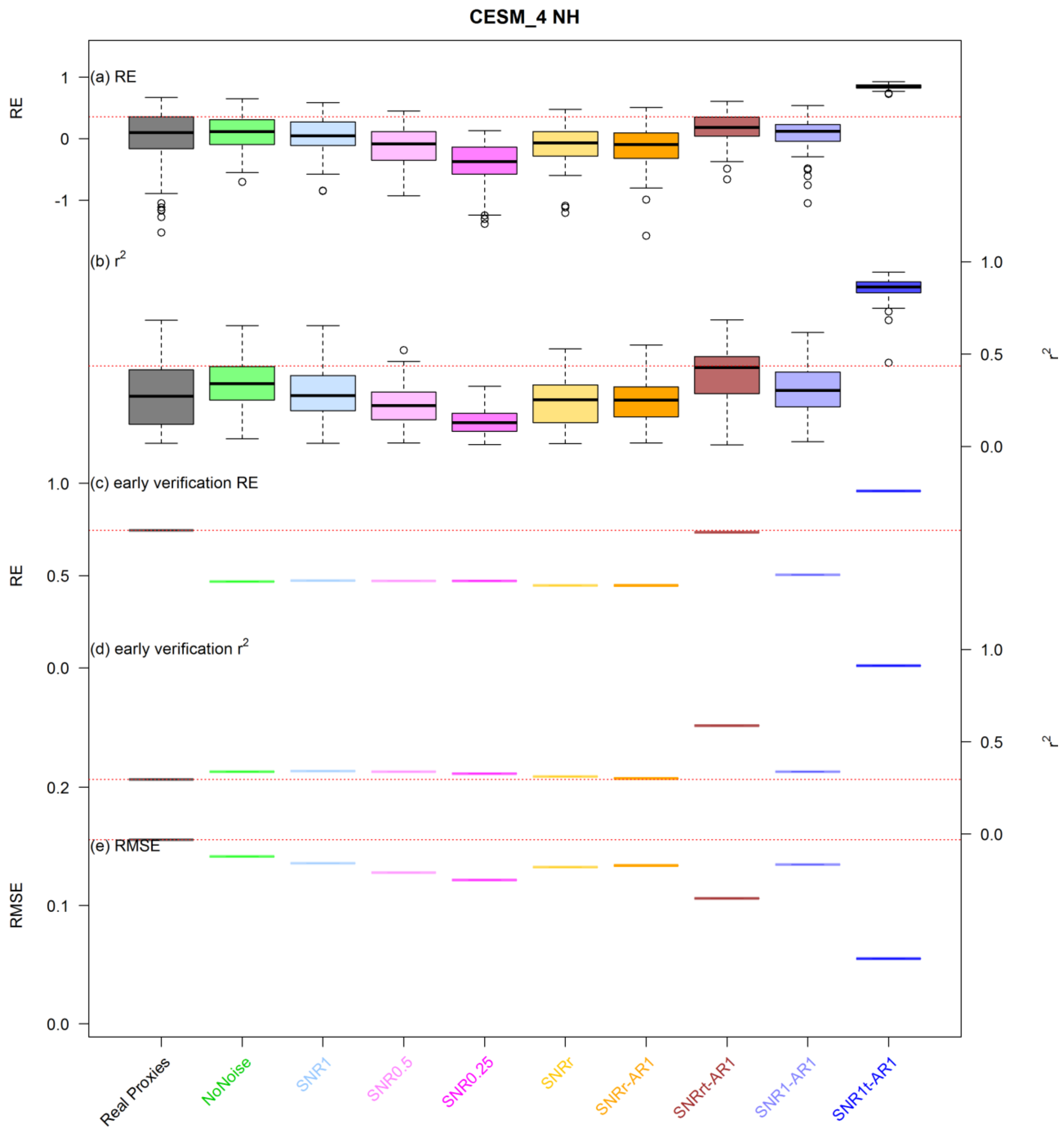


*Figure S71: Same as Figure S11 but for the CESM1-CAM5 (member 4) simulation only.*

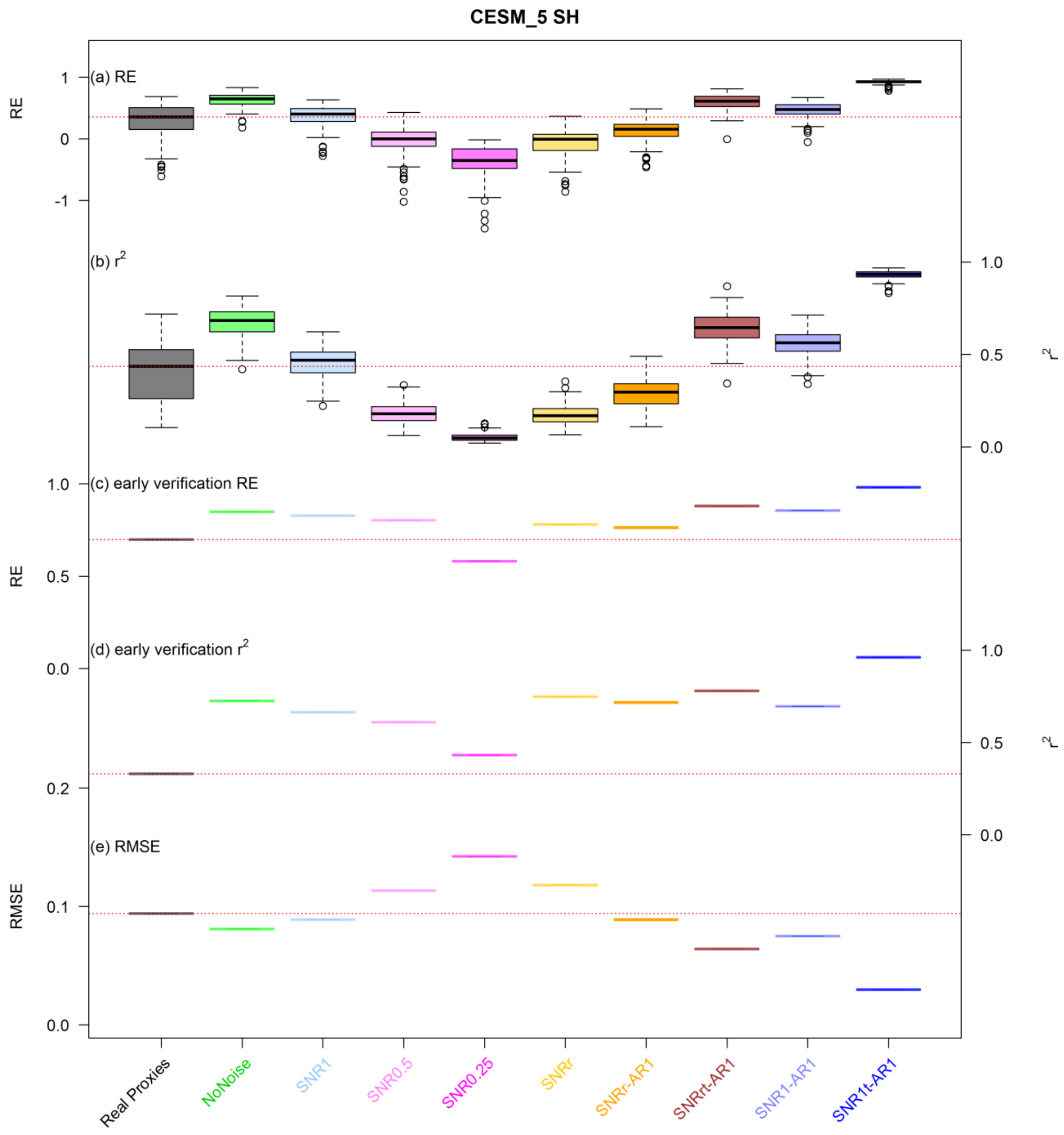


*Figure S72: Same as Figure S10 but for the CESM1-CAM5 (member 5) simulation only.*

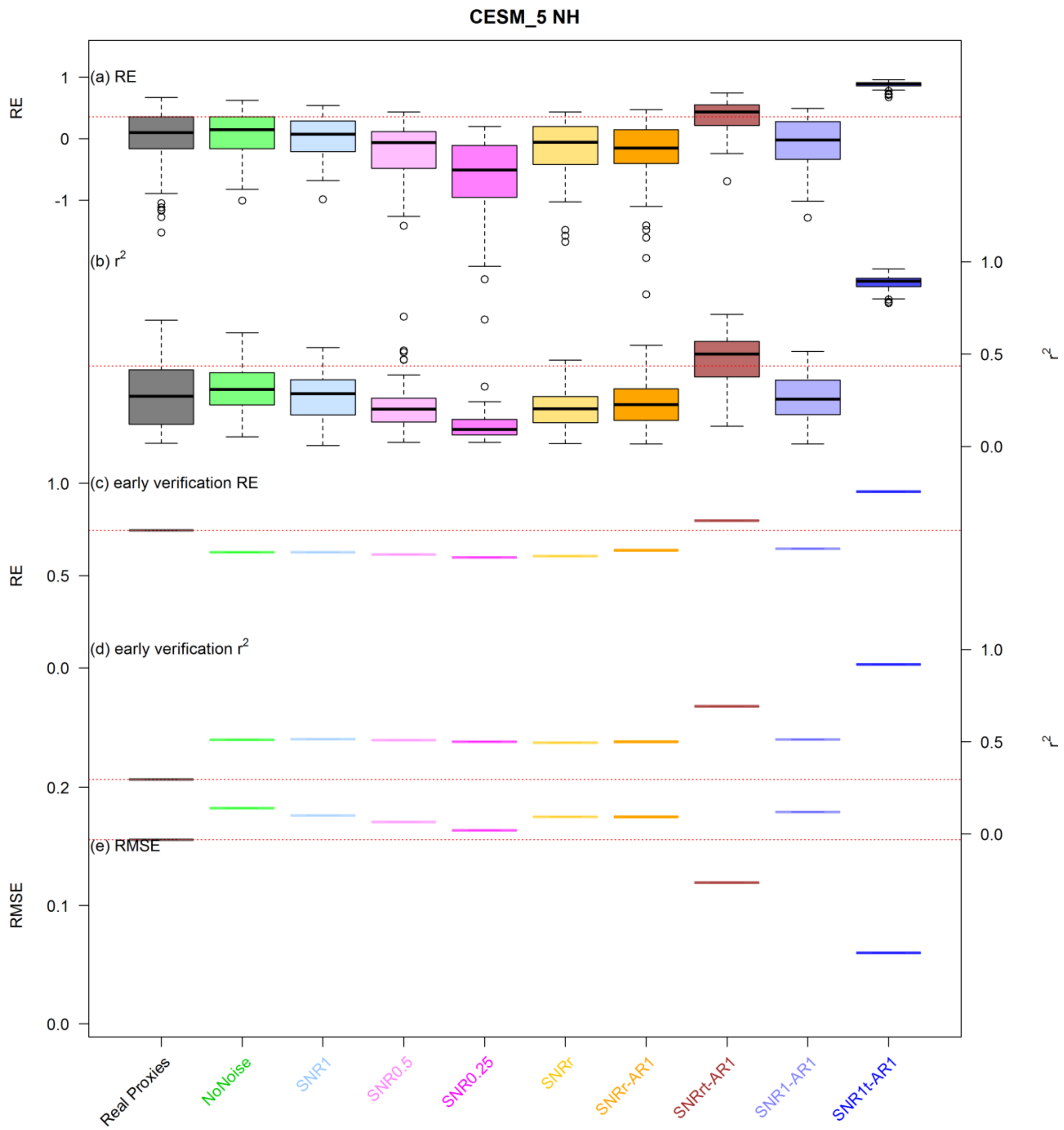


*Figure S73: Same as Figure S11 but for the CESM1-CAM5 (member 5) simulation only.*

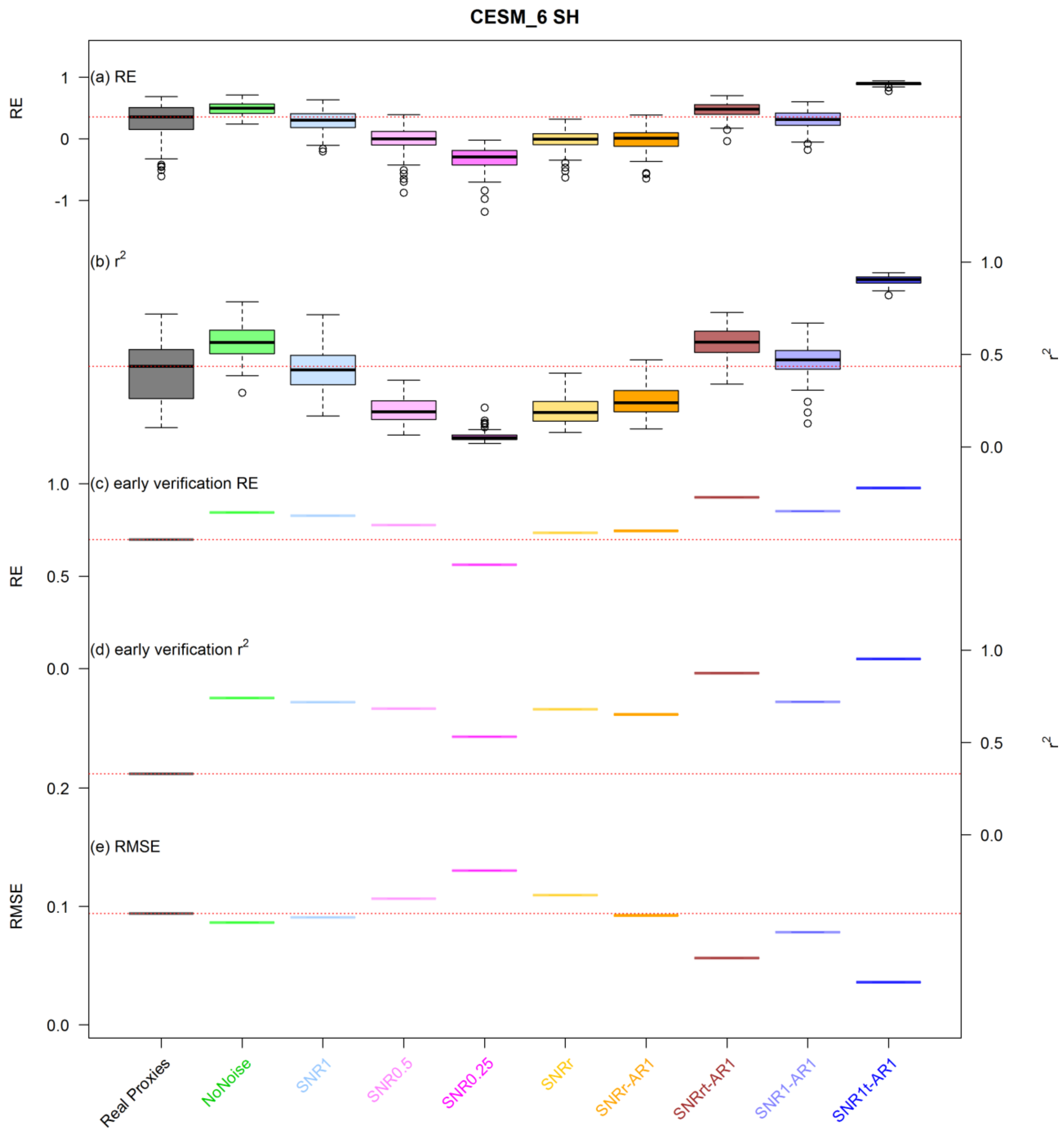


*Figure S74: Same as Figure S10 but for the CESM1-CAM5 (member 6) simulation only.*

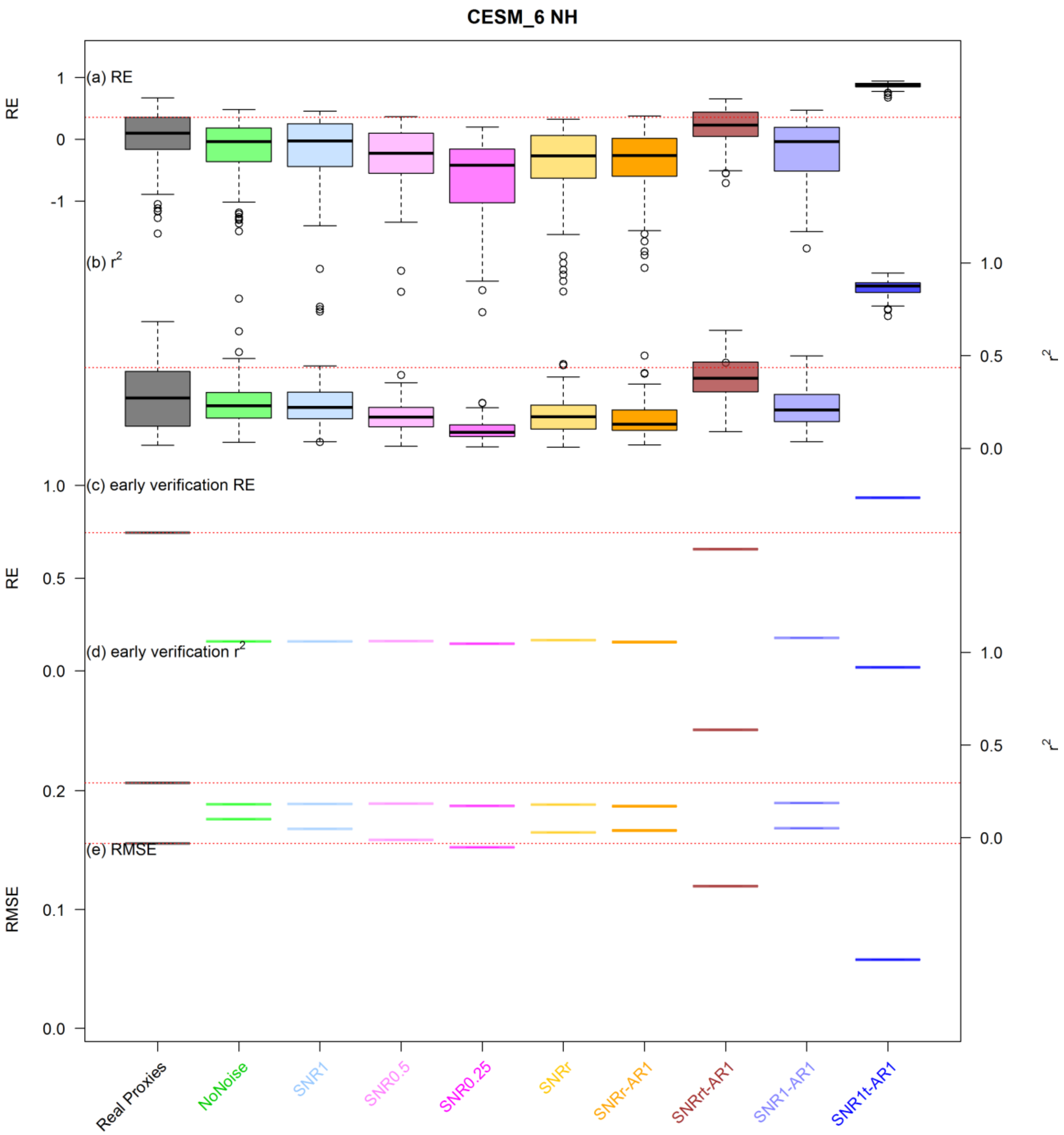


*Figure S75: Same as Figure S11 but for the CESM1-CAM5 (member 6) simulation only.*

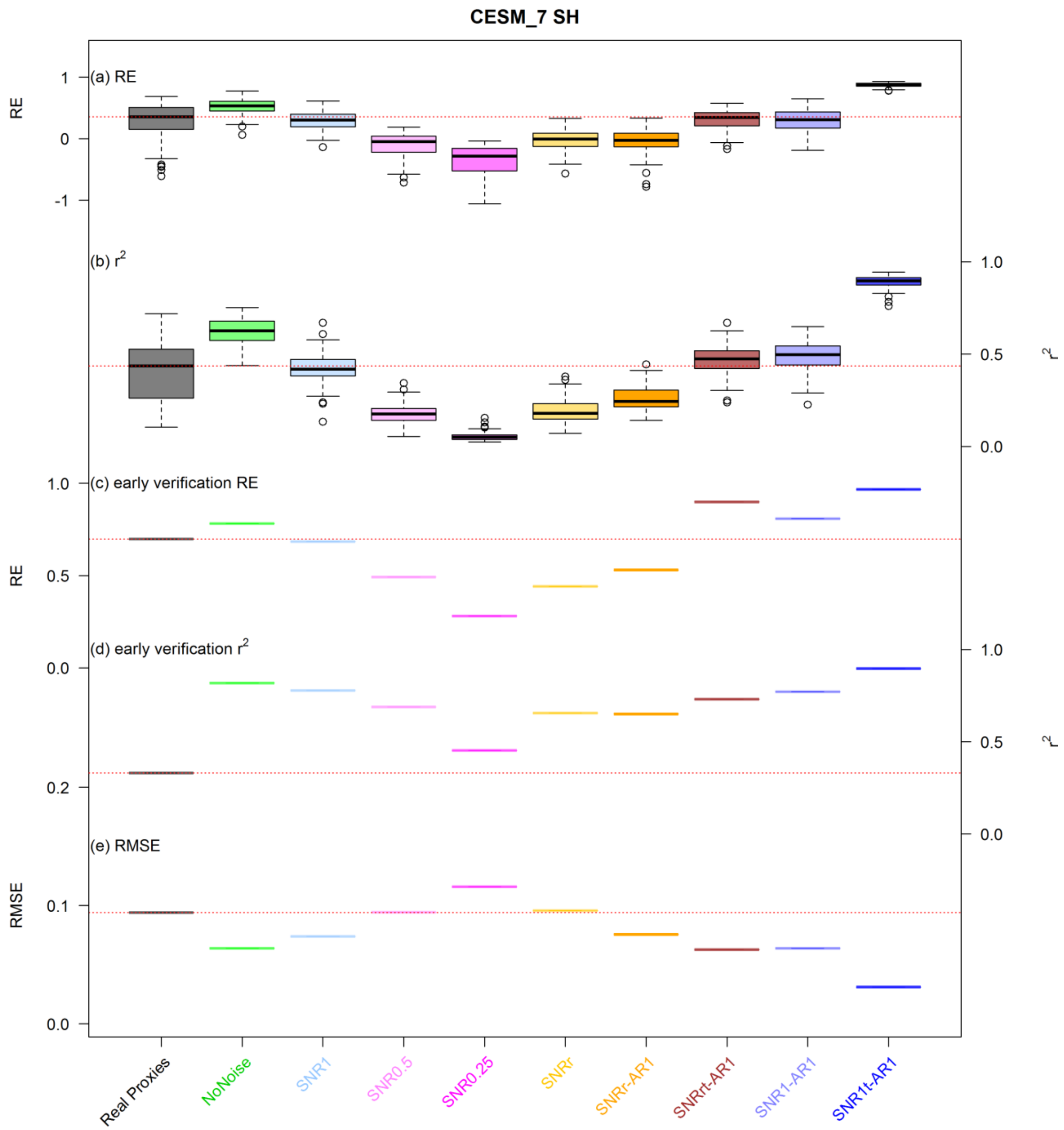


*Figure S76: Same as Figure S10 but for the CESM1-CAM5 (member 7) simulation only.*

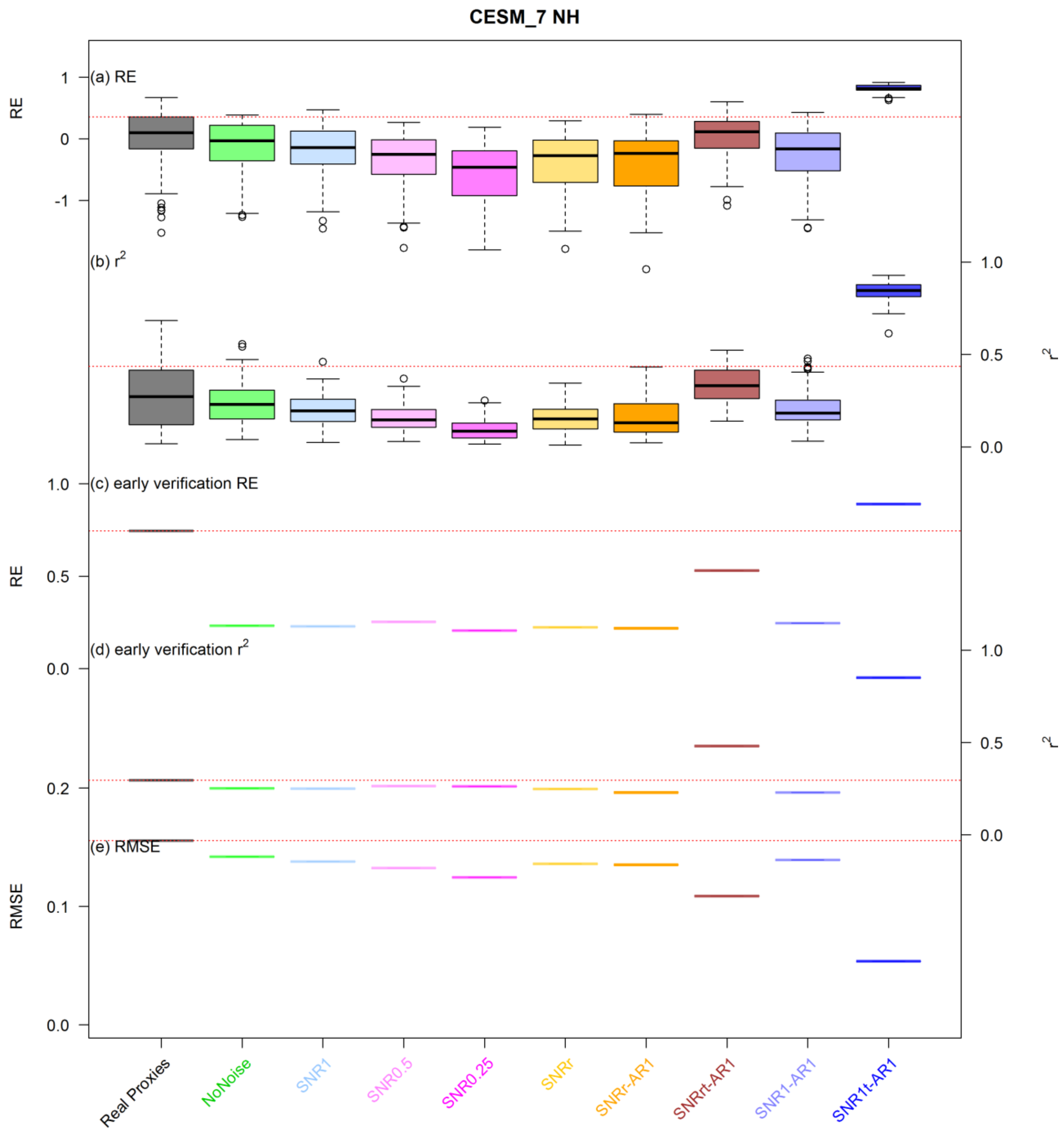


*Figure S77: Same as Figure S11 but for the CESM1-CAM5 (member 7) simulation only.*

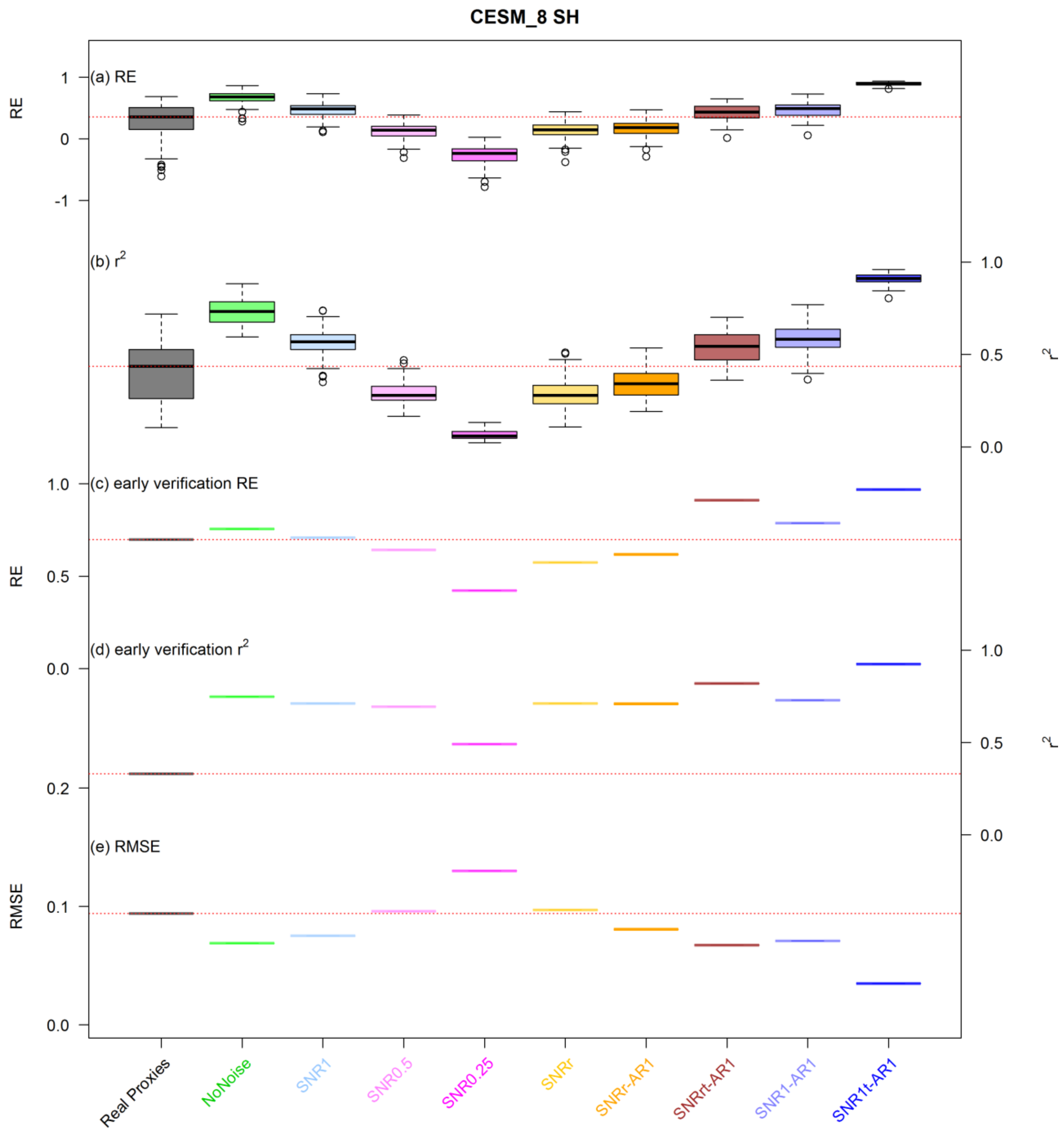


*Figure S78: Same as Figure S10 but for the CESM1-CAM5 (member 8) simulation only.*

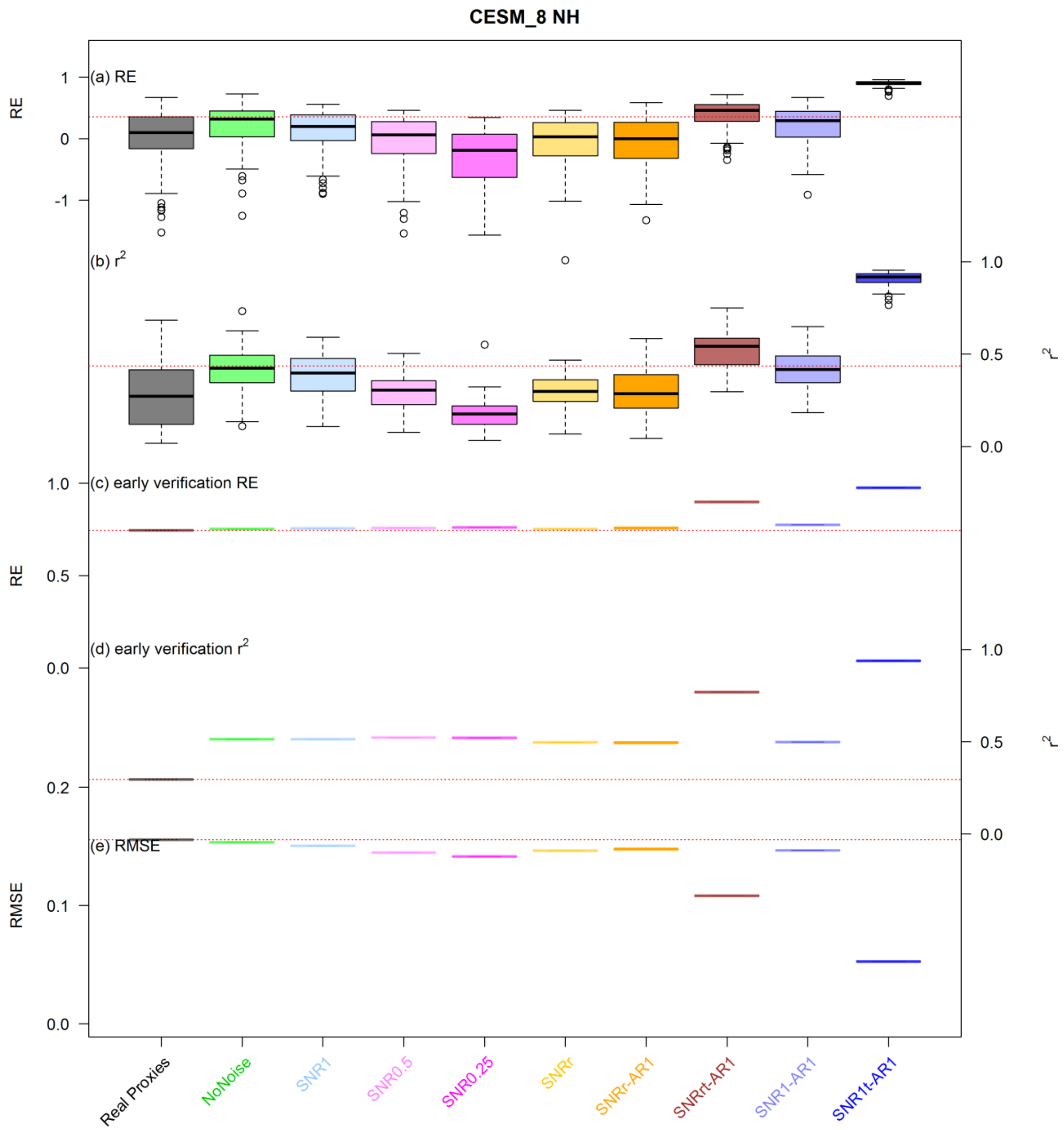


*Figure S79: Same as Figure S11 but for the CESM1-CAM5 (member 8) simulation only.*

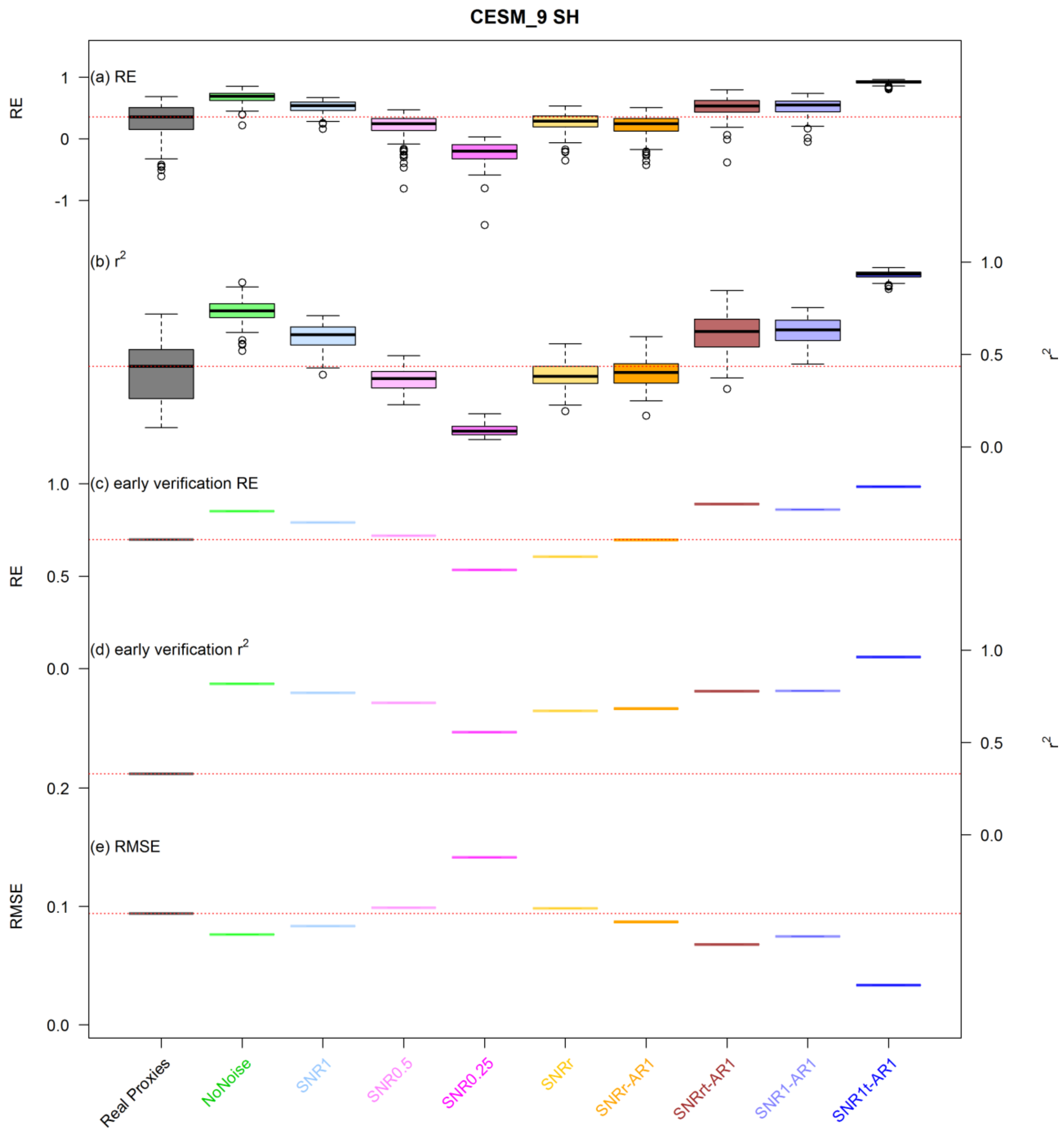


*Figure S80: Same as Figure S10 but for the CESM1-CAM5 (member 9) simulation only.*

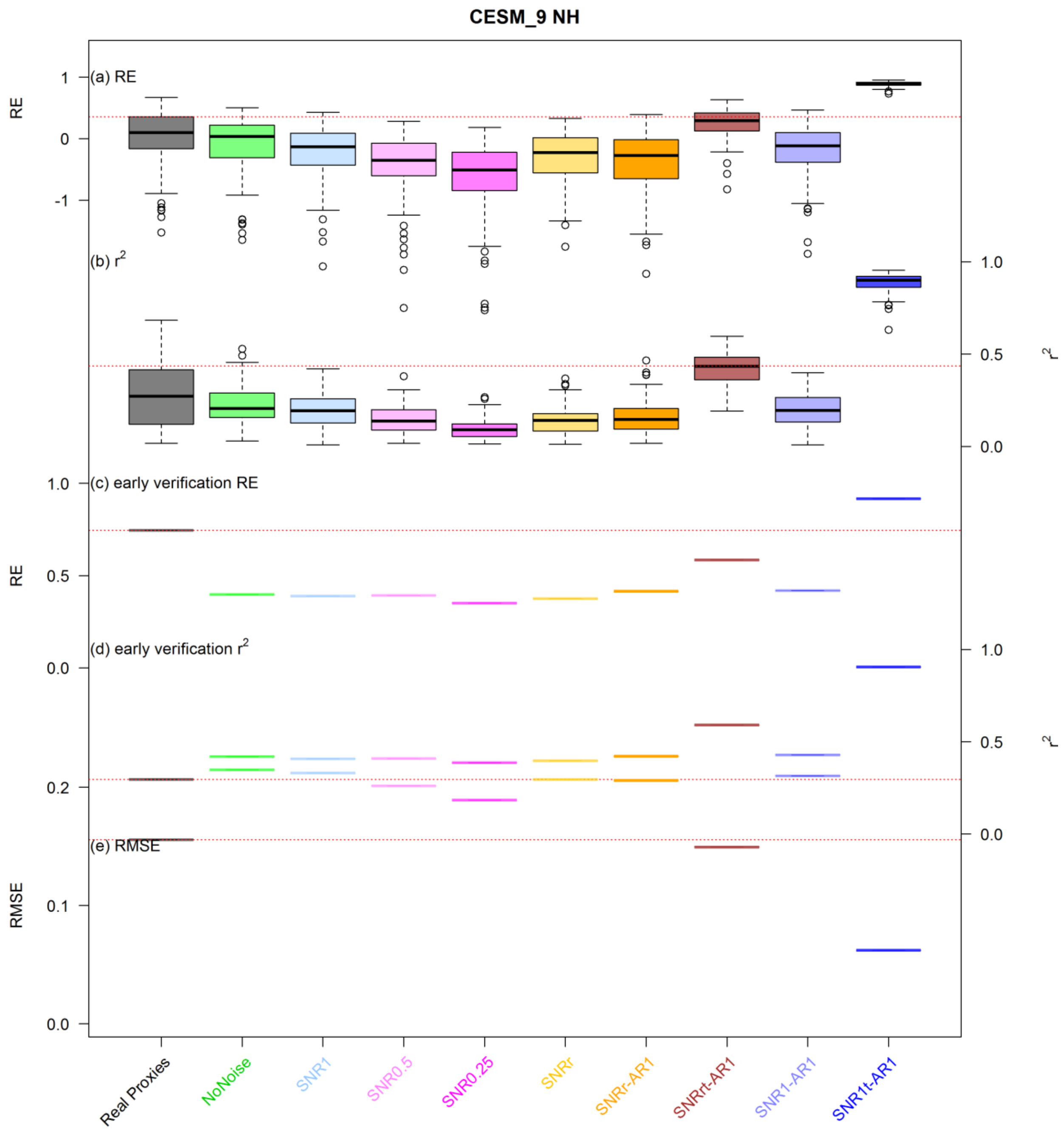


*Figure S81: Same as Figure S11 but for the CESM1-CAM5 (member 9) simulation only.*

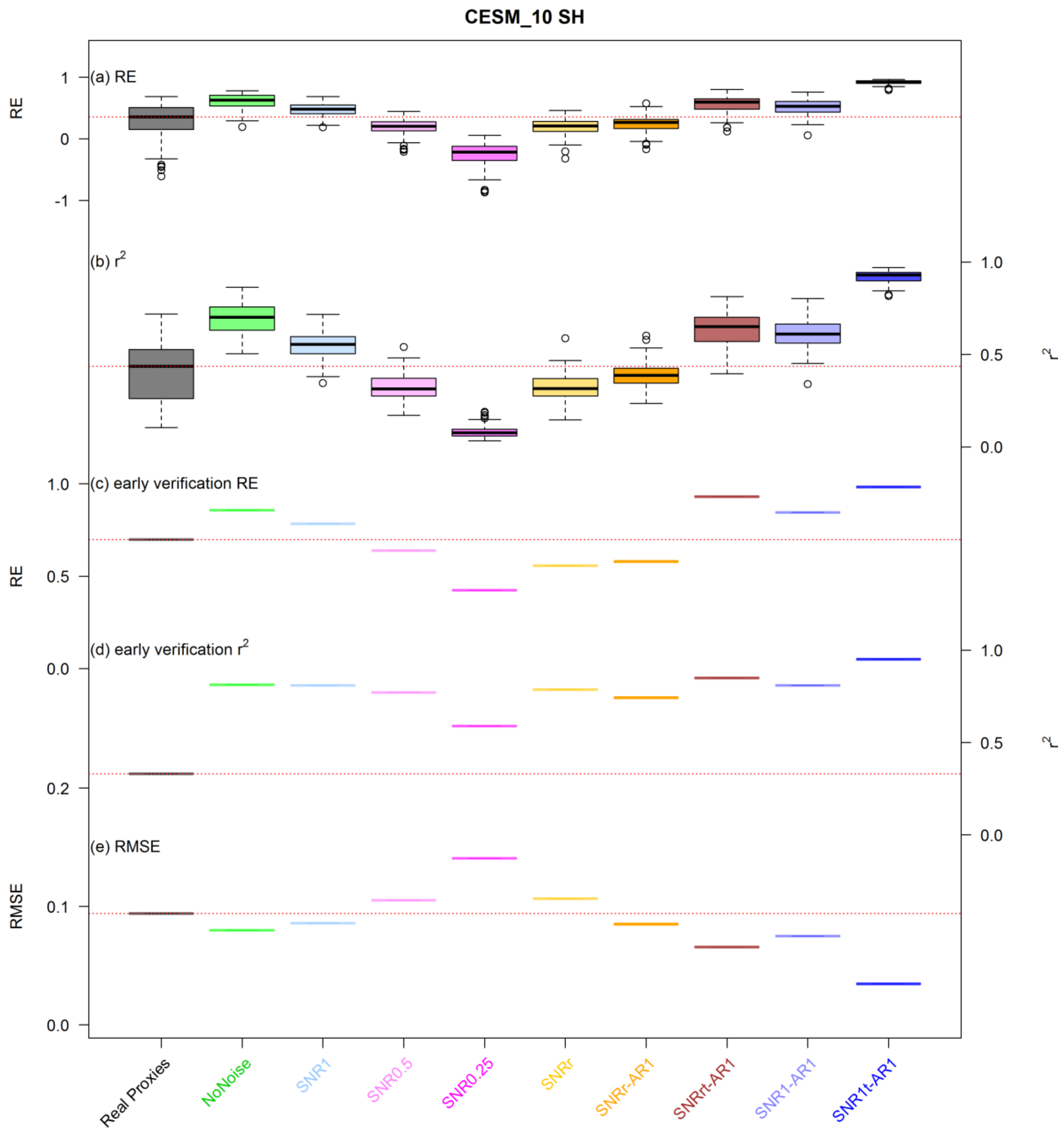


*Figure S82: Same as Figure S10 but for the CESM1-CAM5 (member 10) simulation only.*

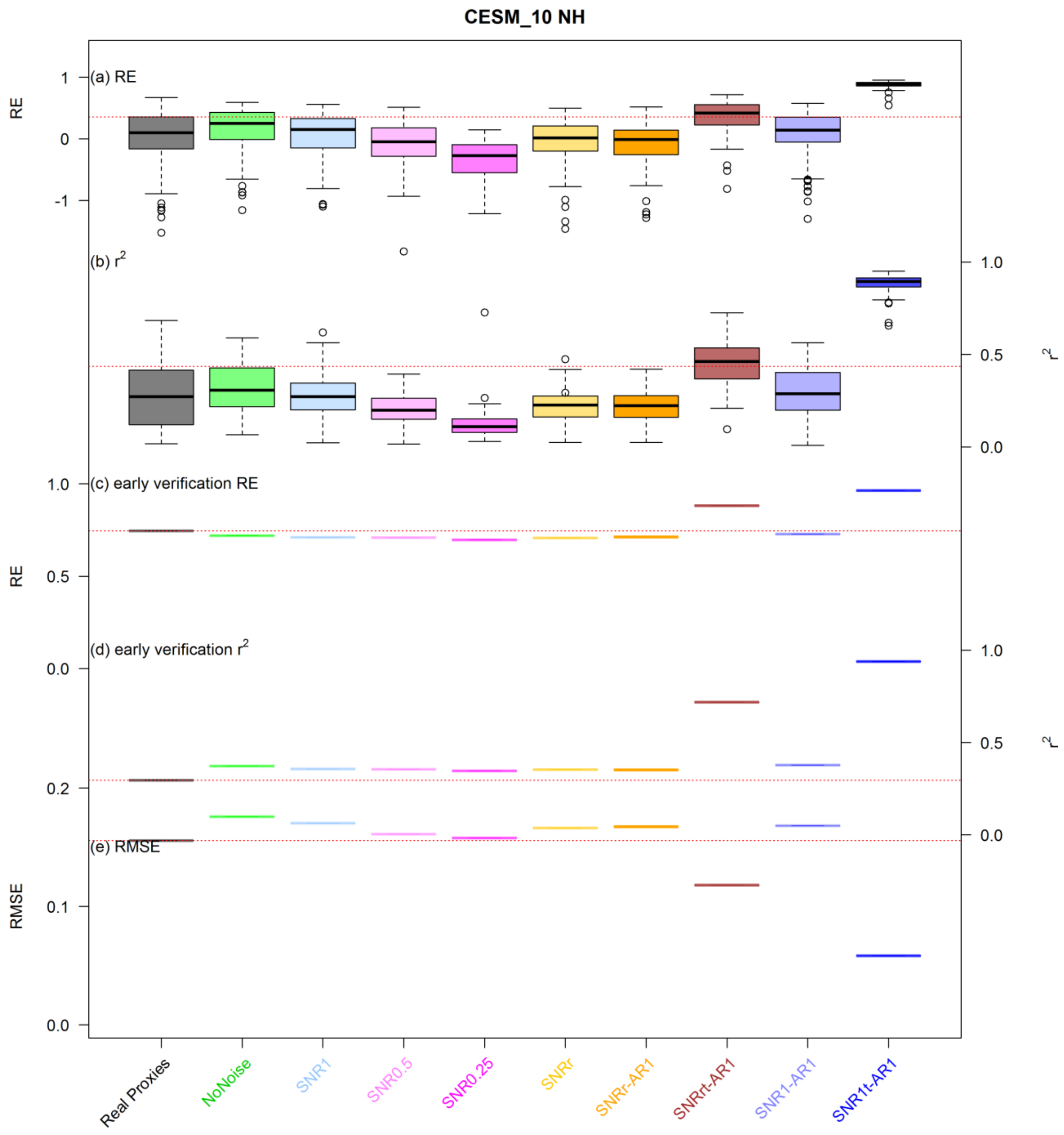


*Figure S83: Same as Figure S11 but for the CESM1-CAM5 (member 10) simulation only.*

## 13. References


1. Wilson, R. *et al.* Last millennium northern hemisphere summer temperatures from tree rings: Part I: The long term context. *Quat. Sci. Rev.* **134,** 1–18 (2016).
2. Neukom, R. *et al.* Inter-hemispheric temperature variability over the past millennium. *Nat. Clim Change* **4,** 362–367 (2014).
3. Frank, D. C. *et al.* Ensemble reconstruction constraints on the global carbon cycle sensitivity to climate. *Nature* **463,** 527–530 (2010).
4. PAGES2k Consortium, P. A global multiproxy database for temperature reconstructions of the Common Era. *Sci. Data* **4,** sdata201788 (2017).
5. Smerdon, J. E. Climate models as a test bed for climate reconstruction methods: pseudoproxy experiments. *Wiley Interdiscip. Rev. Clim. Change* **3,** 63–77 (2012).
6. Wang, J., Emile-Geay, J., Guillot, D., Smerdon, J. E. & Rajaratnam, B. Evaluating climate field reconstruction techniques using improved emulations of real-world conditions. *Clim. Past* **10,** 1–19 (2014).
7. Steiger, N. & Hakim, G. Multi-timescale data assimilation for atmosphere–ocean state estimates. *Clim Past* **12,** 1375–1388 (2016).
8. Christiansen, B., Schmith, T. & Thejll, P. A Surrogate Ensemble Study of Climate Reconstruction Methods: Stochasticity and Robustness. *J. Clim.* **22,** 951–976 (2009).
9. Gómez-Navarro, J. J., Werner, J., Wagner, S., Luterbacher, J. & Zorita, E. Establishing the skill of climate field reconstruction techniques for precipitation with pseudoproxy experiments. *Clim. Dyn.* **45,** 1395–1413 (2015).
10. Steiger, N. J. & Smerdon, J. E. A pseudoproxy assessment of data assimilation for reconstructing the atmosphere–ocean dynamics of hydroclimate extremes. *Clim Past* **13,** 1435–1449 (2017).

11. Tolwinski-Ward, S. E., Evans, M. N., Hughes, M. K. & Anchukaitis, K. J. An efficient forward model of the climate controls on interannual variation in tree-ring width. *Clim. Dyn.* **36,** 2419–2439 (2011).

12. Thompson, D. M., Ault, T. R., Evans, M. N., Cole, J. E. & Emile-Geay, J. Comparison of observed and simulated tropical climate trends using a forward model of coral δ18O. *Geophys. Res. Lett.* **38,** L14706 (2011).

13. Hanhijärvi, S., Tingley, M. P. & Korhola, A. Pairwise comparisons to reconstruct mean temperature in the Arctic Atlantic Region over the last 2,000 years. *Clim. Dyn.* **41,** 2039–2060 (2013).

14. Li, B., Nychka, D. W. & Ammann, C. M. The Value of Multiproxy Reconstruction of Past Climate. *J. Am. Stat. Assoc.* **105,** 883–895 (2010).
